# Nanopore Workflow for Grapevine Viroid Surveillance in Kazakhstan: Bypassing rRNA Depletion Through Non-Canonical Priming

**DOI:** 10.3390/pathogens14080782

**Published:** 2025-08-06

**Authors:** Karlygash P. Aubakirova, Zhibek N. Bakytzhanova, Akbota Rakhatkyzy, Laura S. Yerbolova, Natalya P. Malakhova, Nurbol N. Galiakparov

**Affiliations:** 1 Laboratory of Biotechnology and Molecular Genetics, M. Aitkhozhin Institute of Molecular Biology and Biochemistry, Almaty 050012, Kazakhstan; karlygashaubakirova9@gmail.com (K.P.A.); bakytzhanovazhibek@gmail.com (Z.N.B.); akbotarahatkyzy1@gmail.com (A.R.); yerbolova.laura7@gmail.com (L.S.Y.); 2 Laboratory of Plant Bioengineering, M. Aitkhozhin Institute of Molecular Biology and Biochemistry, Almaty 050012, Kazakhstan; malakhova.natalie@gmail.com

**Keywords:** grapevine viroids, hop stunt viroid, grapevine yellow speckle viroid 1, nanopore sequencing, total RNA, viroid detection, *Vitis vinifera*, Kazakhstan

## Abstract

Grapevine (*Vitis vinifera* L.) cultivation is an important agricultural sector worldwide. Its expansion into new areas, like Kazakhstan, brings significant phytosanitary risks. Viroids, such as grapevine yellow speckle viroid 1 (GYSVd-1) and hop stunt viroid (HSVd), are RNA pathogens that threaten vineyard productivity. They can cause a progressive decline through latent infections. Traditional diagnostic methods are usually targeted and therefore not suitable for thorough surveillance. In contrast, modern high-throughput sequencing (HTS) methods often face challenges due to their high costs and complicated sample preparation, such as ribosomal RNA (rRNA) depletion. This study introduces a simplified diagnostic workflow that overcomes these barriers. We utilized the latest Oxford Nanopore V14 cDNA chemistry, which is designed to prevent internal priming, by substituting a targeted oligo(dT)VN priming strategy to facilitate the sequencing of non-polyadenylated viroids from total RNA extracts, completely bypassing the rRNA depletion step and use of random oligonucleotides for c DNA synthesis. This method effectively detects and identifies both GYSVd-1 and HSVd. This workflow significantly reduces the time, cost, and complexity of HTS-based diagnostics. It provides a powerful and scalable tool for establishing strong genomic surveillance and phytosanitary certification programs, which are essential for supporting the growing viticulture industry in Kazakhstan.

## 1. Introduction

Grapevine (*Vitis vinifera* L.) is one of the most important fruit crops in the world, supporting a wine and table grape industry worth tens of billions of dollars each year [1]. However, grapevines are vulnerable to pathogens that establish systemic infections due to their vegetative propagation [2]. Viroids are particularly concerning among these pathogens. They are small, single-stranded, circular RNA molecules that do not encode proteins like viruses [3]. These agents can replicate independently by hijacking the host’s cellular machinery. Their harmful effects relate directly to the structure and interactions of their RNA genomes [4].

Several viroids can infect grapevines. GYSVd-1, from the genus *Apscaviroid*, and HSVd, from the genus *Hostuviroid*, are two of the most common [5]. Infections can cause visible symptoms like yellow spots and flecks on leaves, stunted cane growth, and lower fruit quality. These issues can affect yield and marketability. Even more concerning, many infections are either latent or asymptomatic. Under certain conditions or in tolerant grapevine varieties, infected plants might show no clear signs of disease for several years [1].

International trade and the exchange of infected, often asymptomatic propagation materials, such as cuttings, rootstocks, and scions, are the main ways these pathogens spread around the world [6]. This situation puts significant pressure on plant health and certification programs. If these latent pathogens go undetected before planting, they can create disease reservoirs in new vineyards, leading to economic losses that might not show up for years. For this reason, developing fast, sensitive, and cost-effective diagnostic tools is crucial for the industry.

Kazakhstan has a long history of viticulture, with records of grapevine cultivation dating back to the 7th century AD. Grapevines were brought in along ancient trade routes from China and Uzbekistan. After a period of decline, recent national efforts have focused on expanding Kazakhstan’s wine industry [7]. This initiative requires substantial investments in new vineyards and improvements to existing ones, often through the large-scale importation of valuable European *V. vinifera* cultivars [7,8]. The mass movement of these propagation materials makes it easy for foreign pathogens to enter areas where they were not previously found. In an industry based on long-term investments in perennial crops, introducing incurable systemic pathogens poses a serious threat. Thus, the long-term success of Kazakhstani viticulture depends on both horticultural expertise and a strong plant health framework. A key part of this framework is the ability to screen imported nursery stock, monitor local vineyards, and implement effective certification programs [9].

Pathogen diagnostics have progressed from specific tests, like Reverse Transcription-Polymerase Chain Reaction (RT-PCR), which can detect only known pathogens, to untargeted high-throughput sequencing (HTS) [10]. Traditional viroid detection methods, such as RT-PCR and Northern blotting, are reliable but labor-intensive and may require specific primers for known viroids. Therefore, they are not suitable for broad screening or discovering new variants [11]. HTS allows for comprehensive genomic surveillance. It enables the identification of the entire “virome” in a sample without prior assumptions, which is essential for biosecurity and understanding complex diseases [12]. Although HTS is powerful, established platforms are often expensive and involve complicated protocols. Newer platforms, like Oxford Nanopore Technologies (ONT), offer a promising alternative for agricultural diagnostics due to their portability, real-time data analysis, and simpler workflows, making them suitable for surveillance [13].

Although ONT is an effective platform, the rapid evolution of its sequencing methods and library preparation kits creates specific challenges. Newer kits are often specialized and optimized for certain applications, such as high-accuracy, full-length analysis of eukaryotic messenger RNAs (mRNAs). This specialization can hinder researchers focused on unique targets, like viroids, which have distinct biochemical and structural properties that do not fit standard workflows. However, two major technical challenges affect using HTS for viroid detection. First, viroid RNA makes up a small portion of the total RNA in infected plants, while host ribosomal RNA (rRNA) accounts for 80–90% of the total RNA pool [14]. Standard protocols require expensive and time-consuming rRNA depletion or dsRNA enrichment steps to avoid wasting sequencing resources on non-target molecules. Second, viroids from the *Pospiviroidae* family are circular, non-polyadenylated RNAs. This characteristic makes them incompatible with common RNA-seq methods that use oligo(dT) primers to capture the poly(A) tails of mRNAs.

This study proposes a new workflow to address these challenges. Viroids from the *Pospiviroidae* family fold into stable, rod-like secondary structures that are crucial for their biological function and have evolved to be conserved in nature. Our main hypothesis is that specific, accessible single-stranded loops within these conserved structures, which are rich in adenine (A) residues, can serve as non-canonical binding sites for the generic oligo(dT)VN primer typically used for cDNA synthesis. By starting reverse transcription from these internal A-rich regions, our approach could enable the sequencing of non-polyadenylated viroid genomes while also enriching diagnostically relevant transcripts, including viroids, viruses, and host mRNAs. This method would eliminate the need for a natural poly(A) tail and would make the separate, costly rRNA depletion step unnecessary, creating a much more efficient and affordable diagnostic process.

## 2. Materials and Methods

### 2.1. Sample Collection and Total RNA Extraction

Mature leaf samples were collected from multiple symptomatic and asymptomatic *Vitis vinifera* L. cv. Alice grapevines in the Almaty region of Kazakhstan during the 2024 growing season. From these, a total of eight representative samples (one symptomatic, seven asymptomatic) were selected for analysis. Samples were placed in sterile bags, transported on ice to the laboratory, and stored at −80 °C until processing. Total RNA extraction was performed as described by Japelaghi et al. [15] with slight changes. First, 100 mg of grape leaves were ground in 1 mL of extraction buffer (3% CTAB, 2.1 M NaCl, 30 mM EDTA pH 8.0, 150 mM Tris-HCl pH 8.0, 3% PVP (40 kDa), 0.3% β-mercaptoethanol) using a KZ-III Tissue Homogenizer (Wuhan Servicebio Technology Co., Ltd., Wuhan, China) and 3 mm steel beads. The mixture was incubated at 60 °C for 30 min and then extracted with an equal volume of chloroform. The aqueous phase was extracted again using chloroform. Nucleic acids were collected by adding 0.1 volume of 3 M NaOAc pH 5.2, and 1 volume of isopropanol. This mixture was incubated at −80 °C for 30 min and centrifuged at 20,000 rcf for 15 min. Pellets were washed with 70% ethanol and resuspended in 450 µL of LYSIS LR buffer from a GenUP Plant RNA Kit (Biotechrabbit GmbH, Berlin, Germany, catalog number BR0701502). Further cleaning of the RNA was performed using Mini Filter DNA and Mini Filter RNA from the kit, according to the manufacturer’s instructions. RNA concentration and purity (A260/A280 and A260/A230 ratios) were quantified using a Nano-500B spectrophotometer (Hangzhou Allsheng Instruments Co., Ltd., Hangzhou, China). RNA integrity was assessed by gel electrophoresis. The extracted RNA was stored at −80 °C.

### 2.2. Library Preparation and Nanopore Sequencing

For library preparation and nanopore sequencing, the cDNA-PCR Barcoding Kit V14 (Oxford Nanopore Technologies, Oxford, UK, catalog number SQK-PCB114.24) was used with modifications (Figure S1), as described below. For cDNA synthesis, 28 ng of total RNA from each sample was mixed with 1 µL of 10 mM Oligo(dT)VN primer (5′-cttgcctgtcgctctatcttctttttttttttttttttttttttvn-3′), 1 µL of 10 mM dNTPs, and the total volume was adjusted to 11 µL with RNase-free water. A low input of 28 ng of total RNA from each sample was intentionally used, deviating from the manufacturer’s recommendation of 500 ng, to assess the workflow’s feasibility in sample-limited diagnostic scenarios. The solution was heated at 72 °C for 10 min and then quickly chilled in ice water. Next, 4 µL of Maxima H Minus 5× RT buffer (Thermo Fisher Scientific, Waltham, MA, USA, catalog number EP0752), 1 µL of RNaseOUT (Thermo Fisher Scientific, Waltham, MA, USA, catalog number 10777019), and 2 µL of SSPII (Strand Switching Primer II, SQK-PCB114.24) were added to the mixture. After preincubation at 42 °C for 2 min, 1 µL of Maxima H Minus Reverse Transcriptase (Thermo Fisher Scientific, Waltham, MA, USA, catalog number EP0752) was added. The reverse transcription reaction was incubated at 42 °C for 90 min. The RT enzyme was then inactivated by heating at 85 °C for 5 min.

For PCR amplification with barcoded primers, 5 µL of the RT reaction product was used. Further steps of library preparation, including barcoding PCR, pooling, and adapter ligation, were conducted according to the nanopore protocol for the SQK-PCB114.24 Nanopore kit. The sequence run was performed on a MinION Mk1B device using a FLO-MIN114 nanopore flow cell.

### 2.3. Bioinformatic Analysis

#### 2.3.1. Basecalling and Barcode Processing

Raw sequencing data (fast5 files) were base-called using MinKNOW software v24.11.10 (Oxford Nanopore Technologies, Oxford, UK) with a super-accurate base-calling model. Barcode demultiplexing and trimming were performed using MinKNOW to generate FASTQ files for each barcoded sample.

#### 2.3.2. Taxonomic Classification

To identify reads potentially derived from viroids, taxonomic classification was performed using Kraken2 v2.1.3 [16]. The classification was conducted using the comprehensive PlusPFP reference database (Available online: https://benlangmead.github.io/aws-indexes/k2 (accessed on 9 July 2025)), which includes archaeal, bacterial, viral, plasmid, human, plant, fungal, protozoan, and UniVec Core sequences. Reads assigned to viroid species were extracted from the original FASTQ files using the extract_kraken_reads.py script from the KrakenTools suite [17].

#### 2.3.3. Alignment and Assembly

The extracted viroid-associated reads were aligned to reference viroid genomes obtained from NCBI GenBank: NC_001920.1 for GYSVd1 and NC_001351.1 for HSVd, using BWA-MEM v0.7.17-r1188 [18] with default parameters that were suitable for nanopore reads. Alignment files (SAM/BAM format) were sorted and indexed using SAMtools [19]. A consensus genome sequence for the detected viroids was generated from the alignments using bcftools mpileup, bcftools call, and bcftools consensus [20] (Figure S2). These consensus sequences were used for subsequent secondary structure and phylogenetic analyses. The sequences were submitted to the NCBI GenBank database and assigned accession numbers PV743055 and PV743054 for GYSVd1 and HSVd, respectively.

#### 2.3.4. Secondary Structure Prediction

Predicted secondary structures for the Kazakhstani viroid sequences and their respective reference sequences were generated using the RNAstructure tool v.6.0.1 with default options [21]. Predicted secondary structures with minimum free energy were selected.

#### 2.3.5. Phylogenetic Analysis

To determine the genetic relationships between the Kazakhstani GYSVd1 and HSVd isolates, phylogenetic analyses were conducted using MEGA12 v12.0.10 [22]. Kazakhstani viroid sequences were aligned using the Muscle algorithm with a comprehensive set of reference sequences retrieved from NCBI GenBank. The best-fitting nucleotide substitution model was selected using Mega12. Phylogeny was inferred using the maximum likelihood method and the General Time Reversible (GTR) model of nucleotide substitutions [23] for GYSVd1 or the Kimura 2-parameter model [24] for HSVd. Trees with the highest log likelihood were selected.

## 3. Results and Discussion

### 3.1. RNA Quality

**Figure 1:**
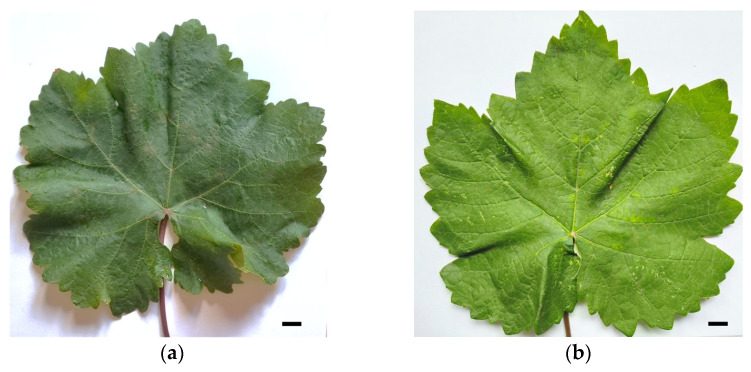
Leaves of *Vitis vinifera* cv. Alice from (**a**) healthy and (**b**) GYSVd1- and HSVd-infected plants (scale bar: 1 cm).

The samples were collected from *Vitis vinifera* L. cv. Alice grapevines. Only one sample showed signs of possible viral or viroid-like issues, such as leaf discoloration and mottling (Figure 1). The hybrid total RNA extraction method combined a modified CTAB protocol with a final spin-column purification step. This method produced RNA of sufficient quality and quantity for nanopore-sequencing-based phytopathogen detection. It consistently met the general quality control guidelines recommended by Oxford Nanopore Technologies and proved reliable for creating sequencing libraries without rRNA depletion. Spectrophotometric analysis showed that the RNA extracted from all samples of cv. Alice was high in purity and appropriate for sequencing. The purity of the RNA extracted using the CTAB method was significantly improved by a subsequent cleaning step with Mini Filter DNA and Mini Filter RNA columns (Table S1). The A260/A280 ratio ranged from 1.89 to 2.05, closely aligning with the optimal value of about 2.0 recommended by Oxford Nanopore Technologies for pure RNA. The A260/A230 ratios varied more, ranging from 1.20 to 2.15. While some samples had A260/A230 ratios below the ideal range of about 2.0 to 2.2, indicating the minor residual contamination typical in plant extractions, this did not affect successful cDNA synthesis and sequencing. Additionally, the total RNA yield from 100 mg of leaves was substantial, ranging from 10.3 to 16.8 µg, which provided enough material for library preparation. Although a formal integrity assessment, such as calculating the RNA Integrity Number (RIN), was not performed, the successful amplification and sequencing of viroid RNA directly showed its suitability for sequencing. The ability to detect viroids and other phytopathogens from these total RNA extractions highlights the importance of this workflow for phytosanitary screening, especially in situations where obtaining suitable RNA from field-grown plant tissues is difficult.

### 3.2. Viroid Detection and Identification Using the Modified Nanopore Protocol

The primary objective of this study was to assess the feasibility of a streamlined diagnostic workflow for the detection of grapevine viroids from total RNA, bypassing the need for costly and labor-intensive ribosomal RNA (rRNA) depletion or viroid enrichment procedures. To achieve this, we employed the latest generation of Oxford Nanopore Technologies (ONT) chemistry, the cDNA-PCR Barcoding Kit v14 (SQK-PCB114.24), in conjunction with an R10.4.1 flow cell (FLO-MIN114). The v14 chemistry represents a fundamental shift from previous ONT cDNA sequencing kits, such as the discontinued SQK-PCB109 kit used in earlier plant virus detection studies [25]. The standard SQK-PCB114.24 protocol is specifically engineered to ensure the sequencing of full-length polyadenylated transcripts using an adapter ligation strategy (involving a cDNA RT Adapter or CRTA) that forces reverse transcription to begin only at the true 3′ terminus of an RNA molecule. One of the key design features of this system is the active prevention of “internal priming”—the spurious initiation of cDNA synthesis from internal adenine-rich sequences within a transcript. Consequently, the standard v14 protocol is unsuitable for viroid detection, and legacy protocols based on the SQK-PCB109 kit are both technically and conceptually inapplicable. To overcome this limitation, we adapted the v14 workflow. We deliberately bypassed the kit’s standard adapter–ligation components (CRTA, RTP, and Annealing Buffer) and initiated reverse transcription by substituting a custom oligo(dT)VN priming strategy.

An initial 4 h nanopore sequencing run was conducted on a pool of eight individually barcoded libraries, each prepared from a single grapevine sample, to screen for viroid presence. This run generated a total of 912,000 reads, of which 687,000 reads passed quality filtering (minimum Q score: 8), with an N50 read length of 397 bp. A total of 95.4% of reads were successfully classified using Kraken2 (Figure S3). Kraken2 classification of the demultiplexed reads against the PlusPFP database revealed that most of the reads were derived from the plant host (*Vitis vinifera*) and common epiphytic or endophytic microorganisms. The concurrent detection of host and microbial transcripts was expected, as the oligo(dT)VN primer will anneal to the poly(A) tails of mRNAs and viral genomes in addition to the targeted internal A-rich sites within viroids. Taxonomic classification of these reads revealed that one library prepared from the cv. Alice sample was positive for both GYSVd1 and HSVd. Kraken2 classified three reads as HSVd and one read as GYSVd1. To increase the sequencing depth for this positive sample, the viroid-positive library was re-sequenced individually for 23 h, yielding 2,102,863 reads, 94.83% of which were classified (Figure S4). The number of viroid-specific reads increased significantly, with 18 and 19 reads classified as HSVd and GYSVd1, respectively. The increase in viroid-specific reads with extended sequencing time, albeit modest in absolute numbers, suggests that deeper sequencing can improve the detection likelihood or confidence of detection.

Successful detection using this non-canonical approach is the key to this study. This underscores the method’s capability, despite bypassing rRNA depletion and using an oligo(dT)VN primer. The mechanism enabling viroid cDNA synthesis via oligo(dT)VN priming was elucidated by examining the assembled viroid sequences from our samples and the read-start sites. For instance, in the HSVd sequence, reads were observed to start within A-rich regions, such as positions 33–47 (GCAAAGAAAAAACAA), including two nucleotides for the ‘VN’ anchor of the primer. Similarly, for GYSVd1, reads were initiated at A-rich sites like positions 57–71 (GCAAAGAAGAAGATA), and also on the minus strand RNA template corresponding to the T-rich region at positions TTTTTCTTTCA (297–307) of the GYSVd1 plus-strand sequence (representing an A-rich target TGAAAGAAAAA on the RNA template). The empirical success in detecting these viroids suggests that internal A-rich sites within viroid genomes can serve as initiation points for cDNA synthesis with oligo(dT)VN primers, potentially allowing their detection in standard RNA-Seq workflows.

### 3.3. Predicted Secondary Structure Analysis of Kazakhstani Viroid Isolates

**Figure 2:**
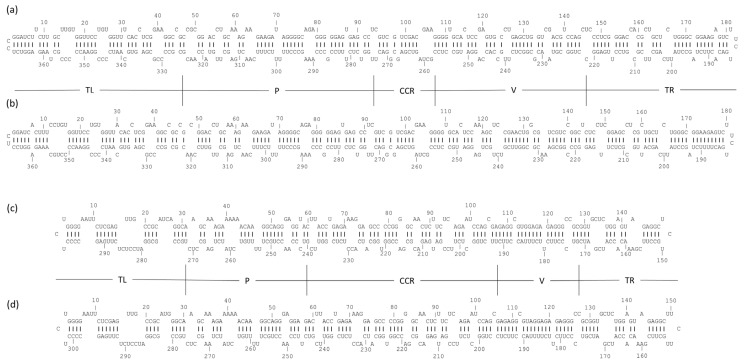
The secondary structure of Kazakhstani GYSVd1 and HSVd variants and corresponding reference isolates. (**a**) Kazakhstani GYSVd1 variant (GenBank: PV743055); (**b**) GYSVd1 reference sequence (GenBank: NC_001920.1); (**c**) Kazakhstani HSVd variant (GenBank: PV743054); (**d**) HSVd reference sequence (GenBank: NC_001351.1). Boundaries of the terminal left (TL), pathogenicity (P), central conserved region (CCR), variable (V), and terminal right (TR) regions are shown.

The alignment of the extracted viroid-specific reads to their reference genomes (NC_001351.1 for HSVd and NC_001920.1 for GYSVd1) confirmed their identity. Generated assembled sequences for the Kazakhstani isolates, named GY-KZALA-16.1 for GYSVd1 and HS-KZALA-16.2 for HSVd, were submitted to GenBank (Accession Nos. PV743055 and PV743054, respectively). To further characterize these isolates, their secondary structures were predicted computationally. This analysis is important because a viroid’s folded shape is crucial for its biological functions, such as replication, movement, and interaction with host cellular components [26]. The minimum free energy (ΔG) for GY-KZALA-16.1 was −159.0 kcal/mol, matching the reference. For HS-KZALA-16.2, the predicted ΔG was −114.0 kcal/mol, which was higher than the reference (−124.2 kcal/mol). This suggests possible structural differences in the Kazakhstani HSVd isolate. The predicted structures fit the typical quasi-rod-like branched structure of GYSVd1 (*Apscaviroidae*) and the nearly perfect rod-like structure of HSVd (*Pospiviroidae*) (Figure 2). In region 33–47 of HSVd, some nucleotides, like 33(G)-34(C) and 37(A)-39(A), are paired, but crucial adenosines, including 35(A), 36(A), and a contiguous AAAA tract (positions 40–43), are predicted to be unpaired. This indicates that these A-rich segments are in loops or bulge structures, making them accessible for primer binding. In the priming region 57–71 of GYSVd1, several adenosine residues (A59, A60, A63, and A64) and a uracil (U70) are unpaired and mixed within an otherwise base-paired stem. The GYSVd1 sequence also has a minus-strand target that corresponds to plus-strand nucleotides 297–307, where several nucleotides (U299, U300, C306, and A307) are predicted to be unpaired. These unpaired bases in small internal loops or bulges, along with the heat denaturation step, support the idea that internal A-rich sites become exposed enough for oligo(dT)VN priming.

### 3.4. Phylogenetic Analysis of Kazakhstani Viroid Isolates

To elucidate the genetic relationships and potential origins of the viroids detected in *Vitis vinifera* cv. Alice, phylogenetic analyses were conducted using the maximum likelihood method, with the topological stability of key clades assessed via bootstrap analysis (1000 replicates). The results revealed distinct European introductions and a complex epidemiological history in Kazakhstan.

**Figure 3:**
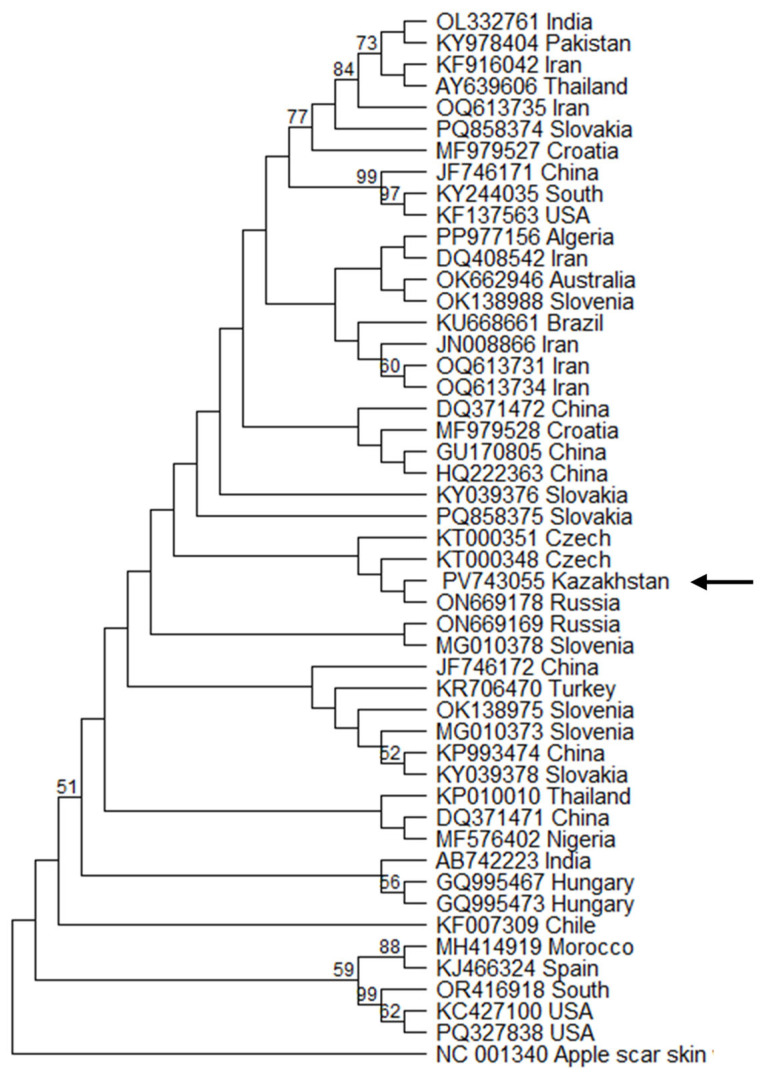
The maximum likelihood tree of GYSVd1 sequence variants from Genebank. Sequence variant determined in this work indicated by arrow. Variants named by accession number followed by country of origin.

For GYSVd-1 the phylogeny was inferred using the General Time Reversible (GTR) model of nucleotide substitution. The tree with the highest log likelihood (−1715.55), constructed from a final dataset of 361 positions from 49 sequences, positioned the Kazakh isolate GY-KZALA-16.1 in a subclade with isolates from the Czech Republic (KT000348) and Russia (ON669178) (Figure 3). While the bootstrap support for this specific clade is low, its placement within a broader group of predominantly European isolates suggests a plausible introduction of this viroid into Kazakhstan via the movement of contaminated grapevine material from Europe. However, this interpretation must be made with caution given the lack of strong statistical support for the deeper nodes of the tree. This finding is consistent with the high prevalence of GYSVd-1 in these regions [27].

**Figure 4:**
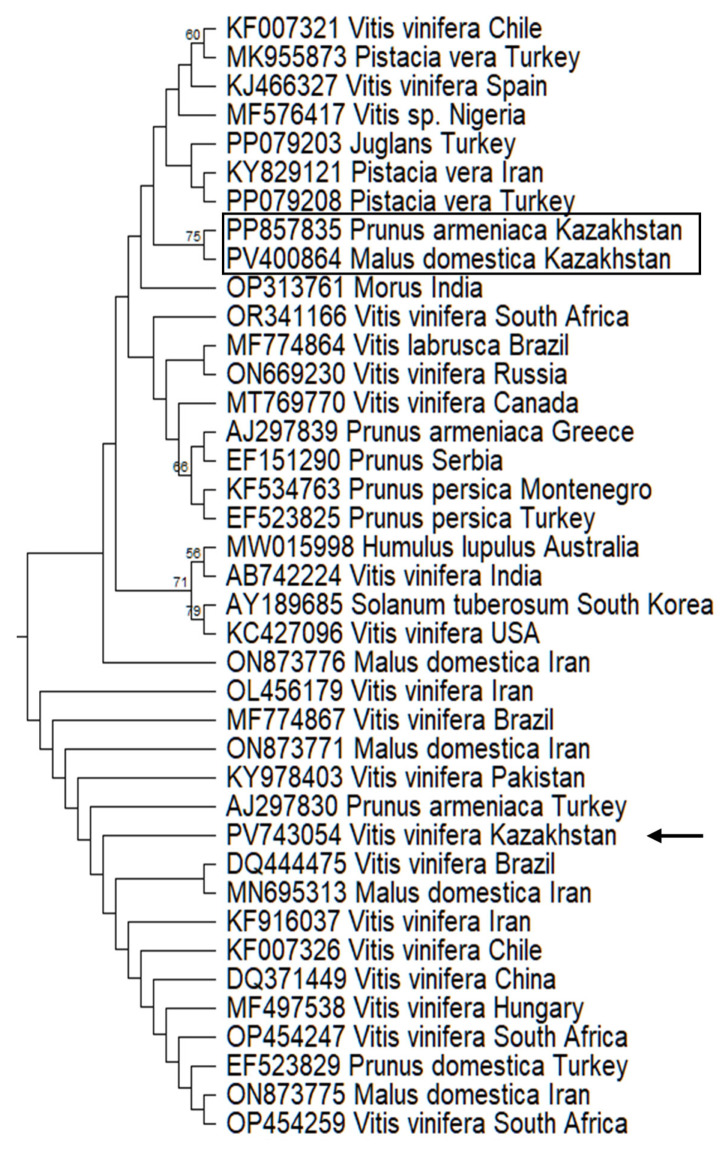
Part of the maximum likelihood tree (Figure S5) of HSVd sequence variants from Genebank. Sequence variant determined in this work indicated by arrow. Kazakhstani variants of HSVd published by other authors indicated by rectangle. Variants of viroid named by accession number followed by host and country of origin.

Phylogenetic analysis of HSVd was conducted on a dataset of 134 nucleotide sequences using the Kimura 2-parameter model. The final analysis, based on 289 positions, produced a tree with the highest log likelihood (−2841.15) and placed the Kazakhstani grapevine isolate HS-KZALA-16.2 (PV743054) into a large, well-supported clade with a broad, intercontinental distribution (Figure 4). This clade is comprised of highly similar HSVd sequences isolated from mostly *V. vinifera*, but also from Malus domestica and Prunus species. Our isolate clusters with strains from Asia, including Iran (ON873771), Pakistan (KY978403), and China (DQ371449); South America, including Brazil (MF774867) and Chile (KF007326); Africa (South Africa, OP454259); and Europe, including Hungary (MF497538) and Turkey (AJ297830). This indicates that the Kazakhstani isolate belongs to a very common and globally widespread HSVd lineage rather than a geographically restricted one.

The epidemiological scenario becomes more complex when considering other HSVd isolates previously identified in Kazakhstan in different hosts. These isolates from apricot (*Prunus armeniaca*) and apple (*Malus domestica*) were located in a completely different phylogenetic clade. The significant genetic distance between the grapevine-infecting and fruit-tree-infecting lineages provides conclusive evidence for at least two separate, independent introductions of HSVd into Kazakhstan. This phenomenon of co-circulating host-associated viroid lineages has been observed in other regions, such as New Zealand and Iran [28,29]. For example, a study in New Zealand identified two genetically distinct sequence types of HSVd coexisting within a single vineyard, leading the authors to conclude that this finding suggested “at least two separate introductions of HSVd into the vineyard”.

Specific European phylogenetic links can be explained by the genetic heritage of the host cultivar. ‘Alice’ is a complex hybrid whose ancestry provides direct potential pathways for the vertical transmission of these viroids through the breeding stock. Its lineage includes the Russian variety ‘Vostorg’ and the Moldovan ‘Frumoasa Albă’, which directly corroborates the connection of the GYSVd-1 isolate to Russia and Eastern Europe [30]. The ancestry of ‘Alice’ includes the other European varieties ‘Pierrelle’ and ‘Perle Noire’, providing a strong direct ancestral link that explains the presence of a Hungarian-type HSVd strain.

This suggests that the viroids were likely introduced into the region via co-transport with the genetic material of grapevine ancestors. Both viroids are transmitted by seeds, in addition to vegetative propagation material [6]. While this vertical transmission through contaminated propagation material or infected seeds is the most compelling hypothesis, it is important to interpret viroid phylogenetics with caution because of the possibility of convergent evolution, where identical mutations can arise independently to preserve the viroid’s critical secondary structure [27]. Therefore, although a direct epidemiological link is the most compelling hypothesis, high sequence identity is not absolute proof. Nevertheless, these findings reveal systemic vulnerabilities and highlight the latent threat posed by asymptomatic infections, which renders visual inspection for phytosanitary control ineffective. The exceptionally broad host range of HSVd further magnifies this risk, demonstrating a cross-commodity threat to both viticulture and horticulture sectors. These results underscore the urgent need for a robust national phytosanitary certification program based on sensitive molecular diagnostics to protect Kazakhstan’s agricultural industries from the economic impact of these and other latent pathogens.

## 4. Conclusions

This study successfully demonstrated that nanopore sequencing of total RNA, without prior rRNA depletion and utilizing an oligo(dT)VN primer, is a viable method for detecting Grapevine yellow speckle viroid 1 and Hop stunt viroid in *Vitis vinifera*. Full-length sequences of GYSVd1 and HSVd from grapevines in Kazakhstan were acquired for first time. Predicted secondary structures suggest accessibility of internal A-rich priming sites. Phylogenetic analysis indicated a close relationship with Eurasian strains. These findings provide valuable initial data on the presence of viroids and their genetic context in Grapevines in Kazakhstan, highlighting the utility of this simplified sequencing approach for regional phytosanitary efforts. This workflow is also applicable to the detection of polyadenylated or other RNA viruses, further broadening its utility as a general tool for phytosanitary screening. However, to fully validate this workflow for diagnostic use, future research should focus on determining the limit of detection and the sequencing depth required to match the sensitivity of conventional methods like RT-PCR and RT-qPCR. Such validation would ideally involve the use of infectious clones to analyze samples with varying viroid loads, representing different stages of infection.

## Data Availability

Nucleotide sequences obtained in this study were deposited in the NCBI GenBank under accession numbers PV743055 and PV743054.

## Supplementary Materials

The following supporting information can be downloaded at: https://www.mdpi.com/article/10.3390/pathogens14080782/s1, Figure S1: Schematic comparison of the standard and modified sequencing workflows; Figure S2: Bioinformatics pipeline for viroid consensus genome assembly; Figure S3: Taxonomic classification of reads from the initial 4 h nanopore screening run; Figure S4: Taxonomic classification of reads from 23 h nanopore screening run; Figure S5: The neighbor-joining tree of HSVd sequence variants from Genebank. Sequence variant determined in this work indicated by arrow. Kazakhstani variants of HSVd published by other authors indicated by rectangle. Variants of viroid named by accession number followed by host and country of origin; Table S1: Total RNA quantity and quality measured by Nano-500B spectrophotometer after extraction using CTAB and further cleaning using Mini Filter DNA and Mini Filter RNA.

## Abbreviations

The following abbreviations are used in this manuscript:

GYSVd1	Grapevine yellow speckle viroid 1
HSVd	Hop stunt viroid
rRNA	Ribosomal RNA
RT	Reverse transcription
CRTA	cDNA RT adapter
RTP	RT primer
HTS	High-throughput sequencing
ONT	Oxford Nanopore Technologies
CTAB	Cetyltrimethylammonium bromide
