# Supplementary material for: Nanopore Workflow for Grapevine Viroid Surveillance in Kazakhstan: Bypassing rRNA Depletion Through Non-Canonical Priming
Source: Pathogens. 2025 Aug 6;14(8):782. doi: 10.3390/pathogens14080782 (PMC12388932; doi:10.3390/pathogens14080782)
Supplement: Supplementary file 1 [file pathogens-14-00782-s001.zip › Figure S2.pdf]

```
#!/bin/bash
if [ "$#" -lt 3 ]; then
        echo "not enough arguments. first reference then fastq and last reads"
        exit 1
fi
REF_FASTA=$1
FASTQ_FILE=$2
THRESHOLD=$3
echo "Indexing reference FASTA file..."
echo "Reference FASTA: $REF_FASTA"
samtools faidx $REF_FASTA
echo "Indexing reference for BWA..."
bwa index $REF_FASTA
echo "Aligning reads..."
bwa mem -x ont2d $REF_FASTA $FASTQ_FILE > alignment.sam
echo "Converting and sorting alignment..."
samtools view -b alignment.sam > alignment.bam
samtools sort alignment.bam -o sortedAlignment.bam
samtools index sortedAlignment.bam
echo "Calculating coverage..."
samtools depth -a sortedAlignment.bam > coverage.txt
echo "Creating BED file for low coverage regions..."
awk -v thresh=$THRESHOLD '$3 < thresh {print $1 "\t" $2-1 "\t" $2}' coverage.txt
> low_coverage.bed
echo "Merging adjacent low coverage positions..."
bedtools merge -i low_coverage.bed > merged_low_coverage.bed
echo "Calling variants..."
bcftools mpileup -f $REF_FASTA sortedAlignment.bam | bcftools call -c --ploidy 1
-o variants.vcf
echo "Compressing and indexing VCF file..."
bgzip variants.vcf
tabix -p vcf variants.vcf.gz
echo "Generating consensus sequence with masking..."
bcftools consensus -f $REF_FASTA -m merged_low_coverage.bed variants.vcf.gz >
consensus_masked.fasta
echo "Done!"
```
