# Supplementary material for: Nanopore Workflow for Grapevine Viroid Surveillance in Kazakhstan: Bypassing rRNA Depletion Through Non-Canonical Priming
Source: Pathogens. 2025 Aug 6;14(8):782. doi: 10.3390/pathogens14080782 (PMC12388932; doi:10.3390/pathogens14080782)
Supplement: Supplementary file 1 [file pathogens-14-00782-s001.zip › Figure S3.pdf]

4.64	5380	5380	U	0	unclassified
95.36	110572	1	R	1	root
95.35	111430	497	1	131567	cellular organisms
94.79	110770	302		2759	Eukaryota
93.68	109478	4	K	33090	Viridiplantae
93.68	109474	0	P	35493	Streptophyta
93.68	109474	0	P1	131221	Streptophytina
93.68	109474	102	P2	3193	Embryophyta
93.59	109372	0	P3	58023	Tracheophyta
93.59	109372	0	P4	78536	Euphyllophyta
93.59	109372	186	P5	58024	Spermatophyta
93.40	109146	1425	C	3398	Magnoliopsida
92.11	107637	5908	C1	1437183	Mesangiospermae
81.62	95380	0	C2	71240	eudicotyledons
81.62	95380	0	C3	91827	Gunneridae
81.62	95380	4066	C4	1437201	Pentapetalae
73.48	85868	2268	C5	71275	rosids
60.03	70150	0	C6	91834	rosids incertae sedis
60.03	70150	0	O	403667	Vitales
60.03	70150	0	F	3602	Vitaceae
60.03	70150	0	F1	2304100	Viteae
60.03	70150	26765	G	3603	Vitis
21.63	25275	25275	S	96939	Vitis riparia
15.50	18110	18110	S	29760	Vitis vinifera
7.60	8881	1163	C6	91835	fabids
1.78	2075	0	O	72025	Fabales
1.78	2075	0	F	3803	Fabaceae
1.78	2075	0	F1	3814	Papilionoideae
1.78	2075	37	F2	2231393	50 kb inversion clade
0.86	1004	0	F3	2231387	dalbergioids sensu lato
0.86	1004	0	F4	163725	Dalbergieae
0.86	1004	0	F5	2231390	Pterocarpus clade
0.86	1004	148	G	3817	Arachis
0.60	707	707	S	217475	Arachis stenosperma
0.06	71	71	S	3818	Arachis hypogaea
0.06	67	67	S	130453	Arachis duranensis
0.01	11	11	S	130454	Arachis ipaensis
0.78	910	6	F3	2231382	NPAAA clade

0.44	520	2	F4	2233838	Hologalegina
0.38	443	51	F5	2233839	IRL clade
0.14	159	0	F6	163722	Cicereae
0.14	159	0	G	3826	Cicer
0.14	159	159	S	3827	Cicer arietinum
0.11	128	0	F6	163743	Fabeae
0.10	113	0	G	3887	Pisum
0.10	113	113	S	3888	Pisum sativum
0.01	15	0	G	3904	Vicia
0.01	15	15	S	3911	Vicia villosa
0.09	105	0	F6	163742	Trifolieae
0.08	93	0	G	3898	Trifolium
0.08	93	93	S	57577	Trifolium pratense
0.01	12	0	G	3877	Medicago
0.01	12	12	S	3880	Medicago truncatula
0.06	75	0	F5	2233857	robinioid clade
0.06	75	0	F6	163747	Loteae
0.06	75	0	G	3867	Lotus
0.06	75	75	S	34305	Lotus japonicus
0.33	384	0	F4	2233855	indigoferoid/millettioid clade
0.33	384	13	F5	163735	Phaseoleae
0.19	227	0	G	3913	Vigna
0.10	119	119	S	3917	Vigna unguiculata
0.08	97	0	S	157791	Vigna radiata
0.08	97	97	S1	3916	Vigna radiata var. radiata
0.01	11	11	S	3914	Vigna angularis
0.07	83	0	G	3846	Glycine
0.07	83	60	G1	1462606	Glycine subgen. Soja
0.02	20	20	S	3847	Glycine max
0.00	3	3	S	3848	Glycine soja
0.03	33	0	G	3820	Cajanus
0.03	33	33	S	3821	Cajanus cajan
0.02	28	0	G	3883	Phaseolus
0.02	28	28	S	3885	Phaseolus vulgaris
0.11	124	0	F3	2231384	genistoids sensu lato
0.11	124	0	F4	2231385	core genistoids

0.11	124	0	F5	163729	Genisteae
0.11	124	0	G	3869	Lupinus
0.11	124	124	S	3871	Lupinus angustifolius
1.33	1550	20	O	3744	Rosales
0.86	1010	159	F	3745	Rosaceae
0.40	464	9	F1	171637	Amygdaloideae
0.22	259	0	F2	721813	Maleae
0.22	259	117	G	3749	Malus
0.08	90	90	S	3752	Malus sylvestris
0.04	52	52	S	3750	Malus domestica
0.17	196	0	F2	721805	Amygdaleae
0.17	196	43	G	3754	Prunus
0.06	70	70	S	3760	Prunus persica
0.04	48	48	S	102107	Prunus mume
0.03	35	35	S	3755	Prunus dulcis
0.33	387	29	F1	171638	Rosoideae
0.18	213	0	F2	1176516	Rosoideae incertae sedis
0.18	213	52	G	3764	Rosa
0.08	94	94	S	74645	Rosa rugosa
0.06	67	67	S	74649	Rosa chinensis
0.12	145	4	F2	721789	Potentilleae
0.08	90	0	F3	1184124	Fragariinae
0.08	90	0	G	3746	Fragaria
0.08	90	0	S	57918	Fragaria vesca
0.08	90	90	S1	101020	Fragaria vesca subsp. vesca
0.04	51	0	F3	1184125	Potentilleae incertae sedis
0.04	51	0	G	23204	Potentilla
0.04	51	51	S	57926	Argentina anserina
0.24	283	6	F	3481	Cannabaceae
0.13	152	0	G	3484	Humulus
0.13	152	152	S	3486	Humulus lupulus
0.11	125	0	G	3482	Cannabis
0.11	125	125	S	3483	Cannabis sativa
0.20	237	0	F	3608	Rhamnaceae
0.20	237	0	F1	325284	Paliureae
0.20	237	0	G	72171	Ziziphus
0.20	237	237	S	326968	Ziziphus jujuba

1.24	1445	0	O	71239	Cucurbitales
1.24	1445	27	F	3650	Cucurbitaceae
1.07	1247	21	F1	1003877	Benincaseae
0.66	770	0	G	3655	Cucumis
0.63	736	736	S	3656	Cucumis melo
0.03	34	34	S	3659	Cucumis sativus
0.39	456	0	G	102210	Benincasa
0.39	456	456	S	102211	Benincasa hispida
0.15	171	0	F1	1003878	Cucurbiteae
0.15	171	0	G	3660	Cucurbita
0.15	171	0	S	3663	Cucurbita pepo
0.15	171	171	S1	3664	Cucurbita pepo subsp. pepo
1.22	1430	44	O	3502	Fagales
0.70	817	54	F	16714	Juglandaceae
0.61	714	9	G	16718	Juglans
0.56	650	650	S	51240	Juglans regia
0.05	55	55	S	2249226	Juglans microcarpa x Juglans regia
0.04	49	0	G	13402	Carya
0.04	49	49	S	32201	Carya illinoinensis
0.35	408	0	F	3503	Fagaceae
0.35	408	29	G	3511	Quercus
0.24	286	286	S	38942	Quercus robur
0.08	93	93	S	97700	Quercus lobata
0.14	161	0	F	3514	Betulaceae
0.10	115	0	G	3515	Alnus
0.10	115	115	S	3517	Alnus glutinosa
0.04	46	0	G	13450	Corylus
0.04	46	46	S	13451	Corylus avellana
0.83	971	9	O	3646	Malpighiales
0.65	754	9	F	3977	Euphorbiaceae
0.38	444	0	F1	235629	Acalyphoideae
0.38	444	2	F2	235880	Acalypheae
0.21	249	0	G	3987	Ricinus
0.21	249	249	S	3988	Ricinus communis
0.17	193	0	G	3984	Mercurialis
0.17	193	193	S	3986	Mercurialis annua
0.14	164	1	F1	235631	Crotonoideae

0.08	89	0	F2	235883	Manihoteae
0.08	89	0	G	3982	Manihot
0.08	89	89	S	3983	Manihot esculenta
0.06	74	0	F2	235882	Micrandreae
0.06	74	0	G	3980	Hevea
0.06	74	74	S	3981	Hevea brasiliensis
0.12	137	0	F1	235633	Euphorbioideae
0.12	137	0	F2	235895	Euphorbieae
0.12	137	0	G	3990	Euphorbia
0.12	137	0	G1	1334278	Euphorbia subgen. Esula
0.12	137	0	G2	1334446	Euphorbia sect. Lathyris
0.12	137	137	S	212925	Euphorbia lathyris
0.18	208	0	F	3688	Salicaceae
0.18	208	0	F1	238069	Saliceae
0.18	208	14	G	3689	Populus
0.11	129	129	S	3691	Populus nigra
0.06	65	65	S	3694	Populus trichocarpa
0.21	247	0	O	233875	Celastrales
0.21	247	0	F	4305	Celastraceae
0.21	247	0	G	123484	Tripterygium
0.21	247	247	S	458696	Tripterygium wilfordii
3.91	4569	255	C6	91836	malvids
1.90	2225	0	O	41938	Malvales
1.90	2225	2	F	3629	Malvaceae
1.90	2217	0	F1	214907	Malvoideae
1.90	2217	309	G	3633	Gossypium
0.82	955	955	S	29729	Gossypium arboreum
0.47	546	546	S	29730	Gossypium raimondii
0.35	407	407	S	3635	Gossypium hirsutum
0.01	6	0	F1	214909	Byttnerioideae
0.01	6	0	G	3640	Theobroma
0.01	6	6	S	3641	Theobroma cacao
1.34	1563	0	O	3699	Brassicales
1.34	1563	2	F	3700	Brassicaceae
1.31	1526	45	F1	981071	Brassiceae
1.21	1412	251	G	3705	Brassica
0.87	1016	1016	S	3708	Brassica napus
0.10	112	112	S	3711	Brassica rapa

0.03	33	0	S	3712	Brassica oleracea
0.03	33	33	S1	109376	Brassica oleracea var. oleracea
0.06	69	0	G	3725	Raphanus
0.06	69	69	S	3726	Raphanus sativus
0.03	35	0	F1	980083	Camelineae
0.03	33	0	G	71323	Camelina
0.03	33	33	S	90675	Camelina sativa
0.00	2	0	G	3701	Arabidopsis
0.00	2	2	S	3702	Arabidopsis thaliana
0.35	405	1	O	41944	Myrtales
0.29	342	0	F	3931	Myrtaceae
0.29	342	30	F1	1699513	Myrtoideae
0.18	210	0	F2	1699524	Eucalypteae
0.18	210	0	G	3932	Eucalyptus
0.18	210	210	S	71139	Eucalyptus grandis
0.09	102	0	F2	1699523	Myrteae
0.09	102	0	F3	1705102	Australasian group
0.09	102	0	G	178132	Rhodamnia
0.09	102	102	S	178133	Rhodamnia argentea
0.05	62	0	F	3928	Lythraceae
0.05	62	0	G	22662	Punica
0.05	62	62	S	22663	Punica granatum
0.10	121	0	O	41937	Sapindales
0.09	107	0	F	4011	Anacardiaceae
0.09	107	0	G	23461	Mangifera
0.09	107	107	S	29780	Mangifera indica
0.01	14	0	F	23513	Rutaceae
0.01	14	0	F1	1728959	Aurantioideae
0.01	14	0	G	2706	Citrus
0.01	14	14	S	2711	Citrus sinensis
3.81	4456	386	C5	71274	asterids
2.37	2765	106	C6	91888	lamiids
1.88	2192	9	O	4069	Solanales
1.81	2116	108	F	4070	Solanaceae
1.29	1512	123	F1	424551	Solanoideae
0.55	640	0	F2	424574	Solaneae
0.55	640	30	G	4107	Solanum

0.24	284	121	G1	49274	Solanum subgen. Lycopersicon
0.09	103	103	S	4081	Solanum lycopersicum
0.05	60	60	S	28526	Solanum pennellii
0.15	178	178	S	172797	Solanum stenotomum
0.13	148	148	S	45834	Solanum dulcamara
0.41	481	0	F2	424564	Capsiceae
0.41	481	0	G	4071	Capsicum
0.41	481	481	S	4072	Capsicum annuum
0.23	268	0	F2	424569	Lycieae
0.23	268	57	G	24646	Lycium
0.11	126	126	S	112874	Lycium ferocissimum
0.07	85	85	S	112863	Lycium barbarum
0.42	496	0	F1	424554	Nicotianoideae
0.42	496	0	F2	424562	Nicotianeae
0.42	496	0	G	4085	Nicotiana
0.42	496	496	S	49451	Nicotiana attenuata
0.06	67	0	F	4118	Convolvulaceae
0.06	67	0	F1	267213	Ipomoeeae
0.06	67	0	G	4119	Ipomoea
0.06	67	67	S	35885	Ipomoea triloba
0.28	330	4	O	4143	Lamiales
0.19	217	0	F	4144	Oleaceae
0.19	217	0	F1	426106	Oleeae
0.19	217	0	G	4145	Olea
0.19	217	0	S	4146	Olea europaea
0.19	217	0	S1	158383	Olea europaea subsp. europaea
0.19	217	217	S2	158386	Olea europaea var. sylvestris
0.06	71	0	F	4136	Lamiaceae
0.06	71	0	F1	216706	Nepetoideae
0.06	71	0	F2	216718	Mentheae
0.06	71	0	F3	2836339	Salviinae
0.06	71	2	G	21880	Salvia
0.03	40	0	G1	2026555	Salvia subgen. Calosphace
0.03	40	0	G2	2026556	core Calosphace
0.03	40	40	S	180675	Salvia splendens
0.02	29	0	G1	2291027	Salvia incertae sedis
0.01	15	15	S	49212	Salvia hispanica

0.01	14	14	S	226208	Salvia miltiorrhiza
0.03	38	0	F	4180	Pedaliaceae
0.03	38	0	G	4181	Sesamum
0.03	38	38	S	4182	Sesamum indicum
0.12	137	0	O	4055	Gentianales
0.12	137	0	F	24966	Rubiaceae
0.12	137	0	F1	169618	Ixoroideae
0.12	137	0	F2	1968429	Gardenieae complex
0.12	137	0	F3	1968428	Bertiereae - Coffeeae clade
0.12	137	0	F4	169640	Coffeeae
0.12	137	58	G	13442	Coffea
0.05	62	62	S	13443	Coffea arabica
0.01	17	17	S	49369	Coffea eugenioides
0.85	990	6	C6	91882	campanulids
0.78	913	0	O	4209	Asterales
0.78	913	54	F	4210	Asteraceae
0.37	428	0	F1	219103	Carduoideae
0.37	428	0	F2	102818	Cardueae
0.37	428	0	F3	742010	Carduinae
0.37	428	0	G	4264	Cynara
0.37	428	0	S	4265	Cynara cardunculus
0.37	428	0	S1	309979	Cynara cardunculus subsp. carduncullus
0.37	428	428	S2	59895	Cynara cardunculus var. scolymus
0.19	223	0	F1	219120	Cichorioideae
0.19	223	0	F2	219121	Cichorieae
0.19	223	0	F3	745062	Lactucinae
0.19	223	0	G	4235	Lactuca
0.19	223	223	S	4236	Lactuca sativa
0.18	208	1	F1	102804	Asteroideae
0.09	110	0	F2	911341	Heliantheae alliance
0.09	110	0	F3	102814	Heliantheae
0.09	110	0	G	4231	Helianthus
0.09	110	110	S	4232	Helianthus annuus
0.08	97	0	F2	102809	Astereae
0.08	97	0	F3	877976	North American clade
0.08	97	0	F4	2841728	Conyzinae

0.08	97	0	G	41574	Erigeron
0.08	97	97	S	72917	Erigeron canadensis
0.06	71	0	O	4036	Apiales
0.06	71	0	O1	364270	Apiineae
0.06	71	0	F	4037	Apiaceae
0.06	71	0	F1	241778	Apioideae
0.06	71	0	F2	241789	Scandiceae
0.06	71	0	F3	241799	Daucinae
0.06	71	0	G	4038	Daucus
0.06	71	0	G1	1873447	Daucus sect. Daucus
0.06	71	0	S	4039	Daucus carota
0.06	71	71	S1	79200	Daucus carota subsp. sativus
0.27	315	19	O	41945	Ericales
0.11	129	0	F	3623	Actinidiaceae
0.11	129	0	G	3624	Actinidia
0.11	129	129	S	165200	Actinidia eriantha
0.05	62	0	F	19955	Ebenaceae
0.05	62	0	G	13492	Diospyros
0.05	62	62	S	55363	Diospyros lotus
0.05	58	0	F	25692	Balsaminaceae
0.05	58	0	G	35939	Impatiens
0.05	58	58	S	253017	Impatiens glandulifera
0.04	47	0	F	4345	Ericaceae
0.04	47	0	F1	217035	Ericoideae
0.04	47	0	F2	217046	Rhodoreae
0.04	47	0	G	4346	Rhododendron
0.04	47	47	S	182163	Rhododendron vialii
0.61	716	0	O	41947	Santalales
0.61	716	0	F	1003242	Ximeniaceae
0.61	716	0	G	397391	Malania
0.61	716	716	S	397392	Malania oleifera
0.23	274	4	O	3524	Caryophyllales
0.17	204	1	F	1804623	Chenopodiaceae
0.11	129	0	F1	1307796	Chenopodioideae
0.11	129	0	F2	1307775	Anserineae
0.11	129	0	G	3561	Spinacia
0.11	129	129	S	3562	Spinacia oleracea

0.06	74	0	F1	1804621	Betoideae
0.06	74	0	G	3554	Beta
0.06	74	0	S	161934	Beta vulgaris
0.06	74	74	S1	3555	Beta vulgaris subsp. vulgaris
0.06	66	0	F	3563	Amaranthaceae
0.06	66	0	G	3564	Amaranthus
0.06	66	66	S	29722	Amaranthus tricolor
3.14	3664	0	C2	4447	Liliopsida
3.14	3664	14	C3	1437197	Petrosaviidae
2.95	3452	36	C4	4734	commelinids
2.65	3100	3	O	38820	Poales
2.64	3087	78	F	4479	Poaceae
2.01	2349	6	F1	359160	BOP clade
1.98	2318	12	F2	147368	Pooideae
1.55	1814	0	F3	1648038	Triticodae
1.55	1814	55	F4	147389	Triticeae
0.88	1029	103	F5	1648030	Triticinae
0.75	877	304	G	4564	Triticum
0.31	358	358	S	85692	Triticum dicoccoides
0.12	145	145	S	4565	Triticum aestivum
0.06	70	70	S	4572	Triticum urartu
0.04	49	0	G	4480	Aegilops
0.04	49	0	S	37682	Aegilops tauschii
0.04	49	49	S1	200361	Aegilops tauschii subsp. strangulata
0.62	730	0	F5	1648017	Hordeinae
0.62	730	0	G	4512	Hordeum
0.62	730	0	S	4513	Hordeum vulgare
0.62	730	730	S1	112509	Hordeum vulgare subsp. vulgare
0.41	482	0	F3	1648037	Poodae
0.41	482	0	F4	147387	Poeae
0.41	482	0	F5	1652081	Poeae Chloroplast Group 2 (Poeae type)
0.41	482	0	F6	2948571	Loliodinae
0.41	482	0	F7	640630	Loliinae
0.41	482	3	G	4520	Lolium
0.36	416	416	S	89674	Lolium rigidum

0.05	63	63	S	4522	Lolium perenne
0.01	10	0	F3	2822797	Stipodae
0.01	10	0	F4	147385	Brachypodieae
0.01	10	0	G	15367	Brachypodium
0.01	10	10	S	15368	Brachypodium distachyon
0.02	25	0	F2	147367	Oryzoideae
0.02	25	0	F3	147380	Oryzeae
0.02	25	0	F4	1648021	Oryzinae
0.02	25	6	G	4527	Oryza
0.01	13	0	S	4530	Oryza sativa
0.01	13	13	S1	39947	Oryza sativa Japonica Group
0.00	4	4	S	4533	Oryza brachyantha
0.00	2	2	S	4538	Oryza glaberrima
0.56	660	2	F1	147370	PACMAD clade
0.51	596	27	F2	147369	Panicoideae
0.35	413	0	F3	1648033	Andropogonodae
0.35	413	0	F4	147429	Andropogoneae
0.34	393	0	F5	1648029	Tripsacinae
0.34	393	0	G	4575	Zea
0.34	393	393	S	4577	Zea mays
0.01	13	0	F5	1648028	Sorghinae
0.01	13	0	G	4557	Sorghum
0.01	13	13	S	4558	Sorghum bicolor
0.01	7	0	F5	1648026	Saccharinae
0.01	7	0	G	62336	Miscanthus
0.01	7	7	S	154761	Miscanthus floridulus
0.13	156	0	F3	1648036	Panicodae
0.13	156	0	F4	147428	Paniceae
0.12	137	0	F5	1293365	Panicinae
0.12	137	2	G	4539	Panicum
0.08	97	0	G1	2100772	Panicum sect. Panicum
0.08	97	97	S	206008	Panicum hallii
0.03	38	0	G1	2100771	Panicum sect. Hiantes
0.03	38	38	S	38727	Panicum virgatum
0.02	19	0	F5	1293361	Cenchrinae
0.02	19	0	G	4554	Setaria
0.01	15	15	S	4555	Setaria italica
0.00	4	4	S	4556	Setaria viridis

0.05	62	0	F2	156631	Arundinoideae
0.05	62	0	F3	1648043	Molinieae
0.05	62	0	F4	2949676	Molininae
0.05	62	0	G	15745	Phragmites
0.05	62	62	S	29695	Phragmites australis
0.01	10	0	F	4613	Bromeliaceae
0.01	10	0	F1	1909378	Bromelioideae
0.01	10	0	G	4614	Ananas
0.01	10	10	S	4615	Ananas comosus
0.18	206	9	O	4618	Zingiberales
0.13	154	0	F	4637	Musaceae
0.13	154	0	G	4640	Musa
0.13	154	0	S	4641	Musa acuminata
0.13	154	154	S1	214697	Musa acuminata AAA Group
0.04	43	0	F	4642	Zingiberaceae
0.04	43	0	G	4650	Zingiber
0.04	43	43	S	94328	Zingiber officinale
0.09	110	0	O	40551	Arecales
0.09	110	3	F	4710	Arecaceae
0.06	72	0	F1	169697	Arecoideae
0.06	72	0	F2	169705	Cocoseae
0.06	72	0	F3	169729	Elaeidinae
0.06	72	0	G	51952	Elaeis
0.06	72	72	S	51953	Elaeis guineensis
0.03	35	0	F1	169700	Coryphoideae
0.03	35	0	F2	169748	Phoeniceae
0.03	35	0	G	4719	Phoenix
0.03	35	35	S	42345	Phoenix dactylifera
0.15	177	0	O	40548	Dioscoreales
0.15	177	0	F	4671	Dioscoreaceae
0.15	177	0	G	4672	Dioscorea
0.15	177	0	S	29710	Dioscorea cayenensis
0.15	177	177	S1	55577	Dioscorea cayenensis subsp. rotundata
0.02	21	0	O	73496	Asparagales
0.02	21	0	F	40552	Asparagaceae
0.02	21	0	F1	703533	Asparagoideae
0.02	21	0	G	4685	Asparagus
0.02	21	21	S	4686	Asparagus officinalis

1.51	1768	0	O	41768	Ranunculales
1.51	1768	0	F	3465	Papaveraceae
1.51	1768	0	F1	1462614	Papaveroideae
1.51	1768	0	G	3468	Papaver
1.51	1768	1768	S	3469	Papaver somniferum
0.40	465	0	C2	232347	Magnoliidae
0.40	465	0	O	3400	Magnoliales
0.40	465	0	F	3401	Magnoliaceae
0.40	465	0	G	3402	Magnolia
0.40	465	465	S	86752	Magnolia sinica
0.39	452	0	O	232378	Proteales
0.39	452	7	F	4328	Proteaceae
0.26	305	0	G	54954	Telopea
0.26	305	305	S	54955	Telopea speciosissima
0.12	140	0	G	4329	Macadamia
0.12	140	140	S	60698	Macadamia integrifolia
0.07	84	0	O	261007	Nymphaeales
0.07	84	0	F	4410	Nymphaeaceae
0.07	84	0	G	4418	Nymphaea
0.07	84	84	S	210225	Nymphaea colorata
0.03	40	0	P6	1437180	Acrogymnospermae
0.03	40	0	C	58019	Pinopsida
0.03	40	0	C1	3313	Pinidae
0.03	40	0	C2	2821351	Conifers II
0.03	40	0	O	1446379	Cupressales
0.03	40	0	F	3367	Cupressaceae
0.03	40	0	G	3368	Cryptomeria
0.03	40	40	S	3369	Cryptomeria japonica
0.84	107	0	D1	33154	Opisthokonta
0.09	107	0	K	4751	Fungi
0.09	105	7	K1	451864	Dikarya
0.07	80	0	P	4890	Ascomycota
0.07	79	3	P1	716545	saccharomyceta
0.05	64	0	P2	147538	Pezizomycotina
0.05	64	1	P3	716546	leotiomyceta
0.02	27	0	P4	715989	sordariomyceta
0.02	23	0	C	147550	Sordariomycetes
0.02	19	1	C1	222543	Hypocreomycetidae

0.01	17	3	O	5125	Hypocreales
0.01	12	0	F	110618	Nectriaceae
0.01	12	0	G	5506	Fusarium
0.01	6	1	G1	232080	Fusarium solani species complex
0.00	5	5	S	1328300	Fusarium keratoplasticum
0.00	4	0	G1	569360	Fusarium sambucinum species complex
0.00	3	3	S	36050	Fusarium poae
0.00	1	0	S	5518	Fusarium graminearum
0.00	1	1	S1	229533	Fusarium graminearum PH-1
0.00	1	0	G1	171627	Fusarium fujikuroi species complex
0.00	1	0	S	117187	Fusarium verticillioides
0.00	1	1	S1	334819	Fusarium verticillioides 7600
0.00	1	0	G1	171631	Fusarium oxysporum species complex
0.00	1	0	S	5507	Fusarium oxysporum
0.00	1	0	S1	59765	Fusarium oxysporum f. sp. lycopersici
0.00	1	1	S2	426428	Fusarium oxysporum f. sp. lycopersici 4287
0.00	1	0	F	5129	Hypocreaceae
0.00	1	1	G	5543	Trichoderma
0.00	1	0	F	474942	Ophiocordycipitaceae
0.00	1	0	G	1052105	Purpureocillium
0.00	1	1	S	2060973	Purpureocillium takamizusanense
0.00	1	0	O	1028384	Glomerellales
0.00	1	0	F	681950	Glomerellaceae
0.00	1	0	G	5455	Colletotrichum
0.00	1	0	G1	2707350	Colletotrichum destructivum species complex
0.00	1	1	S	34406	Colletotrichum destructivum
0.00	4	0	C1	222544	Sordariomycetidae
0.00	4	0	O	5139	Sordariales
0.00	2	1	F	35718	Chaetomiaceae
0.00	1	0	G	1920207	Thermothelomyces
0.00	1	0	S	78579	Thermothelomyces thermophilus
0.00	1	1	S1	573729	Thermothelomyces thermophilus ATCC 42464
0.00	2	0	F	2609812	Podosporaceae
0.00	2	1	G	5144	Podospora
0.00	1	1	S	2093780	Podospora pseudopauciseta

0.00	4	0	C	147548	Leotiomycetes
0.00	4	0	O	5178	Helotiales
0.00	4	0	F	28983	Sclerotiniaceae
0.00	4	0	G	33196	Botrytis
0.00	4	0	S	40559	Botrytis cinerea
0.00	4	4	S1	332648	Botrytis cinerea B05.10
0.02	25	0	P4	715962	dothideomyceta
0.02	25	0	C	147541	Dothideomycetes
0.02	20	0	C1	451868	Pleosporomycetidae
0.02	20	0	O	92860	Pleosporales
0.02	20	0	O1	715340	Pleosporineae
0.02	18	0	F	28556	Pleosporaceae
0.02	18	0	G	5027	Pyrenophora
0.02	18	18	S	45151	Pyrenophora tritici-repentis
0.00	2	0	F	683158	Didymellaceae
0.00	2	0	G	5453	Ascochyta
0.00	2	2	S	5454	Ascochyta rabiei
0.00	5	0	C1	451867	Dothideomycetidae
0.00	5	0	O	2726947	Mycosphaerellales
0.00	5	1	F	93133	Mycosphaerellaceae
0.00	2	0	G	29002	Cercospora
0.00	2	2	S	122368	Cercospora beticola
0.00	2	0	G	1047167	Zymoseptoria
0.00	2	0	S	1047171	Zymoseptoria tritici
0.00	2	2	S1	336722	Zymoseptoria tritici IPO323
0.01	11	0	C	147545	Eurotiomycetes
0.01	11	0	C1	451871	Eurotiomycetidae
0.01	6	0	O	33183	Onygenales
0.01	6	0	F	33184	Onygenaceae
0.01	6	0	G	5500	Coccidioides
0.01	6	0	S	199306	Coccidioides posadasii
0.01	6	6	S1	443226	Coccidioides posadasii str. Silveira
0.00	5	2	O	5042	Eurotiales
0.00	2	0	F	28568	Trichocomaceae
0.00	2	1	G	5094	Talaromyces
0.00	1	0	G1	2752542	Talaromyces sect. Islandici
0.00	1	1	S	121627	Talaromyces rugulosus
0.00	1	0	F	1131492	Aspergillaceae

0.00	1	0	G	5073	Penicillium
0.00	1	1	S	36651	Penicillium digitatum
0.01	12	1	P2	147537	Saccharomycotina
0.01	6	0	C	3239873	Dipodascomycetes
0.01	6	0	O	3243772	Dipodascales
0.01	6	0	O1	3316682	Dipodascales incertae sedis
0.01	6	0	G	4951	Yarrowia
0.01	6	6	S	4952	Yarrowia lipolytica
0.00	5	1	C	3239874	Pichiomycetes
0.00	2	0	O	2916678	Serinales
0.00	2	0	F	27319	Metschnikowiaceae
0.00	2	0	G	3303203	Candidozyma
0.00	2	2	S	498019	Candidozyma auris
0.00	2	0	O	3243775	Pichiales
0.00	2	0	F	1156497	Pichiaceae
0.00	2	0	G	13366	Brettanomyces
0.00	2	2	S	5007	Brettanomyces bruxellensis
0.00	1	0	P1	451866	Taphrinomycotina
0.00	1	0	C	147554	Schizosaccharomycetes
0.00	1	0	O	34346	Schizosaccharomycetales
0.00	1	0	F	4894	Schizosaccharomycetaceae
0.00	1	0	G	4895	Schizosaccharomyces
0.00	1	1	S	2545709	Schizosaccharomyces osmophilus
0.02	18	0	P	5204	Basidiomycota
0.01	11	0	P1	29000	Pucciniomycotina
0.01	11	0	C	162484	Pucciniomycetes
0.01	11	0	O	5258	Pucciniales
0.01	11	0	F	5262	Pucciniaceae
0.01	11	2	G	5296	Puccinia
0.01	7	0	S	27350	Puccinia striiformis
0.01	7	7	S1	168172	Puccinia striiformis f. sp. tritici
0.00	2	2	S	208348	Puccinia triticina
0.01	7	0	P1	5302	Agaricomycotina
0.01	7	0	C	155616	Tremellomycetes
0.01	7	0	O	5234	Tremellales
0.01	7	0	F	1884633	Cryptococcaceae
0.01	7	2	G	490731	Kwoniella
0.00	2	0	S	4975	Kwoniella dendrophila

0.00	2	2	S1	1295534	Kwoniella dendrophila CBS 6074
0.00	1	0	S	324769	Kwoniella bestiolae
0.00	1	1	S1	1296100	Kwoniella bestiolae CBS 10118
0.00	1	0	S	463800	Kwoniella mangrovensis
0.00	1	1	S1	1296122	Kwoniella mangroviensis CBS 8507
0.00	1	1	S	1734106	Kwoniella shandongensis
0.00	2	0	K1	112252	Fungi incertae sedis
0.00	2	0	P	1913637	Mucoromycota
0.00	2	0	P1	214504	Glomeromycotina
0.00	2	0	C	214506	Glomeromycetes
0.00	2	0	O	36750	Glomerales
0.00	2	0	F	36751	Glomeraceae
0.00	2	0	G	1129544	Rhizophagus
0.00	2	2	S	588596	Rhizophagus irregularis
0.01	10	1	D1	2698737	Sar
0.00	5	0	D2	33634	Stramenopiles
0.00	3	0	D3	2696291	Ochrophyta
0.00	3	0	P	2836	Bacillariophyta
0.00	2	0	C	33849	Bacillariophyceae
0.00	2	0	C1	33850	Bacillariophycidae
0.00	2	0	O	38748	Naviculales
0.00	2	0	F	38749	Phaeodactylaceae
0.00	2	0	G	2849	Phaeodactylum
0.00	2	0	S	2850	Phaeodactylum tricornutum
0.00	2	2	S1	556484	Phaeodactylum tricornutum CCAP 1055/1
0.00	1	0	C	33836	Coscinodiscophyceae
0.00	1	0	C1	33846	Thalassiosirophycidae
0.00	1	0	O	33847	Thalassiosirales
0.00	1	0	F	29202	Thalassiosiraceae
0.00	1	0	G	35127	Thalassiosira
0.00	1	0	S	35128	Thalassiosira pseudonana
0.00	1	1	S1	296543	Thalassiosira pseudonana CCMP1335
0.00	2	0	P	4762	Oomycota
0.00	2	0	O	4776	Peronosporales
0.00	2	0	F	4777	Peronosporaceae
0.00	2	0	G	4778	Bremia
0.00	2	2	S	4779	Bremia lactucae
0.00	4	0	D2	33630	Alveolata

0.00	4	0	P	5794	Apicomplexa
0.00	3	0	C	422676	Aconoidasida
0.00	2	0	O	5819	Haemosporida
0.00	2	0	F	1639119	Plasmodiidae
0.00	2	1	G	5820	Plasmodium
0.00	1	0	G1	418101	Plasmodium (Vinckeia)
0.00	1	0	S	5860	Plasmodium vinckei
0.00	1	1	S1	54757	Plasmodium vinckei vinckei
0.00	1	0	O	5863	Piroplasmida
0.00	1	0	F	32594	Babesiidae
0.00	1	0	G	5864	Babesia
0.00	1	1	S	5866	Babesia bigemina
0.00	1	0	C	1280412	Conoidasida
0.00	1	0	C1	5796	Coccidia
0.00	1	0	O	75739	Eucoccidiorida
0.00	1	0	O1	423054	Eimeriorina
0.00	1	0	F	5809	Sarcocystidae
0.00	1	0	G	5810	Toxoplasma
0.00	1	0	S	5811	Toxoplasma gondii
0.00	1	1	S1	508771	Toxoplasma gondii ME49
0.00	1	0	C	3027	Cryptophyceae
0.00	1	0	O	589342	Pyrenomonadales
0.00	1	0	F	589343	Geminigeraceae
0.00	1	0	G	55528	Guillardia
0.00	1	1	S	55529	Guillardia theta
0.14	161	21		2	Bacteria
0.05	63	0	P	1224	Pseudomonadota
0.04	43	7	C	1236	Gammaproteobacteria
0.02	26	1	O	91347	Enterobacterales
0.02	22	1	F	1903409	Erwiniaceae
0.02	18	7	G	551	Erwinia
0.01	11	11	S	68334	Erwinia aphidicola
0.00	3	2	G	53335	Pantoea
0.00	1	0	G1	1654067	Pantoea agglomerans group
0.00	1	1	S	549	Pantoea agglomerans
0.00	2	0	F	543	Enterobacteriaceae
0.00	1	0	G	561	Escherichia
0.00	1	1	S	208962	Escherichia albertii

0.00	1	0	F1	2890311	Klebsiella/Raoultella group
0.00	1	0	G	570	Klebsiella
0.00	1	1	S	548	Klebsiella aerogenes
0.00	1	0	F	1903414	Morganellaceae
0.00	1	0	G	586	Providencia
0.00	1	1	S	126385	Providencia alcalifaciens
0.01	7	0	O	2887326	Moraxellales
0.01	7	0	F	468	Moraxellaceae
0.00	5	2	G	469	Acinetobacter
0.00	2	0	G1	909768	Acinetobacter calcoaceticus/baumannii complex
0.00	1	1	S	470	Acinetobacter baumannii
0.00	1	1	S	48296	Acinetobacter pittii
0.00	1	1	G1	196816	unclassified Acinetobacter
0.00	2	2	G	475	Moraxella
0.00	2	0	O	72274	Pseudomonadales
0.00	2	0	F	135621	Pseudomonadaceae
0.00	2	0	G	286	Pseudomonas
0.00	2	0	G1	196821	unclassified Pseudomonas
0.00	1	1	S	2895471	Pseudomonas sp. B21-010
0.00	1	1	S	3019968	Pseudomonas sp. MM227
0.00	1	0	C1	118884	Gammaproteobacteria incertae sedis
0.00	1	0	C2	32036	sulfur-oxidizing symbionts
0.00	1	1	S	2360	Bathymodiolus thermophilus thioautotrophic gill symbiont
0.01	11	3	C	28216	Betaproteobacteria
0.01	8	1	O	80840	Burkholderiales
0.00	4	2	F	80864	Comamonadaceae
0.00	2	0	G	3051137	Paracidovorax
0.00	2	2	S	80867	Paracidovorax avenae
0.00	3	1	F	75682	Oxalobacteraceae
0.00	2	1	F1	2895353	Telluria group
0.00	1	1	G	149698	Massilia
0.01	9	0	C	28211	Alphaproteobacteria
0.00	4	0	O	766	Rickettsiales
0.00	2	0	F	775	Rickettsiaceae
0.00	2	0	F1	33988	Rickettsieae
0.00	2	2	G	780	Rickettsia
0.00	2	0	F	942	Anaplasmataceae
0.00	2	0	F1	952	Wolbachieae

0.00	2	0	G	953	Wolbachia
0.00	2	0	G1	2640676	unclassified Wolbachia
0.00	1	1	S	3066146	Wolbachia endosymbiont (group A) of Lypha dubia
0.00	1	1	S	2883238	Wolbachia endosymbiont of Chironomus riparius
0.00	4	0	O	356	Hyphomicrobiales
0.00	2	0	F	119045	Methylobacteriaceae
0.00	2	0	G	407	Methylobacterium
0.00	1	0	G1	2615210	unclassified Methylobacterium
0.00	1	1	S	2603276	Methylobacterium sp. WL1
0.00	1	1	S	570505	Methylobacterium bullatum
0.00	1	0	F	45401	Hyphomicrobiaceae
0.00	1	0	G	81	Hyphomicrobium
0.00	1	0	G1	2619925	unclassified Hyphomicrobium
0.00	1	1	S	3019544	Hyphomicrobium sp. DMF-1
0.00	1	0	F	69277	Phyllobacteriaceae
0.00	1	0	G	68287	Mesorhizobium
0.00	1	0	S	39645	Mesorhizobium ciceri
0.00	1	0	S1	278148	Mesorhizobium ciceri biovar biserrulae
0.00	1	1	S2	765698	Mesorhizobium ciceri biovar biserrulae WSM1271
0.00	1	0	O	1921002	Holosporales
0.00	1	0	F	44746	Holosporaceae
0.00	1	0	G	2601574	Candidatus Cytomitobacter
0.00	1	1	S	2066024	Candidatus Cytomitobacter primus
0.04	51	1	D1	1783272	Terrabacteria group
0.02	24	0	D2	1798711	Cyanobacteriota/Melainabacteria group
0.02	24	0	P	1117	Cyanobacteriota
0.02	24	4	C	3028117	Cyanophyceae
0.02	18	0	O	1890424	Synechococcales
0.01	15	0	F	1890428	Merismopediaceae
0.01	15	0	G	1142	Synechocystis
0.01	15	0	G1	2640012	unclassified Synechocystis
0.01	15	15	S	3144951	Synechocystis sp. LKSZ1
0.00	3	0	F	1890426	Synechococcaceae
0.00	3	0	G	1129	Synechococcus
0.00	3	2	G1	2626047	unclassified Synechococcus
0.00	1	1	S	166314	Synechococcus sp. WH 8109
0.00	1	0	O	1161	Nostocales
0.00	1	0	F	1162	Nostocaceae

0.00	1	0	G	1177	Nostoc
0.00	1	0	G1	2593658	unclassified Nostoc
0.00	1	1	S	2576904	Nostoc sp. TCL26-01
0.00	1	0	C1	1301283	Oscillatoriophycideae
0.00	1	0	O	1118	Chroococcales
0.00	1	1	F	2815910	Geminocystaceae
0.02	18	4	P	1239	Bacillota
0.01	9	0	C	91061	Bacilli
0.01	6	0	O	1385	Bacillales
0.00	3	0	F	186822	Paenibacillaceae
0.00	2	0	G	44249	Paenibacillus
0.00	1	0	G1	185978	unclassified Paenibacillus
0.00	1	1	S	2859228	Paenibacillus sp. R14(2021)
0.00	1	1	S	59893	Paenibacillus peoriae
0.00	1	0	G	55080	Brevibacillus
0.00	1	1	S	2496837	Brevibacillus marinus
0.00	2	0	F	186817	Bacillaceae
0.00	1	0	G	1386	Bacillus
0.00	1	0	G1	86661	Bacillus cereus group
0.00	1	1	S	2026186	Bacillus paranthracis
0.00	1	1	G	2837504	Heyndrickxia
0.00	1	0	F	90964	Staphylococcaceae
0.00	1	0	G	1279	Staphylococcus
0.00	1	0	G1	2815305	Staphylococcus intermedius group
0.00	1	1	S	283734	Staphylococcus pseudintermedius
0.00	3	0	O	186826	Lactobacillales
0.00	2	0	F	1300	Streptococcaceae
0.00	2	2	G	1301	Streptococcus
0.00	1	0	F	33958	Lactobacillaceae
0.00	1	0	G	2767887	Ligilactobacillus
0.00	1	1	S	1601	Ligilactobacillus agilis
0.00	2	0	C	186801	Clostridia
0.00	1	0	O	68295	Thermoanaerobacterales
0.00	1	0	F	186814	Thermoanaerobacteraceae
0.00	1	0	G	28895	Thermoanaerobacterium
0.00	1	0	G1	2622527	unclassified Thermoanaerobacterium
0.00	1	1	S	1550240	Thermoanaerobacterium sp. RBIITD
0.00	1	0	O	186802	Eubacteriales

0.00	1	0	F	31979	Clostridiaceae
0.00	1	0	G	1485	Clostridium
0.00	1	1	S	46867	Clostridium chauvoei
0.00	2	0	C	909932	Negativicutes
0.00	2	0	O	909929	Selenomonadales
0.00	2	0	F	1843490	Sporomusaceae
0.00	2	0	G	2375	Sporomusa
0.00	1	0	S	47679	Sporomusa sphaeroides
0.00	1	1	S1	1337886	Sporomusa sphaeroides DSM 2875
0.00	1	0	G1	307248	environmental samples
0.00	1	1	S	307249	uncultured Sporomusa sp.
0.00	1	0	C	1737404	Tissierellia
0.00	1	0	O	1737405	Tissierellales
0.00	1	0	F	1570339	Peptoniphilaceae
0.00	1	1	G	162289	Peptoniphilus
0.01	7	0	P	201174	Actinomycetota
0.01	7	0	C	1760	Actinomycetes
0.00	2	0	O	85006	Micrococcales
0.00	1	0	F	85019	Brevibacteriaceae
0.00	1	1	G	1696	Brevibacterium
0.00	1	1	F	85023	Microbacteriaceae
0.00	2	0	O	85009	Propionibacteriales
0.00	1	0	F	31957	Propionibacteriaceae
0.00	1	0	G	2801844	Arachnia
0.00	1	1	S	1547448	Arachnia rubra
0.00	1	0	F	85015	Nocardioidaceae
0.00	1	0	G	1546255	Mumia
0.00	1	1	G1	2621872	unclassified Mumia
0.00	2	0	O	85011	Kitasatosporales
0.00	2	0	F	2062	Streptomycetaceae
0.00	2	0	G	1883	Streptomyces
0.00	1	1	S	1442032	Streptomyces dangxiongensis
0.00	1	0	G1	2593676	unclassified Streptomyces
0.00	1	1	S	477697	Streptomyces sp. T12
0.00	1	0	O	2037	Actinomycetales
0.00	1	0	F	2049	Actinomycetaceae
0.00	1	1	G	1654	Actinomyces
0.00	1	0	P	1297	Deinococcota

0.00	1	0	C	188787	Deinococci
0.00	1	0	O	118964	Deinococcales
0.00	1	0	F	183710	Deinococcaceae
0.00	1	1	G	1298	Deinococcus
0.01	15	0	D1	1783270	FCB group
0.01	15	0	D2	68336	Bacteroidota/Chlorobiota group
0.01	15	0	P	976	Bacteroidota
0.01	8	0	C	117743	Flavobacteriia
0.01	8	0	O	200644	Flavobacteriales
0.00	4	0	F	49546	Flavobacteriaceae
0.00	3	2	G	237	Flavobacterium
0.00	1	1	G1	196869	unclassified Flavobacterium
0.00	1	0	G	363408	Nonlabens
0.00	1	1	S	906888	Nonlabens ulvanivorans
0.00	3	1	F	2762318	Weeksellaceae
0.00	1	0	G	28250	Ornithobacterium
0.00	1	1	S	28251	Ornithobacterium rhinotracheale
0.00	1	0	G	59734	Empedobacter
0.00	1	1	S	1628248	Empedobacter stercoris
0.00	1	0	F	39782	Blattabacteriaceae
0.00	1	0	G	34098	Blattabacterium
0.00	1	1	S	1653831	Blattabacterium cuenoti
0.01	6	0	C	768503	Cytophagia
0.01	6	0	O	768507	Cytophagales
0.00	2	0	F	1853232	Hymenobacteraceae
0.00	1	1	G	89966	Hymenobacter
0.00	1	1	G	299566	Adhaeribacter
0.00	1	0	F	1937968	Bernardetiaceae
0.00	1	0	G	1937972	Bernardetia
0.00	1	0	S	999	Bernardetia litoralis
0.00	1	1	S1	880071	Bernardetia litoralis DSM 6794
0.00	1	0	F	3141702	Leadbetterellaceae
0.00	1	0	G	2975149	Marinilongibacter
0.00	1	1	S	2975157	Marinilongibacter aquaticus
0.00	1	0	F	2896860	Spirosomataceae
0.00	1	0	G	861914	Fibrella
0.00	1	0	S	651143	Fibrella aestuarina
0.00	1	1	S1	1166018	Fibrella aestuarina BUZ 2

0.00	1	0	F	2858885	Mangrovivirgaceae
0.00	1	0	G	2858886	Mangrovivirga
0.00	1	1	S	2715131	Mangrovivirga cuniculi
0.00	1	0	C	117747	Sphingobacteriia
0.00	1	0	O	200666	Sphingobacteriales
0.00	1	0	F	84566	Sphingobacteriaceae
0.00	1	1	G	28453	Sphingobacterium
0.00	3	0	P	29547	Campylobacterota
0.00	3	0	C	3031852	Epsilonproteobacteria
0.00	3	0	O	213849	Campylobacterales
0.00	1	0	F	72293	Helicobacteraceae
0.00	1	0	G	209	Helicobacter
0.00	1	1	S	210	Helicobacter pylori
0.00	1	0	F	72294	Campylobacteraceae
0.00	1	1	G	194	Campylobacter
0.00	1	0	F	2771472	Sulfurovaceae
0.00	1	0	G	269260	Nitratifractor
0.00	1	0	S	269261	Nitratifractor salsuginis
0.00	1	1	S1	749222	Nitratifractor salsuginis DSM 16511
0.00	3	0	P	32066	Fusobacteriota
0.00	3	0	C	203490	Fusobacteriia
0.00	3	0	O	203491	Fusobacteriales
0.00	2	0	F	203492	Fusobacteriaceae
0.00	2	0	G	848	Fusobacterium
0.00	2	2	S	2764326	Fusobacterium hominis
0.00	1	0	F	1129771	Leptotrichiaceae
0.00	1	0	G	32067	Leptotrichia
0.00	1	0	G1	2633022	unclassified Leptotrichia
0.00	1	1	S	3239303	Leptotrichia sp. HSP-342
0.00	2	0	P	200940	Thermodesulfobacteriota
0.00	2	0	C	3031449	Desulfovibrionia
0.00	2	0	O	213115	Desulfovibrionales
0.00	2	0	F	194924	Desulfovibrionaceae
0.00	2	1	G	872	Desulfovibrio
0.00	1	0	G1	2593640	unclassified Desulfovibrio
0.00	1	1	S	631220	Desulfovibrio sp. G11
0.00	1	0	P	203691	Spirochaetota
0.00	1	0	C	203692	Spirochaetia

0.00	1	0	O	1643686	Brachyspirales
0.00	1	0	F	143786	Brachyspiraceae
0.00	1	0	G	29521	Brachyspira
0.00	1	1	S	159	Brachyspira hyodysenteriae
0.00	1	0	D1	1783257	PVC group
0.00	1	0	P	204428	Chlamydiota
0.00	1	0	C	204429	Chlamydiia
0.00	1	0	O	51291	Chlamydiales
0.00	1	0	F	809	Chlamydiaceae
0.00	1	0	F1	1113537	Chlamydia/Chlamydophila group
0.00	1	0	G	810	Chlamydia
0.00	1	1	S	83559	Chlamydia suis
0.00	1	0	P	200918	Thermotogota
0.00	1	0	C	188708	Thermotogae
0.00	1	0	O	1643947	Petrotogales
0.00	1	0	F	1643949	Petrotogaceae
0.00	1	0	G	2778400	Tepiditoga
0.00	1	1	S	2108365	Tepiditoga spiralis
0.00	2	0		2157	Archaea
0.00	2	0	P	28890	Euryarchaeota
0.00	1	0	P1	2283794	Methanomada group
0.00	1	0	C	183939	Methanococci
0.00	1	0	O	2182	Methanococcales
0.00	1	0	F	196117	Methanocaldococcaceae
0.00	1	0	G	196118	Methanocaldococcus
0.00	1	1	S	1301915	Methanocaldococcus bathoardescens
0.00	1	0	P1	2290931	Stenosarchaea group
0.00	1	0	C	183963	Halobacteria
0.00	1	0	O	2235	Halobacteriales
0.00	1	0	F	3064797	Haladaptataceae
0.00	1	0	G	367188	Haladaptatus
0.00	1	1	S	2884876	Haladaptatus salinisoli
0.00	4	0		10239	Viruses
0.00	4	0		185751	Pospiviroidae
0.00	3	0	G	147262	Hostuviroid
0.00	3	3	S	12893	Hop stunt viroid
0.00	1	0	G	185756	Apscaviroid
0.00	1	1	S	12904	Grapevine yellow speckle viroid 1
