# Supplementary material for: Nanopore Workflow for Grapevine Viroid Surveillance in Kazakhstan: Bypassing rRNA Depletion Through Non-Canonical Priming
Source: Pathogens. 2025 Aug 6;14(8):782. doi: 10.3390/pathogens14080782 (PMC12388932; doi:10.3390/pathogens14080782)
Supplement: Supplementary file 1 [file pathogens-14-00782-s001.zip › Figure S4.pdf]

5.17	108797	108797	U	0	unclassified
94.83	1994066	5	R	1	root
94.82	1993936	11039	1	131567	cellular organisms
94.13	1979407	7380		2759	Eukaryota
92.77	1950762	46	K	33090	Viridiplantae
92.76	1950696	0	P	35493	Streptophyta
92.76	1950696	0	P1	131221	Streptophytina
92.76	1950696	1926	P2	3193	Embryophyta
92.67	1948740	0	P3	58023	Tracheophyta
92.67	1948740	0	P4	78536	Euphyllophyta
92.67	1948740	4548	P5	58024	Spermatophyta
92.41	1943159	31168	C	3398	Magnoliopsida
90.84	1910248	139489	C1	1437183	Mesangiospermae
76.35	1605624	0	C2	71240	eudicotyledons
76.35	1605624	0	C3	91827	Gunneridae
76.35	1605624	101069	C4	1437201	Pentapetalae
66.19	1391980	50011	C5	71275	rosids
50.88	1069901	0	C6	91834	rosids incertae sedis
50.88	1069901	0	O	403667	Vitales
50.88	1069901	0	F	3602	Vitaceae
50.88	1069901	0	F1	2304100	Viteae
50.88	1069901	431262	G	3603	Vitis
17.83	374960	374960	S	96939	Vitis riparia
12.54	263679	263679	S	29760	Vitis vinifera
8.42	177066	25254	C6	91835	fabids
2.00	42150	0	O	72025	Fabales
2.00	42150	0	F	3803	Fabaceae
2.00	42150	0	F1	3814	Papilionoideae
2.00	42150	830	F2	2231393	50 kb inversion clade
0.97	20467	0	F3	2231387	dalbergioids sensu lato
0.97	20467	0	F4	163725	Dalbergieae
0.97	20467	0	F5	2231390	Pterocarpus clade
0.97	20467	2452	G	3817	Arachis
0.73	15388	15388	S	217475	Arachis stenosperma
0.06	1234	1234	S	3818	Arachis hypogaea
0.05	1145	1145	S	130453	Arachis duranensis
0.01	248	248	S	130454	Arachis ipaensis
0.87	18395	106	F3	2231382	NPAAA clade

0.51	10677	22	F4	2233838	Hologalegina
0.44	9298	1046	F5	2233839	IRL clade
0.19	3926	0	F6	163722	Cicereae
0.19	3926	0	G	3826	Cicer
0.19	3926	3926	S	3827	Cicer arietinum
0.11	2416	8	F6	163743	Fabeae
0.10	2202	0	G	3887	Pisum
0.10	2202	2202	S	3888	Pisum sativum
0.01	206	0	G	3904	Vicia
0.01	206	206	S	3911	Vicia villosa
0.09	1910	1	F6	163742	Trifolieae
0.08	1621	0	G	3898	Trifolium
0.08	1621	1621	S	57577	Trifolium pratense
0.01	288	0	G	3877	Medicago
0.01	288	288	S	3880	Medicago truncatula
0.06	1357	0	F5	2233857	robinioid clade
0.06	1357	0	F6	163747	Loteae
0.06	1357	0	G	3867	Lotus
0.06	1357	1357	S	34305	Lotus japonicus
0.36	7612	0	F4	2233855	indigoferoid/millettioid clade
0.36	7612	172	F5	163735	Phaseoleae
0.22	4604	10	G	3913	Vigna
0.10	2198	2198	S	3917	Vigna unguiculata
0.10	2021	0	S	157791	Vigna radiata
0.10	2021	2021	S1	3916	Vigna radiata var. radiata
0.02	375	375	S	3914	Vigna angularis
0.07	1568	0	G	3846	Glycine
0.07	1568	1093	G1	1462606	Glycine subgen. Soja
0.02	418	418	S	3847	Glycine max
0.00	57	57	S	3848	Glycine soja
0.04	806	0	G	3820	Cajanus
0.04	806	806	S	3821	Cajanus cajan
0.02	462	0	G	3883	Phaseolus
0.02	462	462	S	3885	Phaseolus vulgaris
0.12	2458	0	F3	2231384	genistoids sensu lato
0.12	2458	0	F4	2231385	core genistoids

0.12	2458	0	F5	163729	Genisteae
0.12	2458	0	G	3869	Lupinus
0.12	2458	2458	S	3871	Lupinus angustifolius
1.46	30679	0	O	71239	Cucurbitales
1.46	30679	289	F	3650	Cucurbitaceae
1.27	26701	497	F1	1003877	Benincaseae
0.82	17264	40	G	3655	Cucumis
0.78	16357	16357	S	3656	Cucumis melo
0.04	867	867	S	3659	Cucumis sativus
0.43	8940	0	G	102210	Benincasa
0.43	8940	8940	S	102211	Benincasa hispida
0.18	3689	0	F1	1003878	Cucurbiteae
0.18	3689	0	G	3660	Cucurbita
0.18	3689	0	S	3663	Cucurbita pepo
0.18	3689	3689	S1	3664	Cucurbita pepo subsp. pepo
1.29	27199	937	O	3502	Fagales
0.72	15046	824	F	16714	Juglandaceae
0.64	13411	131	G	16718	Juglans
0.57	12090	12090	S	51240	Juglans regia
0.06	1190	1190	S	2249226	Juglans microcarpa x Juglans regia
0.04	811	0	G	13402	Carya
0.04	811	811	S	32201	Carya illinoinensis
0.37	7681	0	F	3503	Fagaceae
0.37	7681	439	G	3511	Quercus
0.26	5444	5444	S	38942	Quercus robur
0.09	1798	1798	S	97700	Quercus lobata
0.17	3535	14	F	3514	Betulaceae
0.14	2882	0	G	3515	Alnus
0.14	2882	2882	S	3517	Alnus glutinosa
0.03	639	0	G	13450	Corylus
0.03	639	639	S	13451	Corylus avellana
1.27	26686	263	O	3744	Rosales
0.76	15976	3259	F	3745	Rosaceae
0.33	6966	145	F1	171637	Amygdaloideae
0.22	4707	0	F2	721813	Maleae
0.22	4707	1995	G	3749	Malus
0.07	1563	1563	S	3752	Malus sylvestris

0.05	1149	1149	S	3750	Malus domestica
0.10	2114	0	F2	721805	Amygdaleae
0.10	2114	429	G	3754	Prunus
0.03	630	630	S	3760	Prunus persica
0.03	538	538	S	3755	Prunus dulcis
0.02	517	517	S	102107	Prunus mume
0.27	5751	538	F1	171638	Rosoideae
0.16	3372	0	F2	1176516	Rosoideae incertae sedis
0.16	3372	875	G	3764	Rosa
0.07	1399	1399	S	74645	Rosa rugosa
0.05	1098	1098	S	74649	Rosa chinensis
0.09	1841	47	F2	721789	Potentilleae
0.05	1151	0	F3	1184124	Fragariinae
0.05	1151	0	G	3746	Fragaria
0.05	1151	0	S	57918	Fragaria vesca
0.05	1151	1151	S1	101020	Fragaria vesca subsp. vesca
0.03	643	0	F3	1184125	Potentilleae incertae sedis
0.03	643	0	G	23204	Potentilla
0.03	643	643	S	57926	Argentina anserina
0.27	5772	89	F	3481	Cannabaceae
0.15	3220	0	G	3484	Humulus
0.15	3220	3220	S	3486	Humulus lupulus
0.12	2463	0	G	3482	Cannabis
0.12	2463	2463	S	3483	Cannabis sativa
0.22	4675	0	F	3608	Rhamnaceae
0.22	4675	0	F1	325284	Paliureae
0.22	4675	0	G	72171	Ziziphus
0.22	4675	4675	S	326968	Ziziphus jujuba
0.97	20427	111	O	3646	Malpighiales
0.78	16302	166	F	3977	Euphorbiaceae
0.45	9491	0	F1	235629	Acalyphoideae
0.45	9491	31	F2	235880	Acalypheae
0.23	4874	0	G	3987	Ricinus
0.23	4874	4874	S	3988	Ricinus communis
0.22	4586	0	G	3984	Mercurialis
0.22	4586	4586	S	3986	Mercurialis annua
0.17	3515	48	F1	235631	Crotonoideae

0.10	2104	0	F2	235883	Manihoteae
0.10	2104	0	G	3982	Manihot
0.10	2104	2104	S	3983	Manihot esculenta
0.06	1363	0	F2	235882	Micrandreae
0.06	1363	0	G	3980	Hevea
0.06	1363	1363	S	3981	Hevea brasiliensis
0.15	3130	0	F1	235633	Euphorbioideae
0.15	3130	0	F2	235895	Euphorbieae
0.15	3130	0	G	3990	Euphorbia
0.15	3130	0	G1	1334278	Euphorbia subgen. Esula
0.15	3130	0	G2	1334446	Euphorbia sect. Lathyris
0.15	3130	3130	S	212925	Euphorbia lathyris
0.19	4014	0	F	3688	Salicaceae
0.19	4014	0	F1	238069	Saliceae
0.19	4014	394	G	3689	Populus
0.10	2180	2180	S	3691	Populus nigra
0.07	1440	1440	S	3694	Populus trichocarpa
0.22	4671	0	O	233875	Celastrales
0.22	4671	0	F	4305	Celastraceae
0.22	4671	0	G	123484	Tripterygium
0.22	4671	4671	S	458696	Tripterygium wilfordii
4.52	95002	4621	C6	91836	malvids
1.93	40579	0	O	41938	Malvales
1.93	40579	40	F	3629	Malvaceae
1.93	40482	0	F1	214907	Malvoideae
1.93	40482	5371	G	3633	Gossypium
0.81	17063	17063	S	29729	Gossypium arboreum
0.49	10230	10230	S	29730	Gossypium raimondii
0.37	7818	7818	S	3635	Gossypium hirsutum
0.00	57	0	F1	214909	Byttnerioideae
0.00	57	0	G	3640	Theobroma
0.00	57	57	S	3641	Theobroma cacao
1.83	38414	0	O	3699	Brassicales
1.83	38414	32	F	3700	Brassicaceae
1.79	37577	916	F1	981071	Brassiceae
1.68	35272	5677	G	3705	Brassica
1.25	26267	26267	S	3708	Brassica napus
0.12	2533	2533	S	3711	Brassica rapa

0.04	795	0	S	3712	Brassica oleracea
0.04	795	795	S1	109376	Brassica oleracea var. oleracea
0.07	1389	0	G	3725	Raphanus
0.07	1389	1389	S	3726	Raphanus sativus
0.04	805	3	F1	980083	Camelineae
0.04	755	0	G	71323	Camelina
0.04	755	755	S	90675	Camelina sativa
0.00	47	0	G	3701	Arabidopsis
0.00	47	47	S	3702	Arabidopsis thaliana
0.41	8521	83	O	41944	Myrtales
0.34	7227	0	F	3931	Myrtaceae
0.34	7227	537	F1	1699513	Myrtoideae
0.21	4412	0	F2	1699524	Eucalypteae
0.21	4412	0	G	3932	Eucalyptus
0.21	4412	4412	S	71139	Eucalyptus grandis
0.11	2278	0	F2	1699523	Myrteae
0.11	2278	0	F3	1705102	Australasian group
0.11	2278	0	G	178132	Rhodamnia
0.11	2278	2278	S	178133	Rhodamnia argentea
0.06	1211	0	F	3928	Lythraceae
0.06	1211	0	G	22662	Punica
0.06	1211	1211	S	22663	Punica granatum
0.14	2867	3	O	41937	Sapindales
0.12	2532	0	F	4011	Anacardiaceae
0.12	2532	0	G	23461	Mangifera
0.12	2532	2532	S	29780	Mangifera indica
0.02	332	0	F	23513	Rutaceae
0.02	332	0	F1	1728959	Aurantioideae
0.02	332	0	G	2706	Citrus
0.02	332	332	S	2711	Citrus sinensis
4.26	89607	7110	C5	71274	asterids
2.68	56405	1851	C6	91888	lamiids
2.17	45573	149	O	4069	Solanales
2.09	43988	2242	F	4070	Solanaceae
1.55	32540	4110	F1	424551	Solanoideae
0.63	13161	0	F2	424574	Solaneae
0.63	13161	409	G	4107	Solanum

0.26	5438	2581	G1	49274	Solanum subgen. Lycopersicon
0.09	1887	1887	S	4081	Solanum lycopersicum
0.05	970	970	S	28526	Solanum pennellii
0.21	4368	4368	S	172797	Solanum stenotomum
0.14	2946	2946	S	45834	Solanum dulcamara
0.46	9650	0	F2	424564	Capsiceae
0.46	9650	0	G	4071	Capsicum
0.46	9650	9650	S	4072	Capsicum annuum
0.27	5619	0	F2	424569	Lycieae
0.27	5619	1042	G	24646	Lycium
0.13	2753	2753	S	112874	Lycium ferocissimum
0.09	1824	1824	S	112863	Lycium barbarum
0.44	9206	0	F1	424554	Nicotianoideae
0.44	9206	0	F2	424562	Nicotianeae
0.44	9206	0	G	4085	Nicotiana
0.44	9206	9206	S	49451	Nicotiana attenuata
0.07	1436	0	F	4118	Convolvulaceae
0.07	1436	0	F1	267213	Ipomoeeae
0.07	1436	0	G	4119	Ipomoea
0.07	1436	1436	S	35885	Ipomoea triloba
0.31	6430	48	O	4143	Lamiales
0.21	4356	0	F	4144	Oleaceae
0.21	4356	0	F1	426106	Oleeae
0.21	4356	0	G	4145	Olea
0.21	4356	0	S	4146	Olea europaea
0.21	4356	0	S1	158383	Olea europaea subsp. europaea
0.21	4356	4356	S2	158386	Olea europaea var. sylvestris
0.06	1270	0	F	4136	Lamiaceae
0.06	1270	0	F1	216706	Nepetoideae
0.06	1270	0	F2	216718	Mentheae
0.06	1270	0	F3	2836339	Salviinae
0.06	1270	11	G	21880	Salvia
0.03	635	0	G1	2291027	Salvia incertae sedis
0.02	426	426	S	49212	Salvia hispanica
0.01	209	209	S	226208	Salvia miltiorrhiza
0.03	624	0	G1	2026555	Salvia subgen. Calosphace
0.03	624	0	G2	2026556	core Calosphace

0.03	624	624	S	180675	Salvia splendens
0.04	756	0	F	4180	Pedaliaceae
0.04	756	0	G	4181	Sesamum
0.04	756	756	S	4182	Sesamum indicum
0.12	2551	0	O	4055	Gentianales
0.12	2551	0	F	24966	Rubiaceae
0.12	2551	0	F1	169618	Ixoroideae
0.12	2551	0	F2	1968429	Gardenieae complex
0.12	2551	0	F3	1968428	Bertiereae - Coffeeae clade
0.12	2551	0	F4	169640	Coffeeae
0.12	2551	1150	G	13442	Coffea
0.06	1199	1199	S	13443	Coffea arabica
0.01	202	202	S	49369	Coffea eugenioides
0.97	20298	22	C6	91882	campanulids
0.88	18576	0	O	4209	Asterales
0.88	18576	948	F	4210	Asteraceae
0.41	8577	0	F1	219103	Carduoideae
0.41	8577	0	F2	102818	Cardueae
0.41	8577	0	F3	742010	Carduinae
0.41	8577	0	G	4264	Cynara
0.41	8577	0	S	4265	Cynara cardunculus
0.41	8577	0	S1	309979	Cynara cardunculus subsp. carduncululus
0.41	8577	8577	S2	59895	Cynara cardunculus var. scolymus
0.22	4599	7	F1	102804	Asteroideae
0.12	2552	0	F2	911341	Heliantheae alliance
0.12	2552	0	F3	102814	Heliantheae
0.12	2552	0	G	4231	Helianthus
0.12	2552	2552	S	4232	Helianthus annuus
0.10	2040	0	F2	102809	Astereae
0.10	2040	0	F3	877976	North American clade
0.10	2040	0	F4	2841728	Conyzinae
0.10	2040	0	G	41574	Erigeron
0.10	2040	2040	S	72917	Erigeron canadensis
0.21	4452	0	F1	219120	Cichorioideae
0.21	4452	0	F2	219121	Cichorieae
0.21	4452	0	F3	745062	Lactucinae

0.21	4452	0	G	4235	Lactuca
0.21	4452	4452	S	4236	Lactuca sativa
0.08	1700	0	O	4036	Apiales
0.08	1700	0	O1	364270	Apiineae
0.08	1700	0	F	4037	Apiaceae
0.08	1700	0	F1	241778	Apioideae
0.08	1700	0	F2	241789	Scandiceae
0.08	1700	0	F3	241799	Daucinae
0.08	1700	0	G	4038	Daucus
0.08	1700	0	G1	1873447	Daucus sect. Daucus
0.08	1700	0	S	4039	Daucus carota
0.08	1700	1700	S1	79200	Daucus carota subsp. sativus
0.28	5794	501	O	41945	Ericales
0.12	2521	0	F	3623	Actinidiaceae
0.12	2521	0	G	3624	Actinidia
0.12	2521	2521	S	165200	Actinidia eriantha
0.05	986	0	F	25692	Balsaminaceae
0.05	986	0	G	35939	Impatiens
0.05	986	986	S	253017	Impatiens glandulifera
0.05	964	0	F	19955	Ebenaceae
0.05	964	0	G	13492	Diospyros
0.05	964	964	S	55363	Diospyros lotus
0.04	822	0	F	4345	Ericaceae
0.04	822	0	F1	217035	Ericoideae
0.04	822	0	F2	217046	Rhodoreae
0.04	822	0	G	4346	Rhododendron
0.04	822	822	S	182163	Rhododendron vialii
0.81	16953	0	O	41947	Santalales
0.81	16953	0	F	1003242	Ximeniaceae
0.81	16953	0	G	397391	Malania
0.81	16953	16953	S	397392	Malania oleifera
0.29	6015	52	O	3524	Caryophyllales
0.19	4085	37	F	1804623	Chenopodiaceae
0.13	2667	0	F1	1307796	Chenopodioideae
0.13	2667	0	F2	1307775	Anserineae
0.13	2667	0	G	3561	Spinacia
0.13	2667	2667	S	3562	Spinacia oleracea

0.07	1381	0	F1	1804621	Betoideae
0.07	1381	0	G	3554	Beta
0.07	1381	0	S	161934	Beta vulgaris
0.07	1381	1381	S1	3555	Beta vulgaris subsp. vulgaris
0.09	1878	0	F	3563	Amaranthaceae
0.09	1878	0	G	3564	Amaranthus
0.09	1878	1878	S	29722	Amaranthus tricolor
4.39	92383	0	C2	4447	Liliopsida
4.39	92383	308	C3	1437197	Petrosaviidae
4.13	86862	766	C4	4734	commelinids
3.78	79462	48	O	38820	Poales
3.76	79168	1603	F	4479	Poaceae
3.00	63160	152	F1	359160	BOP clade
2.97	62352	208	F2	147368	Pooideae
2.15	45176	0	F3	1648038	Triticodae
2.15	45176	1150	F4	147389	Triticeae
1.21	25385	2887	F5	1648030	Triticinae
1.03	21562	5987	G	4564	Triticum
0.52	10966	10966	S	85692	Triticum dicoccoides
0.16	3327	3327	S	4565	Triticum aestivum
0.06	1282	1282	S	4572	Triticum urartu
0.04	936	0	G	4480	Aegilops
0.04	936	0	S	37682	Aegilops tauschii
0.04	936	936	S1	200361	Aegilops tauschii subsp. strangulata
0.89	18641	0	F5	1648017	Hordeinae
0.89	18641	0	G	4512	Hordeum
0.89	18641	0	S	4513	Hordeum vulgare
0.89	18641	18641	S1	112509	Hordeum vulgare subsp. vulgare
0.80	16827	0	F3	1648037	Poodae
0.80	16827	0	F4	147387	Poeae
0.80	16827	0	F5	1652081	Poeae Chloroplast Group 2 (Poeae type)
0.80	16827	0	F6	2948571	Loliodinae
0.80	16827	0	F7	640630	Loliinae
0.80	16827	133	G	4520	Lolium
0.73	15269	15269	S	89674	Lolium rigidum

0.07	1425	1425	S	4522	Lolium perenne
0.01	141	0	F3	2822797	Stipodae
0.01	141	0	F4	147385	Brachypodieae
0.01	141	0	G	15367	Brachypodium
0.01	141	141	S	15368	Brachypodium distachyon
0.03	656	0	F2	147367	Oryzoideae
0.03	656	0	F3	147380	Oryzeae
0.03	656	0	F4	1648021	Oryzinae
0.03	656	129	G	4527	Oryza
0.01	214	0	S	4530	Oryza sativa
0.01	214	214	S1	39947	Oryza sativa Japonica Group
0.01	170	170	S	4538	Oryza glaberrima
0.01	143	143	S	4533	Oryza brachyantha
0.69	14405	17	F1	147370	PACMAD clade
0.63	13185	321	F2	147369	Panicoideae
0.48	10015	0	F3	1648033	Andropogonodae
0.48	10015	30	F4	147429	Andropogoneae
0.44	9345	0	F5	1648029	Tripsacinae
0.44	9345	0	G	4575	Zea
0.44	9345	9345	S	4577	Zea mays
0.02	469	0	F5	1648028	Sorghinae
0.02	469	0	G	4557	Sorghum
0.02	469	469	S	4558	Sorghum bicolor
0.01	171	0	F5	1648026	Saccharinae
0.01	171	0	G	62336	Miscanthus
0.01	171	171	S	154761	Miscanthus floridulus
0.14	2849	0	F3	1648036	Panicodae
0.14	2849	0	F4	147428	Paniceae
0.12	2614	0	F5	1293365	Panicinae
0.12	2614	27	G	4539	Panicum
0.09	1997	0	G1	2100772	Panicum sect. Panicum
0.09	1997	1997	S	206008	Panicum hallii
0.03	590	0	G1	2100771	Panicum sect. Hiantes
0.03	590	590	S	38727	Panicum virgatum
0.01	235	0	F5	1293361	Cenchrinae
0.01	235	34	G	4554	Setaria
0.01	147	147	S	4555	Setaria italica
0.00	54	54	S	4556	Setaria viridis

0.06	1203	0	F2	156631	Arundinoideae
0.06	1203	0	F3	1648043	Molinieae
0.06	1203	0	F4	2949676	Molininae
0.06	1203	0	G	15745	Phragmites
0.06	1203	1203	S	29695	Phragmites australis
0.01	246	0	F	4613	Bromeliaceae
0.01	246	0	F1	1909378	Bromelioideae
0.01	246	0	G	4614	Ananas
0.01	246	246	S	4615	Ananas comosus
0.18	3867	143	O	4618	Zingiberales
0.14	2911	0	F	4637	Musaceae
0.14	2911	0	G	4640	Musa
0.14	2911	0	S	4641	Musa acuminata
0.14	2911	2911	S1	214697	Musa acuminata AAA Group
0.04	813	0	F	4642	Zingiberaceae
0.04	813	0	G	4650	Zingiber
0.04	813	813	S	94328	Zingiber officinale
0.13	2767	0	O	40551	Arecales
0.13	2767	68	F	4710	Arecaceae
0.09	1823	0	F1	169697	Arecoideae
0.09	1823	0	F2	169705	Cocoseae
0.09	1823	0	F3	169729	Elaeidinae
0.09	1823	0	G	51952	Elaeis
0.09	1823	1823	S	51953	Elaeis guineensis
0.04	876	0	F1	169700	Coryphoideae
0.04	876	0	F2	169748	Phoeniceae
0.04	876	0	G	4719	Phoenix
0.04	876	876	S	42345	Phoenix dactylifera
0.22	4568	0	O	40548	Dioscoreales
0.22	4568	0	F	4671	Dioscoreaceae
0.22	4568	0	G	4672	Dioscorea
0.22	4568	0	S	29710	Dioscorea cayenensis
0.22	4568	4568	S1	55577	Dioscorea cayenensis subsp. rotundata
0.03	645	0	O	73496	Asparagales
0.03	645	0	F	40552	Asparagaceae
0.03	645	0	F1	703533	Asparagoideae
0.03	645	0	G	4685	Asparagus
0.03	645	645	S	4686	Asparagus officinalis

2.19	45990	0	O	41768	Ranunculales
2.19	45990	0	F	3465	Papaveraceae
2.19	45990	0	F1	1462614	Papaveroideae
2.19	45990	0	G	3468	Papaver
2.19	45990	45990	S	3469	Papaver somniferum
0.78	16401	0	C2	232347	Magnoliidae
0.78	16401	0	O	3400	Magnoliales
0.78	16401	0	F	3401	Magnoliaceae
0.78	16401	0	G	3402	Magnolia
0.78	16401	16401	S	86752	Magnolia sinica
0.49	10361	0	O	232378	Proteales
0.49	10361	91	F	4328	Proteaceae
0.31	6575	0	G	54954	Telopea
0.31	6575	6575	S	54955	Telopea speciosissima
0.18	3695	0	G	4329	Macadamia
0.18	3695	3695	S	60698	Macadamia integrifolia
0.08	1743	0	O	261007	Nymphaeales
0.08	1743	0	F	4410	Nymphaeaceae
0.08	1743	0	G	4418	Nymphaea
0.08	1743	1743	S	210225	Nymphaea colorata
0.05	1033	0	P6	1437180	Acrogymnospermae
0.05	1033	0	C	58019	Pinopsida
0.05	1033	0	C1	3313	Pinidae
0.05	1033	0	C2	2821351	Conifers II
0.05	1033	0	O	1446379	Cupressales
0.05	1033	0	F	3367	Cupressaceae
0.05	1033	0	G	3368	Cryptomeria
0.05	1033	1033	S	3369	Cryptomeria japonica
0.00	30	0	P3	3208	Bryophyta
0.00	30	0	P4	404260	Bryophytina
0.00	30	0	C	3214	Bryopsida
0.00	30	0	C1	114656	Funariidae
0.00	30	0	O	3215	Funariales
0.00	30	0	F	3216	Funariaceae
0.00	30	0	G	37414	Physcomitrium
0.00	30	30	S	3218	Physcomitrium patens
0.00	20	0	P	3041	Chlorophyta
0.00	15	0	C	1035538	Mamiellophyceae

0.00	15	1	O	13792	Mamiellales
0.00	14	0	F	1525212	Bathycoccaceae
0.00	14	11	G	70447	Ostreococcus
0.00	2	0	G1	2268852	unclassified Ostreococcus
0.00	2	0	S	242159	Ostreococcus sp. 'lucimarinus'
0.00	2	2	S1	436017	Ostreococcus lucimarinus CCE9901
0.00	1	1	S	70448	Ostreococcus tauri
0.00	5	0	P1	2692248	core chlorophytes
0.00	5	0	C	3166	Chlorophyceae
0.00	5	0	C1	2812636	CS clade
0.00	5	0	O	3042	Chlamydomonadales
0.00	5	0	F	3051	Chlamydomonadaceae
0.00	5	0	G	3052	Chlamydomonas
0.00	5	5	S	3055	Chlamydomonas reinhardtii
1.00	2007	61	D1	33154	Opisthokonta
0.09	1946	0	K	4751	Fungi
0.09	1923	60	K1	451864	Dikarya
0.07	1476	8	P	4890	Ascomycota
0.07	1464	68	P1	716545	saccharomyceta
0.05	1147	0	P2	147538	Pezizomycotina
0.05	1147	37	P3	716546	leotiomyceta
0.02	467	0	P4	715989	sordariomyceta
0.02	430	12	C	147550	Sordariomycetes
0.02	358	1	C1	222543	Hypocreomycetidae
0.02	323	63	O	5125	Hypocreales
0.01	218	0	F	110618	Nectriaceae
0.01	218	15	G	5506	Fusarium
0.01	136	8	G1	232080	Fusarium solani species complex
0.01	123	123	S	1328300	Fusarium keratoplasticum
0.00	5	5	S	195108	Fusarium falciforme
0.00	38	5	G1	569360	Fusarium sambucinum species complex
0.00	26	26	S	36050	Fusarium poae
0.00	6	0	S	5518	Fusarium graminearum
0.00	6	6	S1	229533	Fusarium graminearum PH-1
0.00	1	0	S	101028	Fusarium pseudograminearum
0.00	1	1	S1	1028729	Fusarium pseudograminearum CS3096
0.00	24	0	G1	171631	Fusarium oxysporum species complex
0.00	24	1	S	5507	Fusarium oxysporum

0.00	22	0	S1	59765	Fusarium oxysporum f. sp. lycopersici
0.00	22	22	S2	426428	Fusarium oxysporum f. sp. lycopersici 4287
0.00	1	1	S1	660027	Fusarium oxysporum Fo47
0.00	4	0	G1	171627	Fusarium fujikuroi species complex
0.00	3	0	S	117187	Fusarium verticillioides
0.00	3	3	S1	334819	Fusarium verticillioides 7600
0.00	1	0	S	5127	Fusarium fujikuroi
0.00	1	1	S1	1279085	Fusarium fujikuroi IMI 58289
0.00	1	1	S	1042133	Fusarium musae
0.00	19	3	F	34397	Clavicipitaceae
0.00	7	0	G	5529	Metarhizium
0.00	7	7	S	500148	Metarhizium brunneum
0.00	6	0	G	124426	Ustilaginoidea
0.00	6	6	S	1159556	Ustilaginoidea virens
0.00	3	0	G	243023	Pochonia
0.00	3	0	S	280754	Pochonia chlamydosporia
0.00	3	3	S1	1380566	Pochonia chlamydosporia 170
0.00	12	0	F	5129	Hypocreaceae
0.00	12	0	G	5543	Trichoderma
0.00	5	5	S	101201	Trichoderma asperellum
0.00	4	4	S	2034170	Trichoderma breve
0.00	3	3	S	63577	Trichoderma atroviride
0.00	6	0	F	474942	Ophiocordycipitaceae
0.00	5	0	G	1052105	Purpureocillium
0.00	5	5	S	2060973	Purpureocillium takamizusanense
0.00	1	0	G	98402	Drechmeria
0.00	1	1	S	98403	Drechmeria coniospora
0.00	5	0	F	474943	Cordycipitaceae
0.00	5	0	G	150366	Akanthomyces
0.00	5	5	S	2231603	Akanthomyces muscarius
0.00	34	0	O	1028384	Glomerellales
0.00	34	0	F	681950	Glomerellaceae
0.00	34	9	G	5455	Colletotrichum
0.00	20	0	G1	2707350	Colletotrichum destructivum species complex
0.00	11	0	S	80884	Colletotrichum higginsianum
0.00	11	11	S1	759273	Colletotrichum higginsianum IMI

```
349063
  0.00  9       9       S       34406                                   Colletotrichum destructivum
  0.00  5       0       G1      2707335                               Colletotrichum acutatum species complex
  0.00  5       5       S       145971                                  Colletotrichum lupini
  0.00  60      0       C1      222544                        Sordariomycetidae
  0.00  53      4       O       5139                            Sordariales
  0.00  21      1       F       5148                              Sordariaceae
  0.00  15      0       G       5140                                Neurospora
  0.00  15      0       S       5141                                  Neurospora crassa
  0.00  15      15      S1      367110                                  Neurospora crassa OR74A
  0.00  5       0       G       5146                                Sordaria
  0.00  5       5       S       5147                                  Sordaria macrospora
  0.00  20      0       F       2609812                           Podosporaceae
  0.00  20      10      G       5144                                Podospora
  0.00  5       5       S       2093780                               Podospora pseudopauciseta
  0.00  4       4       S       2093779                               Podospora pseudocomata
  0.00  1       1       S       2093777                               Podospora bellae-mahoneyi
  0.00  8       0       F       35718                             Chaetomiaceae
  0.00  8       0       G       1920207                             Thermothelomyces
  0.00  8       0       S       78579                                 Thermothelomyces thermophilus
  0.00  8       8       S1      573729                                  Thermothelomyces thermophilus ATCC
42464
  0.00  7       0       O       639021                          Magnaporthales
  0.00  7       0       F       2528436                           Pyriculariaceae
  0.00  7       0       G       48558                               Pyricularia
  0.00  3       3       S       1578925                               Pyricularia pennisetigena
  0.00  2       2       S       148305                                Pyricularia grisea
  0.00  2       0       S       318829                                Pyricularia oryzae
  0.00  2       2       S1      242507                                  Pyricularia oryzae 70-15
  0.00  37      0       C       147548                      Leotiomycetes
  0.00  37      0       O       5178                          Helotiales
  0.00  37      0       F       28983                           Sclerotiniaceae
  0.00  37      0       G       33196                             Botrytis
  0.00  37      0       S       40559                               Botrytis cinerea
  0.00  37      37      S1      332648                                Botrytis cinerea B05.10
  0.02  443     0       P4      715962                    dothideomyceta
  0.02  443     0       C       147541                      Dothideomycetes
  0.02  411     0       C1      451868                        Pleosporomycetidae
```

0.02	411	0	O	92860	Pleosporales
0.02	411	14	O1	715340	Pleosporineae
0.01	283	0	F	28556	Pleosporaceae
0.01	283	0	G	5027	Pyrenophora
0.01	283	283	S	45151	Pyrenophora tritici-repentis
0.01	114	0	F	683158	Didymellaceae
0.01	114	0	G	5453	Ascochyta
0.01	114	114	S	5454	Ascochyta rabiei
0.00	32	0	C1	451867	Dothideomycetidae
0.00	32	0	O	2726947	Mycosphaerellales
0.00	32	4	F	93133	Mycosphaerellaceae
0.00	13	0	G	1047167	Zymoseptoria
0.00	13	0	S	1047171	Zymoseptoria tritici
0.00	13	13	S1	336722	Zymoseptoria tritici IPO323
0.00	8	0	G	29002	Cercospora
0.00	8	8	S	122368	Cercospora beticola
0.00	7	0	G	2897311	Fulvia
0.00	7	7	S	5499	Fulvia fulva
0.01	200	0	C	147545	Eurotiomycetes
0.01	200	10	C1	451871	Eurotiomycetidae
0.01	118	16	O	5042	Eurotiales
0.00	82	3	F	1131492	Aspergillaceae
0.00	67	4	G	5052	Aspergillus
0.00	49	22	G1	2720871	Aspergillus subgen. Circumdati
0.00	15	15	S	1069201	Aspergillus luchuensis
0.00	12	0	S	5062	Aspergillus oryzae
0.00	12	12	S1	510516	Aspergillus oryzae RIB40
0.00	6	6	S	1220207	Aspergillus puulaauensis
0.00	4	0	G1	2720874	Aspergillus subgen. Aspergillus
0.00	4	4	S	182096	Aspergillus chevalieri
0.00	3	0	G1	2720870	Aspergillus subgen. Nidulantes
0.00	3	0	S	162425	Aspergillus nidulans
0.00	3	3	S1	227321	Aspergillus nidulans FGSC A4
0.00	1	0	G1	2720872	Aspergillus subgen. Fumigati
0.00	1	0	S	746128	Aspergillus fumigatus
0.00	1	1	S1	330879	Aspergillus fumigatus Af293
0.00	12	0	G	5073	Penicillium
0.00	9	9	S	69781	Penicillium oxalicum

0.00	3	3	S	36651	Penicillium digitatum
0.00	20	0	F	28568	Trichocomaceae
0.00	20	9	G	5094	Talaromyces
0.00	7	0	G1	2752542	Talaromyces sect. Islandici
0.00	7	7	S	121627	Talaromyces rugulosus
0.00	4	0	G1	2752537	Talaromyces sect. Talaromyces
0.00	4	4	S	37727	Talaromyces marneffei
0.00	72	0	O	33183	Onygenales
0.00	72	0	F	33184	Onygenaceae
0.00	72	0	G	5500	Coccidioides
0.00	72	0	S	199306	Coccidioides posadasii
0.00	72	72	S1	443226	Coccidioides posadasii str. Silveira
0.01	249	15	P2	147537	Saccharomycotina
0.01	107	3	C	3239874	Pichiomycetes
0.00	67	0	O	3243775	Pichiales
0.00	67	0	F	1156497	Pichiaceae
0.00	44	2	G	13366	Brettanomyces
0.00	35	35	S	5007	Brettanomyces bruxellensis
0.00	7	7	S	13502	Brettanomyces nanus
0.00	23	0	G	4919	Pichia
0.00	23	23	S	4909	Pichia kudriavzevii
0.00	37	1	O	2916678	Serinales
0.00	20	1	F	27319	Metschnikowiaceae
0.00	17	0	G	3303203	Candidozyma
0.00	17	17	S	498019	Candidozyma auris
0.00	2	0	G	3300567	Australozyma
0.00	2	2	S	291208	Australozyma saopauloensis
0.00	16	3	F	766764	Debaryomycetaceae
0.00	10	7	F1	1535325	Candida/Lodderomyces clade
0.00	3	0	G	36913	Lodderomyces
0.00	2	2	S	36914	Lodderomyces elongisporus
0.00	1	1	S	1775926	Lodderomyces beijingensis
0.00	2	0	G	766733	Scheffersomyces
0.00	2	0	S	4924	Scheffersomyces stipitis
0.00	2	2	S1	322104	Scheffersomyces stipitis CBS 6054
0.00	1	0	G	766765	Yamadazyma
0.00	1	1	S	2315449	Yamadazyma tenuis
0.00	88	0	C	3239873	Dipodascomycetes

0.00	88	0	O	3243772	Dipodascales
0.00	85	0	O1	3316682	Dipodascales incertae sedis
0.00	85	0	G	4951	Yarrowia
0.00	85	85	S	4952	Yarrowia lipolytica
0.00	3	0	F	410830	Trichomonascaceae
0.00	3	0	G	410829	Sugiyamaella
0.00	3	3	S	796027	Sugiyamaella lignohabitans
0.00	39	0	C	4891	Saccharomycetes
0.00	38	0	O	4892	Saccharomycetales
0.00	38	15	F	4893	Saccharomycetaceae
0.00	5	0	G	4930	Saccharomyces
0.00	4	0	S	4932	Saccharomyces cerevisiae
0.00	4	4	S1	559292	Saccharomyces cerevisiae S288C
0.00	1	1	S	1080349	Saccharomyces eubayanus
0.00	5	0	G	3163253	Henningerozyma
0.00	5	0	S	1071379	Henningerozyma blattae
0.00	5	5	S1	1071380	Henningerozyma blattae CBS 6284
0.00	4	4	G	33170	Eremothecium
0.00	2	0	G	113604	Tetrapisispora
0.00	2	0	S	113608	Tetrapisispora phaffii
0.00	2	2	S1	1071381	Tetrapisispora phaffii CBS 4417
0.00	2	0	G	278028	Naumovozyma
0.00	2	0	S	27289	Naumovozyma dairenensis
0.00	2	2	S1	1071378	Naumovozyma dairenensis CBS 421
0.00	2	0	G	3163251	Huiozyma
0.00	2	0	S	588726	Huiozyma naganishii
0.00	2	2	S1	1071383	Huiozyma naganishii CBS 8797
0.00	1	0	G	4948	Torulaspora
0.00	1	1	S	4950	Torulaspora delbrueckii
0.00	1	0	G	4953	Zygosaccharomyces
0.00	1	1	S	4956	Zygosaccharomyces rouxii
0.00	1	0	G	1196389	Zygotorulaspora
0.00	1	1	S	42260	Zygotorulaspora mrakii
0.00	1	0	O	3243779	Saccharomycodales
0.00	1	0	F	34365	Saccharomycodaceae
0.00	1	0	G	36034	Saccharomycodes
0.00	1	1	S	36035	Saccharomycodes ludwigii
0.00	4	0	P1	451866	Taphrinomycotina

0.00	4	0	C	147554	Schizosaccharomycetes
0.00	4	0	O	34346	Schizosaccharomycetales
0.00	4	0	F	4894	Schizosaccharomycetaceae
0.00	4	0	G	4895	Schizosaccharomyces
0.00	2	2	S	4896	Schizosaccharomyces pombe
0.00	2	2	S	2545709	Schizosaccharomyces osmophilus
0.02	387	5	P	5204	Basidiomycota
0.01	195	1	P1	5302	Agaricomycotina
0.01	167	1	C	155616	Tremellomycetes
0.01	127	0	O	5234	Tremellales
0.01	127	10	F	1884633	Cryptococcaceae
0.00	105	18	G	490731	Kwoniella
0.00	22	0	S	463800	Kwoniella mangrovensis
0.00	22	22	S1	1296122	Kwoniella mangroviensis CBS 8507
0.00	19	0	S	324769	Kwoniella bestiolae
0.00	19	19	S1	1296100	Kwoniella bestiolae CBS 10118
0.00	13	13	S	1268659	Kwoniella botswanensis
0.00	13	13	S	1734106	Kwoniella shandongensis
0.00	8	0	S	324770	Kwoniella dejecticola
0.00	8	8	S1	1296121	Kwoniella dejecticola CBS 10117
0.00	5	5	S	1651941	Kwoniella newhampshirensis
0.00	4	0	S	4975	Kwoniella dendrophila
0.00	4	4	S1	1295534	Kwoniella dendrophila CBS 6074
0.00	2	0	S	1247712	Kwoniella europaea
0.00	2	2	S1	1423913	Kwoniella europaea PYCC6329
0.00	1	1	S	564305	Kwoniella shivajii
0.00	12	4	G	5206	Cryptococcus
0.00	6	0	G1	1884637	Cryptococcus gattii species complex
0.00	3	0	S	1859096	Cryptococcus deuterogattii
0.00	3	3	S1	294750	Cryptococcus deuterogattii R265
0.00	2	2	S	1859122	Cryptococcus decagattii
0.00	1	0	S	37769	Cryptococcus gattii
0.00	1	1	S1	367775	Cryptococcus gattii WM276
0.00	1	0	S	5208	Cryptococcus depauperatus
0.00	1	1	S1	1295531	Cryptococcus depauperatus CBS 7841
0.00	1	0	G1	1897064	Cryptococcus neoformans species complex
0.00	1	0	S	5207	Cryptococcus neoformans
0.00	1	0	S1	178876	Cryptococcus neoformans var. grubii

0.00	1	1	S2	235443	Cryptococcus neoformans var. grubii H99
0.00	39	0	O	1851469	Trichosporonales
0.00	39	4	F	1759442	Trichosporonaceae
0.00	19	0	G	1851468	Vanrija
0.00	19	19	S	143232	Vanrija pseudolonga
0.00	16	0	G	1838142	Cutaneotrichosporon
0.00	16	16	S	279322	Cutaneotrichosporon cavernicola
0.00	27	2	C	155619	Agaricomycetes
0.00	13	0	C1	452333	Agaricomycetidae
0.00	13	1	O	5338	Agaricales
0.00	6	0	O1	2982305	Agaricineae
0.00	6	0	F	40562	Strophariaceae
0.00	6	0	G	71950	Psilocybe
0.00	6	6	S	181762	Psilocybe cubensis
0.00	6	0	O1	2982316	Marasmiineae
0.00	6	0	F	654128	Marasmiaceae
0.00	6	0	G	34448	Marasmius
0.00	6	6	S	181124	Marasmius oreades
0.00	12	0	C1	355688	Agaricomycetes incertae sedis
0.00	12	0	O	36064	Cantharellales
0.00	12	0	F	5250	Ceratobasidiaceae
0.00	12	0	G	1322061	Rhizoctonia
0.00	12	12	S	456999	Rhizoctonia solani
0.01	160	0	P1	29000	Pucciniomycotina
0.01	160	0	C	162484	Pucciniomycetes
0.01	160	0	O	5258	Pucciniales
0.01	160	0	F	5262	Pucciniaceae
0.01	160	55	G	5296	Puccinia
0.00	78	0	S	27350	Puccinia striiformis
0.00	78	78	S1	168172	Puccinia striiformis f. sp. tritici
0.00	27	27	S	208348	Puccinia triticina
0.00	27	2	P1	452284	Ustilaginomycotina
0.00	19	0	C	1538075	Malasseziomycetes
0.00	19	0	O	162474	Malasseziales
0.00	19	0	F	742845	Malasseziaceae
0.00	19	3	G	55193	Malassezia
0.00	14	14	S	76775	Malassezia restricta
0.00	1	1	S	223818	Malassezia japonica

0.00	1	1	S	2020962	Malassezia vespertilionis
0.00	6	0	C	5257	Ustilaginomycetes
0.00	6	0	O	5267	Ustilaginales
0.00	6	0	F	5268	Ustilaginaceae
0.00	6	0	G	63265	Sporisorium
0.00	6	6	S	280036	Sporisorium graminicola
0.00	23	0	K1	112252	Fungi incertae sedis
0.00	21	0	P	1913637	Mucoromycota
0.00	21	0	P1	214504	Glomeromycotina
0.00	21	0	C	214506	Glomeromycetes
0.00	21	0	O	36750	Glomerales
0.00	21	0	F	36751	Glomeraceae
0.00	21	0	G	1129544	Rhizophagus
0.00	21	21	S	588596	Rhizophagus irregularis
0.00	2	0	P	6029	Microsporidia
0.00	2	0	P1	6032	Apansporoblastina
0.00	1	0	F	27974	Nosematidae
0.00	1	0	G	6038	Vairimorpha
0.00	1	1	S	6039	Vairimorpha necatrix
0.00	1	0	F	36734	Unikaryonidae
0.00	1	0	G	6033	Encephalitozoon
0.00	1	0	S	27973	Encephalitozoon hellem
0.00	1	1	S1	907965	Encephalitozoon hellem ATCC 50504
0.01	277	7	D1	2698737	Sar
0.01	135	0	D2	33630	Alveolata
0.01	135	4	P	5794	Apicomplexa
0.00	101	0	C	422676	Aconoidasida
0.00	98	0	O	5819	Haemosporida
0.00	98	0	F	1639119	Plasmodiidae
0.00	98	58	G	5820	Plasmodium
0.00	19	14	G1	418103	Plasmodium (Plasmodium)
0.00	5	5	S	5855	Plasmodium vivax
0.00	10	2	G1	418101	Plasmodium (Vinckeia)
0.00	4	0	S	5821	Plasmodium berghei
0.00	4	4	S1	5823	Plasmodium berghei ANKA
0.00	3	0	S	5860	Plasmodium vinckei
0.00	3	3	S1	54757	Plasmodium vinckei vinckei
0.00	1	1	S	5861	Plasmodium yoelii

0.00	6	1	G1	418107	Plasmodium (Laverania)
0.00	3	3	S	5854	Plasmodium reichenowi
0.00	2	2	S	880535	Plasmodium sp. gorilla clade G2
0.00	4	0	G1	418104	Plasmodium (Haemamoeba)
0.00	4	4	S	85471	Plasmodium relictum
0.00	1	1	S	208452	Plasmodium coatneyi
0.00	3	0	O	5863	Piroplasmida
0.00	2	0	F	32594	Babesiidae
0.00	2	0	G	5864	Babesia
0.00	1	0	S	5868	Babesia microti
0.00	1	1	S1	1133968	Babesia microti strain RI
0.00	1	1	S	323732	Babesia duncani
0.00	1	0	F	27994	Theileriidae
0.00	1	1	G	5873	Theileria
0.00	30	0	C	1280412	Conoidasida
0.00	30	0	C1	5796	Coccidia
0.00	30	0	O	75739	Eucoccidiorida
0.00	30	1	O1	423054	Eimeriorina
0.00	27	7	F	5809	Sarcocystidae
0.00	20	0	G	5810	Toxoplasma
0.00	20	0	S	5811	Toxoplasma gondii
0.00	20	20	S1	508771	Toxoplasma gondii ME49
0.00	2	0	F	35082	Cryptosporidiidae
0.00	2	0	G	5806	Cryptosporidium
0.00	2	0	S	5807	Cryptosporidium parvum
0.00	2	2	S1	353152	Cryptosporidium parvum Iowa II
0.01	134	1	D2	33634	Stramenopiles
0.00	69	0	D3	2696291	Ochrophyta
0.00	69	5	P	2836	Bacillariophyta
0.00	37	0	C	33849	Bacillariophyceae
0.00	37	0	C1	33850	Bacillariophycidae
0.00	37	0	O	38748	Naviculales
0.00	37	0	F	38749	Phaeodactylaceae
0.00	37	0	G	2849	Phaeodactylum
0.00	37	0	S	2850	Phaeodactylum tricornutum
0.00	37	37	S1	556484	Phaeodactylum tricornutum CCAP 1055/1
0.00	27	0	C	33836	Coscinodiscophyceae
0.00	27	0	C1	33846	Thalassiosirophycidae

0.00	27	0	O	33847	Thalassiosirales
0.00	27	0	F	29202	Thalassiosiraceae
0.00	27	0	G	35127	Thalassiosira
0.00	27	0	S	35128	Thalassiosira pseudonana
0.00	27	27	S1	296543	Thalassiosira pseudonana CCMP1335
0.00	64	0	P	4762	Oomycota
0.00	64	0	O	4776	Peronosporales
0.00	64	0	F	4777	Peronosporaceae
0.00	64	0	G	4778	Bremia
0.00	64	64	S	4779	Bremia lactucae
0.00	1	0	D2	543769	Rhizaria
0.00	1	0	P	136419	Cercozoa
0.00	1	0	C	29197	Chlorarachniophyceae
0.00	1	0	G	227085	Bigelowiella
0.00	1	1	S	227086	Bigelowiella natans
0.00	15	1	C	3027	Cryptophyceae
0.00	8	0	O	589342	Pyrenomonadales
0.00	8	0	F	589343	Geminigeraceae
0.00	8	0	G	55528	Guillardia
0.00	8	8	S	55529	Guillardia theta
0.00	6	0	O	589350	Cryptomonadales
0.00	5	0	F	589351	Hemiselmidaceae
0.00	5	0	G	77924	Hemiselmis
0.00	5	5	S	464988	Hemiselmis andersenii
0.00	1	0	F	2896	Cryptomonadaceae
0.00	1	0	G	3030	Cryptomonas
0.00	1	1	S	2898	Cryptomonas paramecium
0.00	10	0	D1	554915	Amoebozoa
0.00	10	0	P	2605435	Evosea
0.00	10	0	C	142796	Eumycetozoa
0.00	10	0	C1	33083	Dictyostelia
0.00	10	0	O	2058949	Dictyosteliales
0.00	10	0	F	2058185	Dictyosteliaceae
0.00	10	0	G	5782	Dictyostelium
0.00	10	0	S	44689	Dictyostelium discoideum
0.00	10	10	S1	352472	Dictyostelium discoideum AX4
0.00	10	0	D1	2611352	Discoba
0.00	10	0	P	33682	Euglenozoa

0.00	10	0	C	5653	Kinetoplastea
0.00	10	0	C1	2704647	Metakinetoplastina
0.00	10	0	O	2704949	Trypanosomatida
0.00	10	0	F	5654	Trypanosomatidae
0.00	8	2	F1	1286322	Leishmaniinae
0.00	4	0	G	5658	Leishmania
0.00	3	1	G1	37616	Viannia
0.00	2	0	G2	38579	Leishmania guyanensis species complex
0.00	2	2	S	5679	Leishmania panamensis
0.00	1	0	G1	38568	Leishmania
0.00	1	0	G2	38574	Leishmania donovani species complex
0.00	1	1	S	5661	Leishmania donovani
0.00	2	0	G	2761499	Porcisia
0.00	2	2	S	2761500	Porcisia hertigi
0.00	2	0	G	5690	Trypanosoma
0.00	2	0	G1	39700	Trypanozoon
0.00	2	1	S	5691	Trypanosoma brucei
0.00	1	0	S1	31285	Trypanosoma brucei gambiense
0.00	1	1	S2	679716	Trypanosoma brucei gambiense DAL972
0.00	9	0	D1	2611341	Metamonada
0.00	5	0	P	207245	Fornicata
0.00	5	0	O	5738	Diplomonadida
0.00	5	0	F	5739	Hexamitidae
0.00	3	0	F1	68459	Giardiinae
0.00	3	0	G	5740	Giardia
0.00	3	3	S	5741	Giardia intestinalis
0.00	2	0	F1	68460	Hexamitinae
0.00	2	0	G	39709	Spironucleus
0.00	2	2	S	348837	Spironucleus salmonicida
0.00	4	0	P	5719	Parabasalia
0.00	4	0	O	37104	Trichomonadida
0.00	4	0	F	181550	Trichomonadidae
0.00	4	0	G	5721	Trichomonas
0.00	4	0	S	5722	Trichomonas vaginalis
0.00	4	4	S1	412133	Trichomonas vaginalis G3
0.00	4	0	P	2763	Rhodophyta
0.00	4	0	C	2797	Bangiophyceae
0.00	4	0	O	265318	Cyanidiales

```
0.00  4       0       F       265316              Cyanidiaceae
0.00  4       0       G       45156                 Cyanidioschyzon
0.00  4       0       S       45157                   Cyanidioschyzon merolae
0.00  4       4       S1      280699                    Cyanidioschyzon merolae strain 10D
0.16  3461    399             2               Bacteria
0.07  1436    36      P       1224              Pseudomonadota
0.05  1074    95      C       1236                Gammaproteobacteria
0.03  582     34      O       91347                 Enterobacterales
0.02  435     8       F       1903409                 Erwiniaceae
0.02  379     187     G       551                       Erwinia
0.01  177     177     S       68334                       Erwinia aphidicola
0.00  5       5       S       79967                       Erwinia pyrifoliae
0.00  3       3       S       552                         Erwinia amylovora
0.00  3       3       S       65700                       Erwinia tracheiphila
0.00  2       2       S       1922217                     Candidatus Erwinia haradaeae
0.00  2       0       G1      2622719                     unclassified Erwinia
0.00  2       2       S       2675378                       Erwinia sp. E602
0.00  32      25      G       53335                     Pantoea
0.00  5       0       G1      1654067                     Pantoea agglomerans group
0.00  5       5       S       549                           Pantoea agglomerans
0.00  1       1       G1      2630326                     unclassified Pantoea
0.00  1       1       S       470931                      Pantoea anthophila
0.00  16      0       G       32199                     Buchnera
0.00  16      5       S       9                           Buchnera aphidicola
0.00  3       3       S1      98802                         Buchnera aphidicola (Panaphis juglandis)
0.00  2       2       S1      2994483                       Buchnera aphidicola (Hyalopterus amygdali)
0.00  2       2       S1      98797                         Buchnera aphidicola (Thelaxes suberi)
0.00  1       1       S1      911343                        Buchnera aphidicola (Brevicoryne brassicae)
0.00  1       1       S1      1258543                       Buchnera aphidicola (Lipaphis pseudobrassicae)
0.00  1       1       S1      2994490                       Buchnera aphidicola (Symydobius americanus)
0.00  1       1       S1      655391                        Buchnera aphidicola (Nippolachnus piri)
0.00  75      16      F       543                     Enterobacteriaceae
0.00  17      0       G       561                       Escherichia
0.00  17      17      S       562                         Escherichia coli
0.00  17      0       F1      2890311                   Klebsiella/Raoultella group
0.00  17      1       G       570                         Klebsiella
0.00  11      10      S       573                           Klebsiella pneumoniae
0.00  1       1       S1      72407                           Klebsiella pneumoniae subsp. pneumoniae
```

0.00	1	1	S	548	Klebsiella aerogenes
0.00	1	1	S	244366	Klebsiella variicola
0.00	1	0	S	1463165	Klebsiella quasipneumoniae
0.00	1	1	S1	1667327	Klebsiella quasipneumoniae subsp. quasipneumoniae
0.00	1	1	S	2058152	Klebsiella grimontii
0.00	1	1	S	2489010	Klebsiella africana
0.00	7	0	G	590	Salmonella
0.00	7	0	S	28901	Salmonella enterica
0.00	7	0	S1	59201	Salmonella enterica subsp. enterica
0.00	2	2	S2	90371	Salmonella enterica subsp. enterica serovar Typhimurium
0.00	1	1	S2	115981	Salmonella enterica subsp. enterica serovar Montevideo
0.00	1	0	S2	149539	Salmonella enterica subsp. enterica serovar Enteritidis
0.00	1	1	S3	1243620	Salmonella enterica subsp. enterica serovar Enteritidis str. EC20110355
0.00	1	0	S2	58096	Salmonella enterica subsp. enterica serovar Bareilly
0.00	1	1	S3	1182172	Salmonella enterica subsp. enterica serovar Bareilly str. CFSAN000661
0.00	1	1	S2	28144	Salmonella enterica subsp. enterica serovar Derby
0.00	1	1	S2	2583588	Salmonella enterica subsp. enterica serovar 1,4,[5],12:i:-
0.00	5	0	F1	191675	Enterobacteriaceae incertae sedis
0.00	5	0	F2	84563	ant, tsetse, mealybug, aphid, etc. endosymbionts
0.00	3	0	F3	84564	ant endosymbionts
0.00	2	0	G	203804	Candidatus Blochmanniella
0.00	2	0	G1	711328	unclassified Candidatus Blochmanniella
0.00	2	2	S	2945587	Blochmannia endosymbiont of Camponotus modoc
0.00	1	0	G	1699619	Candidatus Westeberhardia
0.00	1	1	S	1594731	Candidatus Westeberhardia cardiocondylae
0.00	1	0	G	801	Symbiopectobacterium
0.00	1	1	S	2871826	Symbiopectobacterium purcellii
0.00	1	0	G	1906659	Candidatus Hoaglandella
0.00	1	1	S	1778263	Candidatus Hoaglandella endobia
0.00	3	2	G	547	Enterobacter
0.00	1	0	G1	354276	Enterobacter cloacae complex
0.00	1	1	S	158836	Enterobacter hormaechei

0.00	3	0	F1	36866	unclassified Enterobacteriaceae
0.00	2	2	S	2675792	Enterobacteriaceae endosymbiont of Neohaemonia nigricornis
0.00	1	1	S	2675797	Enterobacteriaceae endosymbiont of Plateumaris sericea
0.00	2	0	G	544	Citrobacter
0.00	1	0	G1	1344959	Citrobacter freundii complex
0.00	1	1	S	546	Citrobacter freundii
0.00	1	1	S	2971264	Citrobacter enshiensis
0.00	1	0	G	1330546	Pluralibacter
0.00	1	1	S	1334193	[Enterobacter] lignolyticus
0.00	1	0	G	413496	Cronobacter
0.00	1	1	S	28141	Cronobacter sakazakii
0.00	1	0	G	401618	Candidatus Riesia
0.00	1	1	S	428411	Candidatus Riesia pediculischaeffi
0.00	1	0	G	158483	Cedecea
0.00	1	0	G1	2649846	unclassified Cedecea
0.00	1	1	S	2545798	Cedecea sp. FDAARGOS_727
0.00	1	0	G	2726810	Scandinavium
0.00	1	1	S	1851514	Scandinavium goeteborgense
0.00	21	0	F	1903414	Morganellaceae
0.00	10	0	G	581	Morganella
0.00	10	10	S	582	Morganella morganii
0.00	5	2	G	586	Providencia
0.00	3	3	S	587	Providencia rettgeri
0.00	3	0	G	583	Proteus
0.00	2	2	S	584	Proteus mirabilis
0.00	1	1	S	2050967	Proteus faecis
0.00	2	0	G	626	Xenorhabdus
0.00	1	1	S	628	Xenorhabdus nematophila
0.00	1	1	S	351672	Xenorhabdus griffiniae
0.00	1	0	G	637	Arsenophonus
0.00	1	0	G1	2627083	unclassified Arsenophonus
0.00	1	1	S	1231049	Arsenophonus endosymbiont of Aphis craccivora
0.00	8	2	F	1903411	Yersiniaceae
0.00	3	0	G	613	Serratia
0.00	1	0	G1	2647522	unclassified Serratia (in: enterobacteria)
0.00	1	1	S	1327989	Serratia sp. FS14
0.00	1	0	S	82996	Serratia plymuthica

0.00	1	1	S1	1006598	Serratia plymuthica RVH1
0.00	1	1	S	138074	Serratia symbiotica
0.00	3	0	G	629	Yersinia
0.00	2	0	G1	1649845	Yersinia pseudotuberculosis complex
0.00	2	0	S	632	Yersinia pestis
0.00	2	2	S1	1345702	Yersinia pestis 2944
0.00	1	1	S	33060	Yersinia mollaretii
0.00	7	0	F	1903410	Pectobacteriaceae
0.00	4	1	G	204037	Dickeya
0.00	2	2	S	2259638	Dickeya lacustris
0.00	1	1	S	204042	Dickeya zeae
0.00	2	0	G	122277	Pectobacterium
0.00	1	1	S	1507808	Pectobacterium actinidiae
0.00	1	1	S	2485124	Pectobacterium polonicum
0.00	1	0	G	2884243	Musicola
0.00	1	1	S	69223	Musicola paradisiaca
0.00	2	0	F	2812006	Bruguierivoracaceae
0.00	2	0	G	84565	Sodalis
0.00	1	1	S	1239307	Sodalis praecaptivus
0.00	1	1	S	2697027	Sodalis ligni
0.01	178	0	O	2887326	Moraxellales
0.01	178	1	F	468	Moraxellaceae
0.01	158	103	G	469	Acinetobacter
0.00	32	0	G1	909768	Acinetobacter calcoaceticus/baumannii complex
0.00	29	28	S	470	Acinetobacter baumannii
0.00	1	1	S1	1279013	Acinetobacter baumannii PR07
0.00	2	2	S	48296	Acinetobacter pittii
0.00	1	1	S	471	Acinetobacter calcoaceticus
0.00	7	3	G1	196816	unclassified Acinetobacter
0.00	2	2	S	3045147	Acinetobacter sp. KCTC 92772
0.00	2	2	S	2919376	Acinetobacter sp. AOR07_HL
0.00	7	7	S	40215	Acinetobacter junii
0.00	3	3	S	29430	Acinetobacter haemolyticus
0.00	2	0	S	52133	Acinetobacter venetianus
0.00	2	2	S1	1197884	Acinetobacter venetianus VE-C3
0.00	2	2	S	28090	Acinetobacter lwoffii
0.00	1	1	S	108980	Acinetobacter ursingii
0.00	1	1	S	756892	Acinetobacter indicus

0.00	14	11	G	475	Moraxella
0.00	2	2	S	90241	Moraxella lincolnii
0.00	1	1	S	34062	Moraxella osloensis
0.00	4	0	G	497	Psychrobacter
0.00	4	2	G1	196806	unclassified Psychrobacter
0.00	1	1	S	2517899	Psychrobacter sp. KH172YL61
0.00	1	1	S	1415574	Psychrobacter sp. LV10R520-6
0.00	1	0	G	2824158	Aquirhabdus
0.00	1	1	S	2283318	Aquirhabdus parva
0.01	106	1	O	72274	Pseudomonadales
0.00	105	12	F	135621	Pseudomonadaceae
0.00	89	33	G	286	Pseudomonas
0.00	22	0	G1	136841	Pseudomonas aeruginosa group
0.00	21	13	S	287	Pseudomonas aeruginosa
0.00	4	4	S1	1408276	Pseudomonas aeruginosa LESlike5
0.00	3	3	S1	381754	Pseudomonas aeruginosa PA7
0.00	1	1	S1	1408272	Pseudomonas aeruginosa LES431
0.00	1	1	S	53406	Pseudomonas anguilliseptica
0.00	12	5	G1	196821	unclassified Pseudomonas
0.00	2	2	S	2954099	Pseudomonas sp. FP818
0.00	1	1	S	3043447	Pseudomonas sp. P9_2
0.00	1	1	S	2954080	Pseudomonas sp. FP1762
0.00	1	1	S	2954078	Pseudomonas sp. FP1740
0.00	1	1	S	1206777	Pseudomonas sp. Lz4W
0.00	1	1	S	3019968	Pseudomonas sp. MM227
0.00	5	5	S	2954101	Pseudomonas beijingensis
0.00	5	0	G1	136843	Pseudomonas fluorescens group
0.00	4	4	S	183795	Pseudomonas mediterranea
0.00	1	0	S	294	Pseudomonas fluorescens
0.00	1	1	S1	1221522	Pseudomonas fluorescens NCIMB 11764
0.00	4	0	G1	136845	Pseudomonas putida group
0.00	2	2	S	47880	Pseudomonas fulva
0.00	1	1	S	303	Pseudomonas putida
0.00	1	1	S	70775	Pseudomonas plecoglossicida
0.00	1	1	S	1495066	Pseudomonas capeferrum
0.00	1	1	S	226910	Pseudomonas batumici
0.00	1	1	S	1605838	Pseudomonas coleopterorum
0.00	1	1	S	1302376	Candidatus Pseudomonas adelgestsugas

0.00	1	0	G1	136849	Pseudomonas syringae group
0.00	1	0	S	251701	Pseudomonas syringae group genomosp. 3
0.00	1	1	S1	323	Pseudomonas syringae pv. tomato
0.00	1	0	G1	136842	Pseudomonas chlororaphis group
0.00	1	0	S	587753	Pseudomonas chlororaphis
0.00	1	0	S1	587851	Pseudomonas chlororaphis subsp. aureofaciens
0.00	1	1	S2	1038921	Pseudomonas chlororaphis subsp. aureofaciens 30-84
0.00	1	1	S	2666183	Pseudomonas juntendi
0.00	1	1	S	2320867	Pseudomonas cavernae
0.00	2	0	F1	351	Azotobacter group
0.00	2	2	G	352	Azotobacter
0.00	1	0	G	1649479	Permianibacter
0.00	1	1	S	1510150	Permianibacter aggregans
0.00	1	0	G	2901164	Stutzerimonas
0.00	1	0	G1	136846	Stutzerimonas stutzeri group
0.00	1	0	G2	578833	Stutzerimonas stutzeri subgroup
0.00	1	1	S	316	Stutzerimonas stutzeri
0.00	25	1	O	135614	Lysobacterales
0.00	17	0	F	32033	Lysobacteraceae
0.00	10	0	G	40323	Stenotrophomonas
0.00	10	0	G1	995085	Stenotrophomonas maltophilia group
0.00	10	10	S	40324	Stenotrophomonas maltophilia
0.00	4	0	G	338	Xanthomonas
0.00	3	0	S	56454	Xanthomonas hortorum
0.00	3	0	S1	487904	Xanthomonas hortorum pv. carotae
0.00	3	3	S2	863365	Xanthomonas hortorum pv. carotae str. M081
0.00	1	1	S	29447	Xanthomonas albilineans
0.00	2	1	G	68	Lysobacter
0.00	1	1	S	435897	Lysobacter capsici
0.00	1	0	G	83614	Luteimonas
0.00	1	0	G1	2629088	unclassified Luteimonas
0.00	1	1	S	2508168	Luteimonas sp. YGD11-2
0.00	7	0	F	1775411	Rhodanobacteraceae
0.00	7	0	G	242605	Luteibacter
0.00	7	7	S	2911537	Luteibacter aegosomatis
0.00	19	0	O	135622	Alteromonadales
0.00	11	0	F	267890	Shewanellaceae
0.00	11	2	G	22	Shewanella

0.00	2	2	S	24	Shewanella putrefaciens
0.00	2	0	G1	196818	unclassified Shewanella
0.00	1	1	S	2589987	Shewanella sp. Scap07
0.00	1	1	S	2590015	Shewanella sp. SNU WT4
0.00	2	2	S	2864203	Shewanella aegiceratis
0.00	1	1	S	38313	Shewanella algae
0.00	1	1	S	1738770	Shewanella inventionis
0.00	1	1	S	62322	Shewanella baltica
0.00	4	0	F	267888	Pseudoalteromonadaceae
0.00	4	2	G	53246	Pseudoalteromonas
0.00	1	1	S	2518973	Pseudoalteromonas rhizosphaerae
0.00	1	1	S	339617	Pseudoalteromonas viridis
0.00	3	0	F	72275	Alteromonadaceae
0.00	2	0	F1	2903219	Alteromonas/Salinimonas group
0.00	2	0	G	226	Alteromonas
0.00	1	0	S	28108	Alteromonas macleodii
0.00	1	1	S1	529120	Alteromonas macleodii ATCC 27126
0.00	1	1	S	314275	Alteromonas mediterranea
0.00	1	0	G	1621534	Paraglaciecola
0.00	1	0	G1	2685791	unclassified Paraglaciecola
0.00	1	1	S	2686358	Paraglaciecola sp. L3A3
0.00	1	0	F	267889	Colwelliaceae
0.00	1	0	G	1518149	Thalassotalea
0.00	1	1	S	3030492	Thalassotalea hakodatensis
0.00	16	0	O	135623	Vibrionales
0.00	16	0	F	641	Vibrionaceae
0.00	13	0	G	662	Vibrio
0.00	5	0	G1	717610	Vibrio harveyi group
0.00	4	3	S	670	Vibrio parahaemolyticus
0.00	1	1	S1	223926	Vibrio parahaemolyticus RIMD 2210633
0.00	1	0	G2	2315253	Vibrio diabolicus subgroup
0.00	1	1	S	50719	Vibrio diabolicus
0.00	3	0	G1	2614977	unclassified Vibrio
0.00	1	1	S	2942997	Vibrio sp. J383
0.00	1	1	S	2822843	Vibrio sp. SCSIO 43186
0.00	1	1	S	2163016	Vibrio sp. dhg
0.00	2	2	S	676	Vibrio fluvialis
0.00	1	1	S	1481923	Vibrio astriarenae

0.00	1	1	S	2587862	Vibrio aquimaris
0.00	1	1	S	2572923	Vibrio taketomensis
0.00	3	0	G	657	Photobacterium
0.00	2	0	S	553611	Photobacterium leiognathi
0.00	2	2	S1	48408	Photobacterium leiognathi subsp. mandapamensis
0.00	1	0	S	38293	Photobacterium damselae
0.00	1	1	S1	38294	Photobacterium damselae subsp. piscicida
0.00	12	1	O	135613	Chromatiales
0.00	9	0	F	1046	Chromatiaceae
0.00	8	1	G	67575	Rheinheimera
0.00	7	0	G1	115860	unclassified Rheinheimera
0.00	7	7	S	1763998	Rheinheimera sp. F8
0.00	1	0	G	156885	Thioflavicoccus
0.00	1	0	S	80679	Thioflavicoccus mobilis
0.00	1	1	S1	765912	Thioflavicoccus mobilis 8321
0.00	1	0	F	72276	Ectothiorhodospiraceae
0.00	1	0	G	106633	Thioalkalivibrio
0.00	1	1	S	106634	Thioalkalivibrio versutus
0.00	1	0	F	2035710	Thioalkalibacteraceae
0.00	1	0	G	2035712	Guyparkeria
0.00	1	1	S	47960	Guyparkeria halophila
0.00	10	0	O	135619	Oceanospirillales
0.00	8	0	F	28256	Halomonadaceae
0.00	7	0	G	2745	Halomonas
0.00	7	0	G1	2609666	unclassified Halomonas
0.00	6	6	S	2306583	Halomonas sp. JS92-SW72
0.00	1	1	S	2749040	Halomonas sp. SH5A2
0.00	1	0	G	3137766	Vreelandella
0.00	1	1	S	272774	Vreelandella alkaliphila
0.00	1	0	F	135620	Oceanospirillaceae
0.00	1	0	G	28253	Marinomonas
0.00	1	0	G1	196814	unclassified Marinomonas
0.00	1	1	S	2992802	Marinomonas sp. GJ51-6
0.00	1	0	F	224372	Alcanivoracaceae
0.00	1	0	G	3020832	Alloalcanivorax
0.00	1	0	S	285091	Alloalcanivorax dieselolei
0.00	1	1	S1	930169	Alloalcanivorax dieselolei B5
0.00	5	1	O	1706369	Cellvibrionales

0.00	4	0	F	1706371	Cellvibrionaceae
0.00	3	2	G	10	Cellvibrio
0.00	1	0	G1	2624793	unclassified Cellvibrio
0.00	1	1	S	1945512	Cellvibrio sp. PSBB023
0.00	1	0	G	2425	Teredinibacter
0.00	1	0	G1	2648457	unclassified Teredinibacter
0.00	1	1	S	3034506	Teredinibacter sp. KSP-S5-2
0.00	5	0	O	135625	Pasteurellales
0.00	5	0	F	712	Pasteurellaceae
0.00	4	0	G	416916	Aggregatibacter
0.00	3	3	S	732	Aggregatibacter aphrophilus
0.00	1	1	S	714	Aggregatibacter actinomycetemcomitans
0.00	1	0	G	724	Haemophilus
0.00	1	1	S	729	Haemophilus parainfluenzae
0.00	4	0	O	72273	Thiotrichales
0.00	2	0	F	135616	Piscirickettsiaceae
0.00	1	0	G	1237	Piscirickettsia
0.00	1	1	S	1238	Piscirickettsia salmonis
0.00	1	0	G	2781121	Thiosulfatimonas
0.00	1	1	S	2675054	Thiosulfatimonas sediminis
0.00	1	0	F	34064	Francisellaceae
0.00	1	0	G	262	Francisella
0.00	1	0	S	263	Francisella tularensis
0.00	1	1	S1	119857	Francisella tularensis subsp. holarctica
0.00	1	0	F	135617	Thiotrichaceae
0.00	1	0	G	1021	Beggiatoa
0.00	1	1	S	288004	Beggiatoa leptomitoformis
0.00	4	0	O	1240482	Orbales
0.00	4	1	F	1240483	Orbaceae
0.00	3	0	G	1193503	Gilliamella
0.00	1	1	S	1196095	Gilliamella apicola
0.00	1	1	S	1970738	Gilliamella apis
0.00	1	0	G1	2685620	unclassified Gilliamella
0.00	1	1	S	2817969	Gilliamella sp. B3022
0.00	3	0	O	135624	Aeromonadales
0.00	3	0	F	84642	Aeromonadaceae
0.00	3	0	G	642	Aeromonas
0.00	1	1	S	645	Aeromonas salmonicida

0.00	1	0	S	651	Aeromonas media
0.00	1	1	S1	1208104	Aeromonas media WS
0.00	1	0	G1	257493	unclassified Aeromonas
0.00	1	1	S	2033033	Aeromonas sp. CU5
0.00	3	0	O	118969	Legionellales
0.00	2	1	F	118968	Coxiellaceae
0.00	1	0	G	776	Coxiella
0.00	1	0	G1	2676648	unclassified Coxiella (in: g-proteobacteria)
0.00	1	1	S	2749996	Coxiella endosymbiont of Amblyomma nuttalli
0.00	1	0	F	444	Legionellaceae
0.00	1	0	G	445	Legionella
0.00	1	1	S	446	Legionella pneumophila
0.00	2	0	O	135618	Methylococcales
0.00	1	0	F	403	Methylococcaceae
0.00	1	0	G	416	Methylomonas
0.00	1	1	S	702114	Methylomonas koyamae
0.00	1	0	F	1486721	Methylothermaceae
0.00	1	1	G	1486720	Methylomarinovum
0.00	2	0	O	1692040	Acidiferrobacterales
0.00	2	0	F	1692041	Acidiferrobacteraceae
0.00	1	0	G	986106	Acidiferrobacter
0.00	1	0	G1	2640868	unclassified Acidiferrobacter
0.00	1	1	S	1281578	Acidiferrobacter sp. SPIII_3
0.00	1	0	G	1692042	Sulfurifustis
0.00	1	1	S	1675686	Sulfurifustis variabilis
0.00	2	1	C1	118884	Gammaproteobacteria incertae sedis
0.00	1	0	C2	32036	sulfur-oxidizing symbionts
0.00	1	1	S	2360	Bathymodiolus thermophilus thioautotrophic gill symbiont
0.00	1	0	O	3060226	Steroidobacterales
0.00	1	0	F	2689614	Steroidobacteraceae
0.00	1	0	G	469322	Steroidobacter
0.00	1	1	S	465721	Steroidobacter denitrificans
0.01	187	18	C	28216	Betaproteobacteria
0.01	155	18	O	80840	Burkholderiales
0.00	81	7	F	80864	Comamonadaceae
0.00	52	0	G	3051137	Paracidovorax
0.00	52	52	S	80867	Paracidovorax avenae
0.00	8	5	G	34072	Variovorax

0.00	2	0	S	34073	Variovorax paradoxus
0.00	2	2	S1	595537	Variovorax paradoxus EPS
0.00	1	0	G1	663243	unclassified Variovorax
0.00	1	1	S	282217	Variovorax sp. SRS16
0.00	6	1	G	283	Comamonas
0.00	2	0	S	32013	Comamonas terrigena
0.00	2	2	S1	1219032	Comamonas terrigena NBRC 13299
0.00	1	1	S	363952	Comamonas thiooxydans
0.00	1	1	S	379895	Comamonas odontotermitis
0.00	1	0	G1	2638500	unclassified Comamonas
0.00	1	1	S	2918299	Comamonas sp. B21-038
0.00	2	0	G	28065	Rhodoferax
0.00	1	1	S	1842727	Rhodoferax koreense
0.00	1	0	G1	2627954	unclassified Rhodoferax
0.00	1	1	S	2822760	Rhodoferax sp. PAMC 29310
0.00	2	1	G	80865	Delftia
0.00	1	0	G1	2613839	unclassified Delftia
0.00	1	1	S	3153805	Delftia sp. DS1230
0.00	2	0	G	665874	Limnohabitans
0.00	2	1	G1	2626134	unclassified Limnohabitans
0.00	1	1	S	1678128	Limnohabitans sp. 63ED37-2
0.00	1	0	G	201096	Alicycliphilus
0.00	1	1	S	179636	Alicycliphilus denitrificans
0.00	1	0	G	352450	Simplicispira
0.00	1	1	S	2109915	Simplicispira suum
0.00	32	7	F	75682	Oxalobacteraceae
0.00	13	3	F1	2895353	Telluria group
0.00	8	6	G	149698	Massilia
0.00	1	1	S	945844	Massilia oculi
0.00	1	0	G1	2609279	unclassified Massilia
0.00	1	1	S	2970464	Massilia sp. H6
0.00	1	0	G	212744	Rugamonas
0.00	1	0	G1	2620350	unclassified Rugamonas
0.00	1	1	S	3039386	Rugamonas sp. DEMB1
0.00	1	0	G	1522432	Pseudoduganella
0.00	1	1	S	2072590	Pseudoduganella armeniaca
0.00	7	1	G	29580	Janthinobacterium
0.00	4	0	S	55508	Janthinobacterium agaricidamnosum

0.00	4	4	S1	1349767	Janthinobacterium agaricidamnosum NBRC 102515 = DSM 9628
0.00	2	0	G1	2610881	unclassified Janthinobacterium
0.00	2	2	S	2497863	Janthinobacterium sp. 17J80-10
0.00	3	2	G	401469	Undibacterium
0.00	1	0	G1	2630295	unclassified Undibacterium
0.00	1	1	S	3048609	Undibacterium sp. CCC3.4
0.00	2	0	G	963	Herbaspirillum
0.00	1	1	S	964	Herbaspirillum seropedicae
0.00	1	1	S	92645	Herbaspirillum frisingense
0.00	11	2	F	119060	Burkholderiaceae
0.00	3	1	G	32008	Burkholderia
0.00	2	0	G1	87882	Burkholderia cepacia complex
0.00	1	1	S	95486	Burkholderia cenocepacia
0.00	1	1	S	488447	Burkholderia contaminans
0.00	2	1	G	44013	Polynucleobacter
0.00	1	0	G1	2640945	unclassified Polynucleobacter
0.00	1	1	S	2689107	Polynucleobacter sp. MWH-CaK5
0.00	2	0	G	47670	Lautropia
0.00	2	2	S	47671	Lautropia mirabilis
0.00	1	0	G	106589	Cupriavidus
0.00	1	1	S	151783	Cupriavidus campinensis
0.00	1	0	G	1822464	Paraburkholderia
0.00	1	1	S	2152891	Paraburkholderia bonniea
0.00	9	1	F	2975441	Sphaerotilaceae
0.00	3	0	G	34102	Sphaerotilus
0.00	3	3	S	639200	Sphaerotilus sulfidivorans
0.00	3	0	G	93681	Roseateles
0.00	1	0	G1	452439	environmental samples
0.00	1	1	S	452440	uncultured Roseateles sp.
0.00	1	1	S	2070761	Paucibacter aquatile
0.00	1	0	G1	2626991	unclassified Roseateles
0.00	1	1	S	1768242	Paucibacter sp. KCTC 42545
0.00	2	0	G	1114981	Piscinibacter
0.00	2	2	S	946333	Piscinibacter gummiphilus
0.00	4	2	F	506	Alcaligenaceae
0.00	1	1	G	507	Alcaligenes
0.00	1	0	G	222	Achromobacter

0.00	1	1	S	85698	Achromobacter xylosoxidans
0.00	6	0	O	206351	Neisseriales
0.00	3	0	F	1499392	Chromobacteriaceae
0.00	3	0	G	57739	Vogesella
0.00	3	0	G1	2684990	unclassified Vogesella
0.00	3	3	S	2877939	Vogesella sp. XCS3
0.00	2	1	F	2897177	Chitinibacteraceae
0.00	1	0	G	1055692	Chitinolyticbacter
0.00	1	1	S	682798	Chitinolyticbacter meiyuanensis
0.00	1	1	F	481	Neisseriaceae
0.00	4	0	O	32003	Nitrosomonadales
0.00	2	2	F	32011	Methylophilaceae
0.00	1	0	F	206379	Nitrosomonadaceae
0.00	1	0	G	914	Nitrosomonas
0.00	1	0	G1	2609265	unclassified Nitrosomonas
0.00	1	1	S	261292	Nitrosomonas sp. Is79A3
0.00	1	0	F	2772226	Sulfuricellaceae
0.00	1	0	G	1778653	Sulfuriferula
0.00	1	1	S	171865	Sulfuriferula plumbiphila
0.00	2	0	C1	119066	Betaproteobacteria incertae sedis
0.00	2	0	G	1301080	Candidatus Nasuia
0.00	2	2	S	1160784	Candidatus Nasuia deltocephalinicola
0.00	1	1	O	206389	Rhodocyclales
0.00	1	0	O	1442155	Ferrovales
0.00	1	0	F	1442156	Ferrovaceae
0.00	1	0	G	416212	Ferrovum
0.00	1	1	S	416213	Ferrovum myxofaciens
0.01	139	11	C	28211	Alphaproteobacteria
0.00	46	2	O	356	Hyphomicrobiales
0.00	14	11	F	41294	Nitrobacteraceae
0.00	3	0	G	374	Bradyrhizobium
0.00	1	1	S	1325090	Bradyrhizobium guangdongense
0.00	1	0	G1	2631580	unclassified Bradyrhizobium
0.00	1	1	S	2976822	Bradyrhizobium sp. CB1015
0.00	1	1	S	1437360	Bradyrhizobium erythrophlei
0.00	8	0	F	45401	Hyphomicrobiaceae
0.00	7	0	G	81	Hyphomicrobium
0.00	7	0	G1	2619925	unclassified Hyphomicrobium

0.00	7	7	S	3019544	Hyphomicrobium sp. DMF-1
0.00	1	1	G	1068	Rhodomicrobium
0.00	7	3	F	119045	Methylobacteriaceae
0.00	3	0	G	407	Methylobacterium
0.00	3	0	G1	2615210	unclassified Methylobacterium
0.00	3	3	S	2603276	Methylobacterium sp. WL1
0.00	1	0	G	186650	Microvirga
0.00	1	1	S	1882682	Microvirga ossetica
0.00	5	0	F	2831090	Blastochloridaceae
0.00	5	0	G	59282	Blastochloris
0.00	5	5	S	2233851	Blastochloris tepida
0.00	5	0	F	82115	Rhizobiaceae
0.00	5	1	F1	227290	Rhizobium/Agrobacterium group
0.00	2	1	G	379	Rhizobium
0.00	1	1	S	29449	Rhizobium etli
0.00	2	0	G	1612611	Pararhizobium
0.00	1	1	S	1395951	Pararhizobium gei
0.00	1	0	G1	2643050	unclassified Pararhizobium
0.00	1	1	S	2986923	Pararhizobium sp. BT-229
0.00	1	0	F	2831100	Boseaceae
0.00	1	1	G	85413	Bosea
0.00	1	0	F	69277	Phyllobacteriaceae
0.00	1	1	G	68287	Mesorhizobium
0.00	1	0	F	118882	Brucellaceae
0.00	1	0	F1	2826938	Brucella/Ochrobactrum group
0.00	1	0	G	234	Brucella
0.00	1	1	S	29459	Brucella melitensis
0.00	1	0	F	2813035	Parvibaculaceae
0.00	1	0	G	2838250	Kaustia
0.00	1	1	S	2593653	Kaustia mangrovi
0.00	1	0	F	655351	Cohaesibacteraceae
0.00	1	0	G	655352	Cohaesibacter
0.00	1	0	G1	2631913	unclassified Cohaesibacter
0.00	1	1	S	1798205	Cohaesibacter sp. ES.047
0.00	22	1	O	766	Rickettsiales
0.00	17	1	F	775	Rickettsiaceae
0.00	16	2	F1	33988	Rickettsieae
0.00	12	8	G	780	Rickettsia

0.00	3	0	G1	114295	unclassified Rickettsia
0.00	3	3	S	3066248	Rickettsia endosymbiont of Cantharis rufa
0.00	1	1	G1	114277	spotted fever group
0.00	2	0	G	2996317	Candidatus Tisiphia
0.00	2	0	G1	2996318	unclassified Candidatus Tisiphia
0.00	1	1	S	3066253	Candidatus Tisiphia endosymbiont of Dascillus cervinus
0.00	1	1	S	3066260	Candidatus Tisiphia endosymbiont of Ptychoptera albimana
0.00	4	0	F	942	Anaplasmataceae
0.00	4	0	F1	952	Wolbachieae
0.00	4	0	G	953	Wolbachia
0.00	4	0	G1	2640676	unclassified Wolbachia
0.00	4	4	S	3066146	Wolbachia endosymbiont (group A) of Lypha dubia
0.00	22	2	O	204457	Sphingomonadales
0.00	18	2	F	41297	Sphingomonadaceae
0.00	12	1	G	13687	Sphingomonas
0.00	3	0	G1	196159	unclassified Sphingomonas
0.00	1	1	S	1030157	Sphingomonas sp. KC8
0.00	1	1	S	3062169	Sphingomonas sp. C3-2
0.00	1	1	S	3014784	Sphingomonas sp. NIBR02145
0.00	2	2	S	2972485	Sphingomonas endolithica
0.00	1	1	S	2319844	Sphingomonas paeninsulae
0.00	1	1	S	2599297	Sphingomonas suaedae
0.00	1	1	S	1541170	Sphingomonas morindae
0.00	1	1	S	1327635	Sphingomonas psychrotolerans
0.00	1	1	S	2872652	Sphingomonas nostoxanthinifaciens
0.00	1	1	S	2698679	Sphingomonas changnyeongensis
0.00	2	1	G	165695	Sphingobium
0.00	1	0	G1	2611147	unclassified Sphingobium
0.00	1	1	S	3038927	Sphingobium sp. V4
0.00	1	0	G	2709685	Parasphingorhabdus
0.00	1	1	S	2806553	Parasphingorhabdus cellanae
0.00	1	1	G	165697	Sphingopyxis
0.00	1	1	F	335929	Erythrobacteraceae
0.00	1	0	F	2844881	Zymomonadaceae
0.00	1	0	G	541	Zymomonas
0.00	1	0	S	542	Zymomonas mobilis

0.00	1	0	S1	120044	Zymomonas mobilis subsp. pomaceae
0.00	1	1	S2	579138	Zymomonas mobilis subsp. pomaceae ATCC 29192
0.00	17	1	O	204441	Rhodospirillales
0.00	10	0	F	433	Acetobacteraceae
0.00	6	0	G	434	Acetobacter
0.00	3	3	S	446692	Acetobacter senegalensis
0.00	3	0	G1	2628570	unclassified Acetobacter
0.00	3	3	S	3157859	Acetobacter sp. A11-2
0.00	2	0	G	125216	Roseomonas
0.00	1	0	S	257708	Roseomonas gilardii
0.00	1	1	S1	204527	Roseomonas gilardii subsp. gilardii
0.00	1	1	S	2768162	Roseomonas haemaphysalidis
0.00	1	0	G	1079922	Commensalibacter
0.00	1	1	S	2070537	Commensalibacter melissae
0.00	1	0	G	2811380	Entomobacter
0.00	1	1	S	2762277	Entomobacter blattae
0.00	4	0	F	3031143	Magnetovibrionaceae
0.00	4	0	G	1866954	Varunaivibrio
0.00	4	4	S	1773489	Varunaivibrio sulfuroxidans
0.00	2	0	F	2829815	Azospirillaceae
0.00	1	0	G	191	Azospirillum
0.00	1	0	S	193	Azospirillum lipoferum
0.00	1	1	S1	862719	Azospirillum lipoferum 4B
0.00	1	0	G	204447	Skermanella
0.00	1	1	S	2233999	Skermanella pratensis
0.00	11	1	O	204455	Rhodobacterales
0.00	7	0	F	31989	Paracoccaceae
0.00	5	2	G	265	Paracoccus
0.00	3	0	G1	2688777	unclassified Paracoccus (in: a-proteobacteria)
0.00	2	2	S	2589076	Paracoccus sp. AK26
0.00	1	1	S	3030011	Paracoccus sp. S3-43
0.00	1	0	G	2211635	Cognatishimia
0.00	1	1	S	1715691	Cognatishimia activa
0.00	1	0	G	204456	Gemmobacter
0.00	1	1	S	2169400	Gemmobacter aquarius
0.00	3	0	F	2854170	Roseobacteraceae
0.00	2	1	G	60136	Sulfitobacter
0.00	1	1	S	83219	Sulfitobacter mediterraneus

0.00	1	0	G	74030	Roseovarius
0.00	1	0	G1	2614913	unclassified Roseovarius
0.00	1	1	S	3080976	Roseovarius sp. S88
0.00	4	0	O	204458	Caulobacterales
0.00	4	0	F	76892	Caulobacteraceae
0.00	4	3	G	41275	Brevundimonas
0.00	1	0	G1	2622653	unclassified Brevundimonas
0.00	1	1	S	2560058	Brevundimonas sp. Bb-A
0.00	3	0	O	1921002	Holosporales
0.00	3	0	F	1777752	Candidatus Paracaedibacteraceae
0.00	3	0	G	3163645	Candidatus Bodocaedibacter
0.00	3	3	S	2741701	Candidatus Bodocaedibacter vickermanii
0.00	2	0	O	54526	Candidatus Pelagibacterales
0.00	2	0	F	1655514	Candidatus Pelagibacteraceae
0.00	2	0	G	198251	Candidatus Pelagibacter
0.00	2	0	G1	2647897	unclassified Candidatus Pelagibacter
0.00	1	1	S	1002672	Candidatus Pelagibacter sp. IMCC9063
0.00	1	1	S	2268451	Candidatus Pelagibacter sp. FZCC0015
0.00	1	0	O	362534	Kordiimonadales
0.00	1	0	F	1331809	Kordiimonadaceae
0.00	1	0	G	288021	Kordiimonas
0.00	1	0	G1	2618732	unclassified Kordiimonas
0.00	1	1	S	2829596	Kordiimonas sp. SCSIO 12603
0.06	1203	14	D1	1783272	Terrabacteria group
0.03	621	0	D2	1798711	Cyanobacteriota/Melainabacteria group
0.03	621	0	P	1117	Cyanobacteriota
0.03	620	99	C	3028117	Cyanophyceae
0.02	458	0	O	1890424	Synechococcales
0.02	434	0	F	1890428	Merismopediaceae
0.02	434	0	G	1142	Synechocystis
0.02	434	0	G1	2640012	unclassified Synechocystis
0.02	434	434	S	3144951	Synechocystis sp. LKSZ1
0.00	24	0	F	1890426	Synechococcaceae
0.00	24	0	G	1129	Synechococcus
0.00	24	12	G1	2626047	unclassified Synechococcus
0.00	11	11	S	166314	Synechococcus sp. WH 8109
0.00	1	1	S	321332	Synechococcus sp. JA-2-3B'a(2-13)
0.00	39	0	C1	1301283	Oscillatoriophycideae

0.00	29	0	O	1118	Chroococcales
0.00	15	0	F	3079756	Halothecacae
0.00	15	0	F1	92682	Halothece cluster
0.00	15	0	G	76023	Halothece
0.00	15	0	G1	2632878	unclassified Halothece
0.00	15	15	S	65093	Halothece sp. PCC 7418
0.00	8	6	F	2815910	Geminocystaceae
0.00	1	0	G	669357	Geminocystis
0.00	1	0	G1	2625037	unclassified Geminocystis
0.00	1	1	S	1617448	Geminocystis sp. NIES-3709
0.00	1	0	G	3079908	Picosynechococcus
0.00	1	0	G1	3079910	unclassified Picosynechococcus
0.00	1	1	S	32049	Picosynechococcus sp. PCC 7002
0.00	6	0	F	1890450	Aphanothecaceae
0.00	6	0	G	1453359	Candidatus Atelocyanobacterium
0.00	6	0	S	713887	Candidatus Atelocyanobacterium thalassa
0.00	5	5	S1	1285375	cyanobacterium endosymbiont of Braarudosphaera bigelowii
0.00	1	1	S1	1453429	Candidatus Atelocyanobacterium thalassa isolate ALOHA
0.00	10	0	O	1150	Oscillatoriales
0.00	4	0	F	1892254	Oscillatoriaceae
0.00	4	0	G	1158	Oscillatoria
0.00	3	0	S	118323	Oscillatoria acuminata
0.00	3	3	S1	56110	Oscillatoria acuminata PCC 6304
0.00	1	0	S	482564	Oscillatoria nigro-viridis
0.00	1	1	S1	179408	Oscillatoria nigro-viridis PCC 7112
0.00	4	0	F	2934961	Sirenicapillariaceae
0.00	4	1	G	2596745	Limnospira
0.00	3	0	S	147322	Limnospira indica
0.00	3	3	S1	2760438	Limnospira indica BM01
0.00	2	0	F	1892252	Microcoleaceae
0.00	2	1	G	54304	Planktothrix
0.00	1	0	S	1160	Planktothrix agardhii
0.00	1	1	S1	388467	Planktothrix agardhii NIVA-CYA 126/8
0.00	9	0	O	1161	Nostocales
0.00	6	0	F	1162	Nostocaceae
0.00	5	1	G	1177	Nostoc

0.00	4	0	G1	2593658	unclassified Nostoc
0.00	4	4	S	2576904	Nostoc sp. TCL26-01
0.00	1	0	G	1163	Anabaena
0.00	1	0	G1	2619674	unclassified Anabaena
0.00	1	1	S	46234	Anabaena sp. 90
0.00	2	0	F	1185	Rivulariaceae
0.00	2	0	G	373984	Rivularia
0.00	2	0	G1	2676603	unclassified Rivularia (in: cyanobacteria)
0.00	2	2	S	373994	Rivularia sp. PCC 7116
0.00	1	0	F	119859	Tolypothrichaceae
0.00	1	0	G	111782	Tolypothrix
0.00	1	1	G1	2649714	unclassified Tolypothrix
0.00	6	0	O	3079749	Leptolyngbyales
0.00	6	0	F	1890438	Leptolyngbyaceae
0.00	5	0	F1	3081713	Leptolyngbya group
0.00	5	0	G	47251	Leptolyngbya
0.00	5	0	G1	2650499	unclassified Leptolyngbya
0.00	5	5	S	2812896	Leptolyngbya sp. 7M
0.00	1	0	G	1874526	Kovacikia
0.00	1	0	S	2931930	Kovacikia minuta
0.00	1	1	S1	2653194	Kovacikia minuta CCNUW1
0.00	2	0	O	52604	Pleurocapsales
0.00	2	0	F	1890500	Hyellaceae
0.00	2	0	G	44474	Pleurocapsa
0.00	2	0	G1	2645907	unclassified Pleurocapsa
0.00	2	2	S	118163	Pleurocapsa sp. PCC 7327
0.00	2	0	O	3079744	Acaryochloridales
0.00	2	0	F	2881378	Thermosynechococcaceae
0.00	2	0	G	146785	Thermosynechococcus
0.00	1	0	G1	2622553	unclassified Thermosynechococcus
0.00	1	1	S	1394889	Thermosynechococcus sp. NK55a
0.00	1	0	S	3161974	Thermosynechococcus sichuanensis
0.00	1	1	S1	2016101	Thermosynechococcus sichuanensis E542
0.00	1	0	O	307595	Gloeobacterales
0.00	1	0	F	1890422	Gloeobacteraceae
0.00	1	1	G	33071	Gloeobacter
0.00	1	0	O	1890505	Chroococcidiopsidales
0.00	1	0	F	1890528	Chroococcidiopsidaceae

0.00	1	1	G	54298	Chroococcidiopsis
0.00	1	0	O	2881383	Thermostichales
0.00	1	0	F	2881384	Thermostichaceae
0.00	1	0	G	2881385	Thermostichus
0.00	1	0	S	33070	Thermostichus lividus
0.00	1	1	S1	1917166	Thermostichus lividus PCC 6715
0.00	1	0	O	3079748	Oculatellales
0.00	1	0	F	2303507	Oculatellaceae
0.00	1	0	G	3065395	Thermocoleostomius
0.00	1	0	S	3065396	Thermocoleostomius sinensis
0.00	1	1	S1	2016057	Thermocoleostomius sinensis A174
0.00	1	0	O	3079757	Gomontiellales
0.00	1	0	F	1892255	Gomontiellaceae
0.00	1	0	G	241421	Crinalium
0.00	1	0	S	241425	Crinalium epipsammum
0.00	1	1	S1	1173022	Crinalium epipsammum PCC 9333
0.00	1	0	P1	1983111	unclassified Cyanobacteriota
0.00	1	0	S	718217	cyanobacterium endosymbiont of Epithemia turgida
0.00	1	1	S1	1228987	cyanobacterium endosymbiont of Epithemia turgida isolate EtSB Lake Yunoko
0.02	348	46	P	1239	Bacillota
0.01	159	3	C	91061	Bacilli
0.01	106	4	O	1385	Bacillales
0.00	62	1	F	186817	Bacillaceae
0.00	25	2	G	1386	Bacillus
0.00	12	3	G1	86661	Bacillus cereus group
0.00	4	4	S	1396	Bacillus cereus
0.00	2	2	S	1405	Bacillus mycoides
0.00	2	2	S	1392	Bacillus anthracis
0.00	1	1	S	64104	Bacillus pseudomycoides
0.00	8	0	G1	653685	Bacillus subtilis group
0.00	4	0	S	1452	Bacillus atrophaeus
0.00	4	4	S1	1239783	Bacillus atrophaeus UCMB-5137
0.00	3	0	G2	1938374	Bacillus amyloliquefaciens group
0.00	2	2	S	492670	Bacillus velezensis
0.00	1	1	S	1390	Bacillus amyloliquefaciens
0.00	1	1	S	1423	Bacillus subtilis
0.00	2	1	G1	185979	unclassified Bacillus (in: firmicutes)

0.00	1	1	S	3077585	Bacillus sp. DTU_2020_1000418_1_SI_GHA_SEK_038
0.00	1	1	S	1408	Bacillus pumilus
0.00	4	4	G	2837504	Heyndrickxia
0.00	3	0	G	484508	Ornithinibacillus
0.00	3	0	G1	2620869	unclassified Ornithinibacillus
0.00	3	3	S	3231488	Ornithinibacillus sp. 4-3
0.00	3	0	G	2675232	Neobacillus
0.00	2	0	G1	2675272	unclassified Neobacillus
0.00	2	2	S	3048535	Neobacillus sp. 114
0.00	1	1	S	220684	Neobacillus drentensis
0.00	3	0	G	84406	Virgibacillus
0.00	1	1	S	1482	Virgibacillus halodenitrificans
0.00	1	1	S	163877	Virgibacillus necropolis
0.00	1	1	S	302167	Virgibacillus dokdonensis
0.00	2	0	G	2675230	Cytobacillus
0.00	2	2	S	665099	Cytobacillus oceanisediminis
0.00	2	0	G	2675229	Peribacillus
0.00	2	2	S	450367	Peribacillus frigoritolerans
0.00	2	0	G	182709	Oceanobacillus
0.00	2	1	G1	2630292	unclassified Oceanobacillus
0.00	1	1	S	2921698	Oceanobacillus sp. FSL W7-1281
0.00	2	1	G	2685905	Salicibibacter
0.00	1	1	S	2743000	Salicibibacter cibarius
0.00	2	1	G	2800373	Priestia
0.00	1	1	S	1404	Priestia megaterium
0.00	2	2	G	400634	Lysinibacillus
0.00	2	0	G	1221880	Psychrobacillus
0.00	1	1	S	2283160	Psychrobacillus glaciei
0.00	1	1	G1	2636677	unclassified Psychrobacillus
0.00	2	0	G	2837510	Siminovitchia
0.00	2	2	S	254758	Siminovitchia fortis
0.00	1	0	G	2675233	Metabacillus
0.00	1	1	S	930124	Metabacillus halosaccharovorans
0.00	1	0	G	74385	Gracilibacillus
0.00	1	1	S	2663022	Gracilibacillus salitolerans
0.00	1	0	G	2785518	Radiobacillus
0.00	1	1	S	2844358	Radiobacillus kanasensis
0.00	1	0	G	1884449	Salipaludibacillus

0.00	1	0	G1	2635229	unclassified Salipaludibacillus
0.00	1	1	S	2924031	Salipaludibacillus sp. LMS25
0.00	1	0	G	2837485	Evansella
0.00	1	0	S	1413	Evansella cellulosilytica
0.00	1	1	S1	649639	Evansella cellulosilytica DSM 2522
0.00	1	0	G	2837508	Rossellomorea
0.00	1	1	S	189382	Rossellomorea aquimaris
0.00	1	0	G	129337	Geobacillus
0.00	1	0	G1	2642459	unclassified Geobacillus
0.00	1	1	S	391290	Geobacillus sp. E263
0.00	17	0	F	90964	Staphylococcaceae
0.00	14	4	G	1279	Staphylococcus
0.00	2	2	S	1282	Staphylococcus epidermidis
0.00	1	0	G1	3239053	Staphylococcus cohnii species complex
0.00	1	1	S	29382	Staphylococcus cohnii
0.00	1	0	G1	91994	unclassified Staphylococcus
0.00	1	1	S	2929481	Staphylococcus sp. IVB6181
0.00	1	1	S	1280	Staphylococcus aureus
0.00	1	1	S	214473	Staphylococcus nepalensis
0.00	1	1	S	29385	Staphylococcus saprophyticus
0.00	1	1	S	985762	Staphylococcus agnetis
0.00	1	0	G1	2815305	Staphylococcus intermedius group
0.00	1	1	S	283734	Staphylococcus pseudintermedius
0.00	1	1	S	1286	Staphylococcus simulans
0.00	2	0	G	2803850	Mammaliicoccus
0.00	2	2	S	1296	Mammaliicoccus sciuri
0.00	1	0	G	227979	Jeotgalicoccus
0.00	1	0	G1	2630462	unclassified Jeotgalicoccus
0.00	1	1	S	2708346	Jeotgalicoccus sp. WY2
0.00	14	0	F	186822	Paenibacillaceae
0.00	12	1	G	44249	Paenibacillus
0.00	7	2	G1	185978	unclassified Paenibacillus
0.00	1	1	S	2975345	Paenibacillus sp. FSL H7-0350
0.00	1	1	S	2758563	Paenibacillus sp. 19GGS1-52
0.00	1	1	S	2954532	Paenibacillus sp. FSL R7-0313
0.00	1	1	S	1566358	Paenibacillus sp. IHBB 10380
0.00	1	1	S	2921604	Paenibacillus sp. FSL L8-0638
0.00	1	1	S	1406	Paenibacillus polymyxa

0.00	1	1	S	169760	Paenibacillus stellifer
0.00	1	1	S	1763538	Paenibacillus crassostreae
0.00	1	1	S	44251	Paenibacillus durus
0.00	1	0	F1	85151	Aneurinibacillus group
0.00	1	0	G	55079	Aneurinibacillus
0.00	1	0	G1	2642329	unclassified Aneurinibacillus
0.00	1	1	S	3022697	Aneurinibacillus sp. Ricciae_BoGa-3
0.00	1	0	G	329857	Cohnella
0.00	1	1	S	2598458	Cohnella cholangitidis
0.00	4	0	F	186818	Planococcaceae
0.00	3	0	G	1372	Planococcus
0.00	2	0	S	161360	Planococcus antarcticus
0.00	2	2	S1	1185653	Planococcus antarcticus DSM 14505
0.00	1	1	S	1302659	Planococcus versutus
0.00	1	0	G	496496	Viridibacillus
0.00	1	0	G1	2617942	unclassified Viridibacillus
0.00	1	1	S	2779527	Viridibacillus sp. JNUCC-6
0.00	2	0	F	186820	Listeriaceae
0.00	2	0	G	1637	Listeria
0.00	2	2	S	1642	Listeria innocua
0.00	1	0	F	186824	Thermoactinomycetaceae
0.00	1	0	G	2689589	Staphylospora
0.00	1	1	S	2490858	Staphylospora marina
0.00	1	0	O1	539002	Bacillales incertae sedis
0.00	1	0	O2	539742	Bacillales Family XII. Incertae Sedis
0.00	1	1	G	33986	Exiguobacterium
0.00	1	0	F	539738	Gemellaceae
0.00	1	0	G	1378	Gemella
0.00	1	1	S	84135	Gemella sanguinis
0.00	50	1	O	186826	Lactobacillales
0.00	20	2	F	33958	Lactobacillaceae
0.00	6	0	G	2767885	Latilactobacillus
0.00	6	6	S	28038	Latilactobacillus curvatus
0.00	3	0	G	1578	Lactobacillus
0.00	1	1	S	1587	Lactobacillus helveticus
0.00	1	0	S	47770	Lactobacillus crispatus
0.00	1	1	S1	748671	Lactobacillus crispatus ST1
0.00	1	1	S	2012495	Lactobacillus panisapium

0.00	3	0	G	46255	Weissella
0.00	3	3	S	759620	Weissella ceti
0.00	2	2	G	1243	Leuconostoc
0.00	1	0	G	2759736	Lacticaseibacillus
0.00	1	0	S	1597	Lacticaseibacillus paracasei
0.00	1	1	S1	47714	Lacticaseibacillus paracasei subsp. paracasei
0.00	1	0	G	2767890	Paucilactobacillus
0.00	1	0	S	1193095	Paucilactobacillus hokkaidonensis
0.00	1	1	S1	1291742	Paucilactobacillus hokkaidonensis JCM 18461
0.00	1	0	G	2767887	Ligilactobacillus
0.00	1	1	S	1624	Ligilactobacillus salivarius
0.00	1	0	G	2767881	Fructilactobacillus
0.00	1	1	S	1625	Fructilactobacillus sanfranciscensis
0.00	19	0	F	1300	Streptococcaceae
0.00	16	6	G	1301	Streptococcus
0.00	2	2	S	1314	Streptococcus pyogenes
0.00	1	1	S	1304	Streptococcus salivarius
0.00	1	0	G1	119603	Streptococcus dysgalactiae group
0.00	1	1	S	1336	Streptococcus equi
0.00	1	1	S	254785	Streptococcus halichoeri
0.00	1	0	G1	2608887	unclassified Streptococcus
0.00	1	1	S	1759399	Streptococcus sp. A12
0.00	1	1	S	1313	Streptococcus pneumoniae
0.00	1	1	S	1340	Streptococcus porcinus
0.00	1	1	S	68892	Streptococcus infantis
0.00	1	0	G1	671232	Streptococcus anginosus group
0.00	1	0	S	76860	Streptococcus constellatus
0.00	1	0	S1	184250	Streptococcus constellatus subsp. pharyngis
0.00	1	1	S2	696216	Streptococcus constellatus subsp. pharyngis C232
0.00	3	1	G	1357	Lactococcus
0.00	2	1	S	1359	Lactococcus cremoris
0.00	1	0	S1	2816960	Lactococcus cremoris subsp. cremoris
0.00	1	1	S2	1295826	Lactococcus cremoris subsp. cremoris KW2
0.00	6	0	F	81852	Enterococcaceae
0.00	4	0	G	1350	Enterococcus
0.00	2	2	S	1352	Enterococcus faecium
0.00	1	1	S	37734	Enterococcus casseliflavus
0.00	1	1	S	53345	Enterococcus durans

0.00	2	0	G	51668	Tetragenococcus
0.00	1	1	S	290335	Tetragenococcus koreensis
0.00	1	1	S	526944	Tetragenococcus osmophilus
0.00	4	1	F	186828	Carnobacteriaceae
0.00	2	2	G	29393	Dolosigranulum
0.00	1	1	G	1470540	Jeotgalibaca
0.00	81	5	C	186801	Clostridia
0.00	48	3	O	186802	Eubacteriales
0.00	32	0	F	31979	Clostridiaceae
0.00	27	7	G	1485	Clostridium
0.00	4	4	S	1491	Clostridium botulinum
0.00	3	0	G1	2614128	unclassified Clostridium
0.00	1	1	S	641107	Clostridium sp. DL-VIII
0.00	1	1	S	3070681	Clostridium sp. OS1-26
0.00	1	1	S	1970093	Clostridium sp. 001
0.00	3	3	S	1502	Clostridium perfringens
0.00	2	2	S	94869	Clostridium gasigenes
0.00	2	2	S	238834	Clostridium estertheticum
0.00	1	1	S	84022	Clostridium aceticum
0.00	1	0	S	1501	Clostridium pasteurianum
0.00	1	1	S1	86416	Clostridium pasteurianum BC1
0.00	1	1	S	36839	Clostridium felsineum
0.00	1	1	S	36745	Clostridium saccharoperbutylacetonicum
0.00	1	1	S	1520	Clostridium beijerinckii
0.00	1	0	S	1542	Clostridium novyi
0.00	1	1	S1	386415	Clostridium novyi NT
0.00	5	0	G	1848399	Crassaminicella
0.00	4	4	S	2855394	Crassaminicella indica
0.00	1	1	S	2599308	Crassaminicella thermophila
0.00	7	4	F	216572	Oscillospiraceae
0.00	2	0	G	1508657	Ruminiclostridium
0.00	2	2	S	2488810	Ruminiclostridium herbifermentans
0.00	1	0	G	216851	Faecalibacterium
0.00	1	1	S	411483	Faecalibacterium duncaniae
0.00	2	1	F	186807	Peptococcaceae
0.00	1	0	G	471826	Candidatus Desulforudis
0.00	1	1	S	471827	Candidatus Desulforudis audaxviator
0.00	2	0	O1	538999	Eubacteriales incertae sedis

0.00	2	0	G	2717089	Massilistercora
0.00	2	2	S	2086584	Massilistercora timonensis
0.00	1	0	F	186806	Eubacteriaceae
0.00	1	0	G	33951	Acetobacterium
0.00	1	0	S	33952	Acetobacterium woodii
0.00	1	1	S1	931626	Acetobacterium woodii DSM 1030
0.00	1	0	F	2937909	Desulfitobacteriaceae
0.00	1	0	G	36853	Desulfitobacterium
0.00	1	1	S	49338	Desulfitobacterium hafniense
0.00	13	0	O	3085636	Lachnospirales
0.00	13	2	F	186803	Lachnospiraceae
0.00	3	0	G	207244	Anaerostipes
0.00	1	1	S	649756	Anaerostipes hadrus
0.00	1	1	S	1229621	Anaerostipes rhamnosivorans
0.00	1	0	G1	2635253	unclassified Anaerostipes
0.00	1	1	S	3036926	Anaerostipes sp. PC18
0.00	2	2	G	2719231	Lacrimispora
0.00	2	0	G	572511	Blautia
0.00	1	0	S	1322	Blautia hansenii
0.00	1	1	S1	537007	Blautia hansenii DSM 20583
0.00	1	1	S	89014	Blautia luti
0.00	1	0	G	1843210	Anaerocolumna
0.00	1	0	G1	2642604	unclassified Anaerocolumna
0.00	1	1	S	3070997	Anaerocolumna sp. MB42-C2
0.00	1	0	G	1766253	Agathobacter
0.00	1	1	S	39491	Agathobacter rectalis
0.00	1	0	G	2719313	Enterocloster
0.00	1	1	S	208479	Enterocloster bolteae
0.00	1	0	G	189330	Dorea
0.00	1	1	S	39486	Dorea formicigenerans
0.00	9	0	O	3082720	Peptostreptococcales
0.00	7	0	F	186804	Peptostreptococcaceae
0.00	3	0	G	1870884	Clostridioides
0.00	3	3	S	1496	Clostridioides difficile
0.00	1	0	G	214904	Tepidibacter
0.00	1	1	S	3036126	Tepidibacter hydrothermalis
0.00	1	0	G	1505652	Terrisporobacter
0.00	1	1	S	1460447	Terrisporobacter petrolearius

0.00	1	0	G	1849822	Paraclostridium
0.00	1	1	S	1490	Paraclostridium bifermentans
0.00	1	0	G	2743582	Peptacetobacter
0.00	1	1	S	89152	Peptacetobacter hiranonis
0.00	1	0	F	3030910	Anaerovoracaceae
0.00	1	0	G	2060094	Aminipila
0.00	1	1	S	2507160	Aminipila luticellarii
0.00	1	0	F	3118655	Filifactoraceae
0.00	1	0	G	186831	Acetoanaerobium
0.00	1	1	S	1511	Acetoanaerobium sticklandii
0.00	2	1	O	53433	Halanaerobiales
0.00	1	1	F	972	Halanaerobiaceae
0.00	2	0	O	68295	Thermoanaerobacterales
0.00	2	0	F	186814	Thermoanaerobacteraceae
0.00	2	1	G	28895	Thermoanaerobacterium
0.00	1	0	S	29329	Thermoanaerobacterium xylanolyticum
0.00	1	1	S1	858215	Thermoanaerobacterium xylanolyticum LX-11
0.00	1	0	O	3039167	Moorellales
0.00	1	0	F	2748673	Zhaonellaceae
0.00	1	0	G	2748674	Zhaonella
0.00	1	1	S	2528593	Zhaonella formicivorans
0.00	1	0	O	3082768	Christensenellales
0.00	1	0	F	990719	Christensenellaceae
0.00	1	1	G	990721	Christensenella
0.00	42	0	C	909932	Negativicutes
0.00	40	0	O	909929	Selenomonadales
0.00	38	0	F	1843490	Sporomusaceae
0.00	38	19	G	2375	Sporomusa
0.00	13	0	S	47679	Sporomusa sphaeroides
0.00	13	13	S1	1337886	Sporomusa sphaeroides DSM 2875
0.00	3	0	S	2378	Sporomusa ovata
0.00	3	3	S1	1123288	Sporomusa ovata DSM 2662
0.00	1	1	S	112901	Sporomusa malonica
0.00	1	1	S	204936	Sporomusa aerivorans
0.00	1	1	S	357999	Sporomusa rhizae
0.00	2	0	F	1843491	Selenomonadaceae
0.00	1	0	G	970	Selenomonas
0.00	1	1	S	69823	Selenomonas sputigena

0.00	1	0	G	158846	Megamonas
0.00	1	1	S	158847	Megamonas hypermegale
0.00	1	0	O	1843488	Acidaminococcales
0.00	1	0	F	909930	Acidaminococcaceae
0.00	1	0	G	33024	Phascolarctobacterium
0.00	1	1	S	33025	Phascolarctobacterium faecium
0.00	1	0	O	1843489	Veillonellales
0.00	1	0	F	31977	Veillonellaceae
0.00	1	0	G	29465	Veillonella
0.00	1	1	S	423477	Veillonella rogosae
0.00	9	0	C	526524	Erysipelotrichia
0.00	9	0	O	526525	Erysipelotrichales
0.00	8	0	F	128827	Erysipelotrichaceae
0.00	6	0	G	1647	Erysipelothrix
0.00	3	0	S	1648	Erysipelothrix rhusiopathiae
0.00	3	3	S1	3119429	Erysipelothrix rhusiopathiae subsp. ohloneorum
0.00	2	2	S	1514105	Erysipelothrix larvae
0.00	1	1	S	225084	Erysipelothrix inopinata
0.00	2	0	G	118747	Bulleidia
0.00	2	0	G1	2704656	unclassified Bulleidia
0.00	2	2	S	2806552	Bulleidia sp. zg-1006
0.00	1	0	F	2810280	Coprobacillaceae
0.00	1	0	G	2678885	Faecalibacillus
0.00	1	1	S	1982626	Faecalibacillus intestinalis
0.00	8	0	C	1737404	Tissierellia
0.00	8	0	O	1737405	Tissierellales
0.00	8	0	F	1570339	Peptoniphilaceae
0.00	3	0	G	165779	Anaerococcus
0.00	3	3	S	1755241	Anaerococcus nagyae
0.00	3	0	G	543311	Parvimonas
0.00	3	3	S	33033	Parvimonas micra
0.00	1	0	G	150022	Finegoldia
0.00	1	1	S	1260	Finegoldia magna
0.00	1	1	G	162289	Peptoniphilus
0.00	2	0	P1	2840488	Bacillota incertae sedis
0.00	2	0	O	3071001	Caldicellulosiruptorales
0.00	2	0	F	3071002	Caldicellulosiruptoraceae
0.00	2	2	G	44000	Caldicellulosiruptor

0.00	1	0	P1	33974	Bacillota sensu stricto incertae sedis
0.00	1	0	G	1930845	Ndongobacter
0.00	1	1	S	1871025	Ndongobacter massiliensis
0.01	182	0	P	201174	Actinomycetota
0.01	170	17	C	1760	Actinomycetes
0.00	43	0	O	85011	Kitasatosporales
0.00	43	0	F	2062	Streptomycetaceae
0.00	39	7	G	1883	Streptomyces
0.00	22	2	G1	2593676	unclassified Streptomyces
0.00	9	9	S	477697	Streptomyces sp. T12
0.00	1	1	S	2900152	Streptomyces sp. LX-29
0.00	1	1	S	2931974	Streptomyces sp. 2P-4
0.00	1	1	S	2903788	Streptomyces sp. NBC_01235
0.00	1	1	S	2903649	Streptomyces sp. NBC_00435
0.00	1	1	S	2975733	Streptomyces sp. NBC_00385
0.00	1	1	S	2742137	Streptomyces sp. NA02950
0.00	1	1	S	2750011	Streptomyces sp. Rer75
0.00	1	1	S	2903869	Streptomyces sp. NBC_01445
0.00	1	1	S	2840373	Streptomyces sp. YPW6
0.00	1	1	S	2135430	Streptomyces sp. P3
0.00	1	1	S	2855836	Streptomyces sp. WY228
0.00	2	2	S	67280	Streptomyces bobili
0.00	1	1	S	1893	Streptomyces atratus
0.00	1	1	S	35621	Streptomyces mobaraensis
0.00	1	1	S	68270	Streptomyces spectabilis
0.00	1	1	S	68286	Streptomyces zaomyceticus
0.00	1	0	G1	1477431	Streptomyces albidoflavus group
0.00	1	1	S	1886	Streptomyces albidoflavus
0.00	1	1	S	1907	Streptomyces glaucescens
0.00	1	1	S	68231	Streptomyces longwoodensis
0.00	1	1	S	54571	Streptomyces venezuelae
0.00	4	0	G	2063	Kitasatospora
0.00	4	0	G1	2633591	unclassified Kitasatospora
0.00	4	4	S	2018025	Kitasatospora sp. MMS16-BH015
0.00	37	4	O	85006	Micrococcales
0.00	16	1	F	1268	Micrococcaceae
0.00	6	0	G	1663	Arthrobacter
0.00	4	0	G1	235627	unclassified Arthrobacter

0.00	2	2	S	2830993	Arthrobacter sp. StoSoilB13
0.00	1	1	S	2830990	Arthrobacter sp. StoSoilA2
0.00	1	1	S	2828862	Arthrobacter sp. Helios
0.00	1	1	S	37928	Arthrobacter crystallopoietes
0.00	1	1	S	656366	Arthrobacter alpinus
0.00	4	1	G	32207	Rothia
0.00	2	2	S	43675	Rothia mucilaginosa
0.00	1	1	S	396015	Rothia terrae
0.00	2	1	G	57493	Kocuria
0.00	1	1	S	1275	Kocuria rosea
0.00	2	1	G	1269	Micrococcus
0.00	1	1	S	2856555	Micrococcus porci
0.00	1	0	G	1742993	Pseudarthrobacter
0.00	1	0	G1	2647000	unclassified Pseudarthrobacter
0.00	1	1	S	2851598	Pseudarthrobacter sp. L1SW
0.00	12	3	F	85023	Microbacteriaceae
0.00	4	2	G	33882	Microbacterium
0.00	1	1	S	69362	Microbacterium schleiferi
0.00	1	0	G1	2609290	unclassified Microbacterium
0.00	1	1	S	2909588	Microbacterium sp. KUDC0406
0.00	2	2	G	33886	Rathayibacter
0.00	1	0	F1	1655488	Luna cluster
0.00	1	0	F2	1655489	Luna-1 subcluster
0.00	1	0	G	529883	Rhodoluna
0.00	1	1	S	535712	Candidatus Rhodoluna planktonica
0.00	1	0	G	881616	Herbiconiux
0.00	1	0	G1	2618217	unclassified Herbiconiux
0.00	1	1	S	2905871	Herbiconiux sp. L3-i23
0.00	1	0	G	96492	Frigoribacterium
0.00	1	0	G1	2627005	unclassified Frigoribacterium
0.00	1	1	S	2596916	Frigoribacterium sp. NBH87
0.00	4	3	F	85021	Intrasporangiaceae
0.00	1	0	G	267408	Arsenicicoccus
0.00	1	0	G1	2663846	unclassified Arsenicicoccus
0.00	1	1	S	1658671	Arsenicicoccus sp. oral taxon 190
0.00	1	0	F	85020	Dermabacteraceae
0.00	1	1	G	36739	Dermabacter
0.00	34	1	O	85009	Propionibacteriales

0.00	18	1	F	31957	Propionibacteriaceae
0.00	10	0	G	1912216	Cutibacterium
0.00	7	7	S	1747	Cutibacterium acnes
0.00	3	3	S	33011	Cutibacterium granulosum
0.00	4	0	G	29404	Microlunatus
0.00	2	0	S	29405	Microlunatus phosphovorus
0.00	2	2	S1	1032480	Microlunatus phosphovorus NM-1
0.00	2	2	S	546874	Microlunatus sagamiharensis
0.00	1	0	G	1743	Propionibacterium
0.00	1	1	S	1744	Propionibacterium freudenreichii
0.00	1	0	G	72763	Tessaracoccus
0.00	1	1	S	1332264	Tessaracoccus aquimaris
0.00	1	0	G	2801844	Arachnia
0.00	1	1	S	1750	Arachnia propionica
0.00	14	3	F	85015	Nocardioidaceae
0.00	5	2	G	2040	Aeromicrobium
0.00	2	2	S	2969247	Aeromicrobium wangtongii
0.00	1	1	S	2041	Aeromicrobium erythreum
0.00	2	0	G	1839	Nocardioides
0.00	2	2	S	642780	Nocardioides scoriae
0.00	2	0	G	116071	Micropruina
0.00	2	2	S	75385	Micropruina glycogenica
0.00	1	0	G	2044	Pimelobacter
0.00	1	1	S	2045	Pimelobacter simplex
0.00	1	0	G	53387	Friedmanniella
0.00	1	1	S	546871	Friedmanniella luteola
0.00	1	0	F	2726069	Kribbellaceae
0.00	1	0	G	182639	Kribbella
0.00	1	0	G1	2644121	unclassified Kribbella
0.00	1	1	S	2903578	Kribbella sp. NBC_01245
0.00	28	2	O	85007	Mycobacteriales
0.00	18	0	F	1653	Corynebacteriaceae
0.00	18	5	G	1716	Corynebacterium
0.00	5	5	S	1705	Corynebacterium stationis
0.00	3	3	S	38304	Corynebacterium tuberculostearicum
0.00	2	2	S	1718	Corynebacterium glutamicum
0.00	2	2	S	169292	Corynebacterium aurimucosum
0.00	1	0	S	43771	Corynebacterium urealyticum

0.00	1	1	S1	1267754	Corynebacterium urealyticum DSM 7111
0.00	3	0	F	1762	Mycobacteriaceae
0.00	3	0	G	670516	Mycobacteroides
0.00	3	0	S	36809	Mycobacteroides abscessus
0.00	3	3	S1	1185650	Mycobacteroides abscessus subsp. abscessus
0.00	2	0	F	85025	Nocardiaceae
0.00	1	0	G	1817	Nocardia
0.00	1	1	S	135487	Nocardia cyriacigeorgica
0.00	1	0	G	3259750	Rhodococcoides
0.00	1	1	S	1828	Rhodococcoides fascians
0.00	2	0	F	85026	Gordoniaceae
0.00	2	0	G	2053	Gordonia
0.00	2	0	G1	2657482	unclassified Gordonia (in: high G+C Gram-positive bacteria)
0.00	2	2	S	337191	Gordonia sp. KTR9
0.00	1	0	F	85029	Dietziaceae
0.00	1	0	G	37914	Dietzia
0.00	1	1	S	139021	Dietzia psychralcaliphila
0.00	3	0	O	1643682	Geodermatophilales
0.00	3	0	F	85030	Geodermatophilaceae
0.00	2	0	G	38501	Blastococcus
0.00	1	1	S	1564165	Blastococcus brunescens
0.00	1	0	G1	2619396	unclassified Blastococcus
0.00	1	1	S	2933797	Blastococcus sp. PRF04-17
0.00	1	0	G	1860	Geodermatophilus
0.00	1	0	S	1861	Geodermatophilus obscurus
0.00	1	1	S1	526225	Geodermatophilus obscurus DSM 43160
0.00	2	0	O	85004	Bifidobacteriales
0.00	2	0	F	31953	Bifidobacteriaceae
0.00	2	0	G	1678	Bifidobacterium
0.00	1	1	S	1684	Bifidobacterium asteroides
0.00	1	1	S	33905	Bifidobacterium thermophilum
0.00	2	0	O	85008	Micromonosporales
0.00	2	1	F	28056	Micromonosporaceae
0.00	1	0	G	1873	Micromonospora
0.00	1	1	S	2749844	Micromonospora ferruginea
0.00	2	0	O	85012	Streptosporangiales
0.00	1	0	F	2004	Streptosporangiaceae

0.00	1	0	G	147067	Thermobispora
0.00	1	1	S	2006	Thermobispora bispora
0.00	1	0	F	2012	Thermomonosporaceae
0.00	1	0	G	1988	Actinomadura
0.00	1	0	G1	2626254	unclassified Actinomadura
0.00	1	1	S	2742128	Actinomadura sp. NAK00032
0.00	1	0	O	85010	Pseudonocardiales
0.00	1	0	F	2070	Pseudonocardiaceae
0.00	1	0	G	43356	Kutzneria
0.00	1	1	S	1483604	Kutzneria chonburiensis
0.00	1	0	O	2039638	Candidatus Nanopelagicales
0.00	1	0	F	2162846	Candidatus Nanopelagicaceae
0.00	1	1	G	622681	Candidatus Planktophila
0.00	8	0	C	84998	Coriobacteriia
0.00	5	0	O	84999	Coriobacteriales
0.00	5	5	F	1643824	Atopobiaceae
0.00	3	0	O	1643822	Eggerthellales
0.00	3	3	F	1643826	Eggerthellaceae
0.00	2	0	C	84992	Acidimicrobiia
0.00	2	0	O	84993	Acidimicrobiales
0.00	2	0	F	633392	Iamiaceae
0.00	1	0	G	467975	Iamia
0.00	1	0	G1	2624035	unclassified Iamia
0.00	1	1	S	2722752	Iamia sp. SCSIO 61187
0.00	1	0	G	1648491	Aquihabitans
0.00	1	0	G1	2633173	unclassified Aquihabitans
0.00	1	1	S	2849779	Aquihabitans sp. G128
0.00	2	0	C	1497346	Thermoleophilia
0.00	2	0	O	588673	Solirubrobacterales
0.00	1	0	F	320583	Conexibacteraceae
0.00	1	0	G	191494	Conexibacter
0.00	1	0	G1	2627773	unclassified Conexibacter
0.00	1	1	S	2937800	Conexibacter sp. S30A1
0.00	1	0	F	2600303	Baekduiaceae
0.00	1	1	G	2600304	Baekduia
0.00	27	0	P	544448	Mycoplasmatota
0.00	19	0	O	2790996	Mycoplasmoidales
0.00	19	0	F	2895623	Metamycoplasmataceae

0.00	11	0	G	2923352	Mesomycoplasma
0.00	9	0	S	2118	[Mycoplasma] mobile
0.00	9	9	S1	267748	Mycoplasma mobile 163K
0.00	2	2	S	2120	Mesomycoplasma neurolyticum
0.00	8	0	G	2767358	Mycoplasmopsis
0.00	4	4	S	171284	Mycoplasmopsis cynos
0.00	1	1	S	171279	Mycoplasmopsis anatis
0.00	1	1	S	29556	Mycoplasmopsis gallinacea
0.00	1	1	S	114881	Mycoplasmopsis columbina
0.00	1	1	S	55603	Mycoplasmopsis caviae
0.00	7	0	C	31969	Mollicutes
0.00	6	0	O	186328	Entomoplasmatales
0.00	3	0	F	2131	Spiroplasmataceae
0.00	3	0	G	2132	Spiroplasma
0.00	2	1	G1	2637901	unclassified Spiroplasma
0.00	1	1	S	3066276	Spiroplasma endosymbiont of Aspidapion aeneum
0.00	1	0	S	2137	Spiroplasma apis
0.00	1	1	S1	1276258	Spiroplasma apis B31
0.00	3	0	F	33925	Entomoplasmataceae
0.00	3	0	G	46239	Mesoplasma
0.00	1	0	S	2151	Mesoplasma florum
0.00	1	1	S1	265311	Mesoplasma florum L1
0.00	1	1	S	81459	Mesoplasma melaleucae
0.00	1	1	S	216427	Mesoplasma chauliocola
0.00	1	0	O	2085	Mycoplasmatales
0.00	1	0	F	2092	Mycoplasmataceae
0.00	1	0	G	2093	Mycoplasma
0.00	1	0	G1	656088	Mycoplasma mycoides group
0.00	1	0	S	2095	Mycoplasma capricolum
0.00	1	1	S1	40479	Mycoplasma capricolum subsp. capricolum
0.00	1	0	C	1912503	Candidatus Izimaplasma
0.00	1	0	O	2975519	Candidatus Izemoplasmatales
0.00	1	0	F	3078170	Hujiaoplasmataceae
0.00	1	0	G	3078171	Hujiaoplasma
0.00	1	1	S	2725268	Hujiaoplasma nucleasis
0.00	7	0	P	1297	Deinococcota
0.00	7	0	C	188787	Deinococci
0.00	6	0	O	118964	Deinococcales

0.00	6	0	F	183710	Deinococcaceae
0.00	6	4	G	1298	Deinococcus
0.00	1	0	G1	2623546	unclassified Deinococcus
0.00	1	1	S	2939437	Deinococcus sp. QL22
0.00	1	1	S	980427	Deinococcus wulumuqiensis
0.00	1	0	O	68933	Thermales
0.00	1	0	F	188786	Thermaceae
0.00	1	0	G	270	Thermus
0.00	1	0	S	274	Thermus thermophilus
0.00	1	1	S1	762633	Thermus thermophilus SG0.5JP17-16
0.00	4	0	P	200795	Chloroflexota
0.00	2	0	C	32061	Chloroflexia
0.00	2	0	O	32064	Chloroflexales
0.00	2	0	O1	1508594	Chloroflexineae
0.00	2	0	F	1106	Chloroflexaceae
0.00	2	2	G	1107	Chloroflexus
0.00	1	0	C	292625	Anaerolineae
0.00	1	0	O	292629	Anaerolineales
0.00	1	0	F	292628	Anaerolineaceae
0.00	1	0	G	2019482	Brevefilum
0.00	1	1	S	1986204	Brevefilum fermentans
0.00	1	0	C	475962	Caldilineae
0.00	1	0	O	475963	Caldilineales
0.00	1	0	F	475964	Caldilineaceae
0.00	1	0	G	233191	Caldilinea
0.00	1	0	S	133453	Caldilinea aerophila
0.00	1	1	S1	926550	Caldilinea aerophila DSM 14535 = NBRC 104270
0.01	274	0	D1	1783270	FCB group
0.01	270	0	D2	68336	Bacteroidota/Chlorobiota group
0.01	269	14	P	976	Bacteroidota
0.01	167	0	C	117743	Flavobacteriia
0.01	167	5	O	200644	Flavobacteriales
0.01	113	5	F	49546	Flavobacteriaceae
0.00	80	55	G	237	Flavobacterium
0.00	11	4	G1	196869	unclassified Flavobacterium
0.00	1	1	S	3031997	Flavobacterium sp. YJ01
0.00	1	1	S	2986831	Flavobacterium sp. N2270
0.00	1	1	S	2748319	Flavobacterium sp. I3-2

0.00	1	1	S	2986827	Flavobacterium sp. N1994
0.00	1	1	S	2478552	Flavobacterium sp. 140616W15
0.00	1	1	S	2739062	Flavobacterium sp. M31R6
0.00	1	1	S	2732161	Flavobacterium sp. IMCC34852
0.00	3	3	S	1751056	Flavobacterium ammonificans
0.00	2	2	S	1751095	Flavobacterium ammoniigenes
0.00	2	2	S	2906076	Flavobacterium covae
0.00	2	2	S	3003260	Flavobacterium gelatinilyticum
0.00	1	1	S	986	Flavobacterium johnsoniae
0.00	1	1	S	1492737	Flavobacterium gilvum
0.00	1	1	S	2172098	Flavobacterium pallidum
0.00	1	1	S	2175091	Flavobacterium album
0.00	1	0	G1	89279	environmental samples
0.00	1	1	S	165435	uncultured Flavobacterium sp.
0.00	4	0	G	104267	Tenacibaculum
0.00	2	0	G1	2635139	unclassified Tenacibaculum
0.00	2	2	S	3137860	Tenacibaculum sp. 190524A02b
0.00	1	1	S	107401	Tenacibaculum maritimum
0.00	1	1	S	2358479	Tenacibaculum singaporense
0.00	3	0	G	52959	Polaribacter
0.00	2	2	S	1354726	Polaribacter huanghezhanensis
0.00	1	1	S	1888915	Polaribacter haliotis
0.00	3	1	G	104264	Cellulophaga
0.00	2	1	G1	2634405	unclassified Cellulophaga
0.00	1	1	S	1942464	Cellulophaga sp. HaHaR_3_176
0.00	2	0	G	3085669	Paenimyroides
0.00	2	2	S	2968490	Paenimyroides aestuarii
0.00	2	1	G	326319	Dokdonia
0.00	1	0	G1	2615033	unclassified Dokdonia
0.00	1	1	S	983548	Dokdonia sp. 4H-3-7-5
0.00	2	0	G	290174	Aquimarina
0.00	2	0	G1	2627091	unclassified Aquimarina
0.00	2	2	S	1714860	Aquimarina sp. BL5
0.00	2	0	G	286104	Winogradskyella
0.00	2	0	G1	2615021	unclassified Winogradskyella
0.00	1	1	S	754409	Winogradskyella sp. PG-2
0.00	1	1	S	2929510	Winogradskyella sp. MH6
0.00	2	0	G	252356	Maribacter

0.00	2	0	G1	2615042	unclassified Maribacter
0.00	2	2	S	313603	Maribacter sp. HTCC2170
0.00	1	1	G	1016	Capnocytophaga
0.00	1	0	G	2953860	Candidatus Arcticimaribacter
0.00	1	1	S	2820661	Candidatus Arcticimaribacter forsetii
0.00	1	0	G	2045416	Oceanihabitans
0.00	1	0	G1	2631961	unclassified Oceanihabitans
0.00	1	1	S	2529032	Oceanihabitans sp. IOP_32
0.00	1	0	G	1518147	Wenyingzhuangia
0.00	1	1	S	1790137	Wenyingzhuangia fucanilytica
0.00	1	0	G	561367	Salinimicrobium
0.00	1	0	G1	2643747	unclassified Salinimicrobium
0.00	1	1	S	3114359	Salinimicrobium sp. 3283s
0.00	1	0	G	291183	Lacinutrix
0.00	1	1	S	1486034	Lacinutrix venerupis
0.00	1	1	G	76831	Myroides
0.00	1	0	G	143222	Salegentibacter
0.00	1	1	S	270918	Salegentibacter mishustinae
0.00	25	4	F	2762318	Weeksellaceae
0.00	15	2	F1	2782232	Chryseobacterium group
0.00	13	5	G	59732	Chryseobacterium
0.00	8	1	G1	2593645	unclassified Chryseobacterium
0.00	3	3	S	2478663	Chryseobacterium sp. 3008163
0.00	2	2	S	2825845	Chryseobacterium sp. PCH239
0.00	1	1	S	1721091	Chryseobacterium sp. IHB B 17019
0.00	1	1	S	3138177	Chryseobacterium sp. POE27
0.00	2	0	G	308865	Elizabethkingia
0.00	1	1	S	1117645	Elizabethkingia anophelis
0.00	1	1	S	1756149	Elizabethkingia bruuniana
0.00	2	0	G	1778601	Apibacter
0.00	2	2	S	2500547	Apibacter raozihei
0.00	1	0	G	28250	Ornithobacterium
0.00	1	1	S	28251	Ornithobacterium rhinotracheale
0.00	1	1	G	501783	Cloacibacterium
0.00	22	0	F	39782	Blattabacteriaceae
0.00	22	5	G	34098	Blattabacterium
0.00	13	13	S	1653831	Blattabacterium cuenoti
0.00	2	2	S	164516	Blattabacterium clevelandi

0.00	2	0	G1	2647581	unclassified Blattabacterium
0.00	2	2	S	2715232	Blattabacterium sp. DPU
0.00	2	0	O1	313602	Flavobacteriales incertae sedis
0.00	2	0	G	336809	Candidatus Karelsulcia
0.00	2	2	S	336810	Candidatus Karelsulcia muelleri
0.00	39	0	C	768503	Cytophagia
0.00	39	3	O	768507	Cytophagales
0.00	14	0	F	1853232	Hymenobacteraceae
0.00	12	5	G	89966	Hymenobacter
0.00	5	2	G1	2615202	unclassified Hymenobacter
0.00	2	2	S	2932250	Hymenobacter sp. 5317J-9
0.00	1	1	S	2675878	Hymenobacter sp. BRD128
0.00	1	1	S	1850093	Hymenobacter nivis
0.00	1	1	S	2502781	Hymenobacter jejuensis
0.00	1	0	G	323449	Pontibacter
0.00	1	0	G1	2648980	unclassified Pontibacter
0.00	1	1	S	3074898	Pontibacter sp. G13
0.00	1	0	G	1379908	Rufibacter
0.00	1	0	G1	2639626	unclassified Rufibacter
0.00	1	1	S	1379909	Rufibacter sp. DG15C
0.00	5	0	F	563798	Cyclobacteriaceae
0.00	2	1	G	390846	Echinicola
0.00	1	1	S	2859768	Echinicola marina
0.00	1	0	G	232244	Belliella
0.00	1	0	S	232259	Belliella baltica
0.00	1	1	S1	866536	Belliella baltica DSM 15883
0.00	1	0	G	246875	Algoriphagus
0.00	1	0	G1	2641541	unclassified Algoriphagus
0.00	1	1	S	3097546	Algoriphagus sp. NG3
0.00	1	0	G	1511649	Mongoliitalea
0.00	1	1	S	2782006	Mongoliitalea daihaiensis
0.00	5	0	F	89373	Cytophagaceae
0.00	4	1	G	107	Spirosoma
0.00	2	2	S	2057025	Spirosoma pollinicola
0.00	1	1	S	564064	Spirosoma rigui
0.00	1	0	G	455076	Rhodocytophaga
0.00	1	1	S	2704465	Rhodocytophaga rosea
0.00	4	0	F	200667	Flammeovirgaceae

0.00	4	1	G	59739	Flammeovirga
0.00	2	0	G1	2637820	unclassified Flammeovirga
0.00	2	2	S	1191459	Flammeovirga sp. MY04
0.00	1	1	S	373891	Flammeovirga kamogawensis
0.00	4	0	F	1501348	Amoebophilaceae
0.00	4	1	G	273135	Candidatus Cardinium
0.00	3	2	G1	2641185	unclassified Candidatus Cardinium
0.00	1	0	S	249402	Cardinium endosymbiont of Encarsia pergandiella
0.00	1	1	S1	1231626	Cardinium endosymbiont cEper1 of Encarsia pergandiella
0.00	2	0	F	3141701	Flectobacillaceae
0.00	2	1	G	2676247	Aquirufa
0.00	1	1	S	2516559	Aquirufa antheringensis
0.00	2	0	F	2896860	Spirosomataceae
0.00	1	0	G	861914	Fibrella
0.00	1	0	S	651143	Fibrella aestuarina
0.00	1	1	S1	1166018	Fibrella aestuarina BUZ 2
0.00	1	0	G	2674996	Tellurirhabdus
0.00	1	1	S	2907205	Tellurirhabdus bombi
0.00	22	0	C	200643	Bacteroidia
0.00	20	1	O	171549	Bacteroidales
0.00	5	0	F	815	Bacteroidaceae
0.00	3	1	G	816	Bacteroides
0.00	1	1	S	28116	Bacteroides ovatus
0.00	1	1	S	2136147	Bacteroides sedimenti
0.00	2	0	G	909656	Phocaeicola
0.00	2	2	S	357276	Phocaeicola dorei
0.00	4	0	F	171550	Rikenellaceae
0.00	4	0	G	1647173	Acetobacteroides
0.00	4	0	G1	1760810	environmental samples
0.00	4	4	S	1760811	uncultured Acetobacteroides sp.
0.00	2	0	F	171552	Prevotellaceae
0.00	2	0	G	838	Prevotella
0.00	1	0	S	589436	Prevotella fusca
0.00	1	1	S1	1236517	Prevotella fusca JCM 17724
0.00	1	0	G1	2638335	unclassified Prevotella
0.00	1	1	S	2937774	Prevotella sp. E15-22
0.00	2	0	F	1970190	Salinivirgaceae

0.00	2	0	G	1970191	Salinivirga
0.00	2	2	S	1307839	Salinivirga cyanobacteriivorans
0.00	2	0	F	2005525	Tannerellaceae
0.00	2	0	G	375288	Parabacteroides
0.00	1	1	S	328812	Parabacteroides goldsteinii
0.00	1	1	S	2685834	Parabacteroides chongii
0.00	1	0	F	1853231	Odoribacteraceae
0.00	1	0	G	574697	Butyricimonas
0.00	1	1	S	1472417	Butyricimonas paravirosa
0.00	1	0	F	2005473	Muribaculaceae
0.00	1	0	G	2518495	Duncaniella
0.00	1	1	S	2518971	Duncaniella dubosii
0.00	1	0	F	2005519	Barnesiellaceae
0.00	1	0	G	1348911	Coprobacter
0.00	1	1	S	1099853	Coprobacter fastidiosus
0.00	1	0	F	2005523	Paludibacteraceae
0.00	1	0	G	346096	Paludibacter
0.00	1	0	S	185300	Paludibacter propionicigenes
0.00	1	1	S1	694427	Paludibacter propionicigenes WB4
0.00	2	0	O	1970189	Marinilabiliales
0.00	2	1	F	1471398	Prolixibacteraceae
0.00	1	0	G	2678352	Maribellus
0.00	1	1	S	2681766	Maribellus comscasis
0.00	15	0	C	1853228	Chitinophagia
0.00	15	0	O	1853229	Chitinophagales
0.00	15	1	F	563835	Chitinophagaceae
0.00	11	0	G	2812019	Pseudocnuella
0.00	11	11	S	2502779	Pseudocnuella soli
0.00	1	0	G	398041	Flavisolibacter
0.00	1	1	S	661481	Flavisolibacter ginsenosidimutans
0.00	1	0	G	2698688	Paraflavitalea
0.00	1	1	S	3076558	Paraflavitalea speifideaquila
0.00	1	1	G	79328	Chitinophaga
0.00	10	0	C	117747	Sphingobacteriia
0.00	10	0	O	200666	Sphingobacteriales
0.00	10	0	F	84566	Sphingobacteriaceae
0.00	7	0	G	84567	Pedobacter
0.00	4	0	G1	2628915	unclassified Pedobacter

0.00	2	2	S	2578106	Pedobacter sp. KBS0701
0.00	1	1	S	3140251	Pedobacter sp. FW305-3-2-15-E-R2A2
0.00	1	1	S	3234142	Pedobacter sp. WC2423
0.00	2	2	S	363852	Pedobacter ginsengisoli
0.00	1	1	S	336820	Pedobacter roseus
0.00	2	1	G	423349	Mucilaginibacter
0.00	1	1	S	652787	Mucilaginibacter mallensis
0.00	1	0	G	28453	Sphingobacterium
0.00	1	1	S	28454	Sphingobacterium multivorum
0.00	2	0	C	1937959	Saprospiria
0.00	2	0	O	1936988	Saprospirales
0.00	2	0	F	89374	Saprospiraceae
0.00	1	0	G	1007	Saprospira
0.00	1	1	S	1008	Saprospira grandis
0.00	1	0	G	365032	Aureispira
0.00	1	0	G1	2649989	unclassified Aureispira
0.00	1	1	S	3051121	Aureispira sp. CCB-E
0.00	1	0	P	1853220	Rhodothermota
0.00	1	0	C	1853222	Rhodothermia
0.00	1	0	O	1853224	Rhodothermales
0.00	1	0	F	1853225	Salinibacteraceae
0.00	1	0	G	146918	Salinibacter
0.00	1	1	S	146919	Salinibacter ruber
0.00	4	0	P	142182	Gemmatimonadota
0.00	4	0	C	219685	Gemmatimonadia
0.00	4	0	O	219686	Gemmatimonadales
0.00	4	0	F	219687	Gemmatimonadaceae
0.00	4	0	G	3237678	Pseudogemmatithrix
0.00	4	4	S	3062599	Pseudogemmatithrix spongiicola
0.00	37	0	P	200940	Thermodesulfobacteriota
0.00	33	0	C	3031449	Desulfovibrionia
0.00	33	0	O	213115	Desulfovibrionales
0.00	33	7	F	194924	Desulfovibrionaceae
0.00	23	9	G	872	Desulfovibrio
0.00	13	0	G1	2593640	unclassified Desulfovibrio
0.00	13	13	S	631220	Desulfovibrio sp. G11
0.00	1	1	S	44742	Desulfovibrio fairfieldensis
0.00	2	0	G	1433996	Desulfobaculum

0.00	2	2	S	376490	Desulfobaculum bizertense
0.00	1	0	G	2035811	Pseudodesulfovibrio
0.00	1	1	S	2810563	Pseudodesulfovibrio sediminis
0.00	2	0	C	3031647	Desulfobaccia
0.00	2	0	O	3031655	Desulfobaccales
0.00	2	0	F	3031656	Desulfobaccaceae
0.00	2	0	G	60892	Desulfobacca
0.00	2	0	S	60893	Desulfobacca acetoxidans
0.00	2	2	S1	880072	Desulfobacca acetoxidans DSM 11109
0.00	1	0	C	3024418	Desulfobacteria
0.00	1	0	O	213118	Desulfobacterales
0.00	1	0	F	213119	Desulfobacteraceae
0.00	1	0	G	2289	Desulfobacter
0.00	1	0	G1	240138	environmental samples
0.00	1	1	S	240139	uncultured Desulfobacter sp.
0.00	1	1	C	3031651	Desulfuromonadia
0.00	34	1	D1	1783257	PVC group
0.00	18	0	P	203682	Planctomycetota
0.00	18	3	C	203683	Planctomycetia
0.00	9	0	O	2691355	Gemmatales
0.00	9	3	F	1914233	Gemmataceae
0.00	4	0	G	2731450	Limnoglobus
0.00	4	4	S	2598579	Limnoglobus roseus
0.00	1	1	G	113	Gemmata
0.00	1	0	G	2807415	Urbifossiella
0.00	1	1	S	2528023	Urbifossiella limnaea
0.00	5	0	O	2691354	Pirellulales
0.00	5	0	F	2691357	Pirellulaceae
0.00	2	0	G	123	Pirellula
0.00	2	0	G1	2639138	unclassified Pirellula
0.00	2	2	S	1632865	Pirellula sp. SH-Sr6A
0.00	2	0	G	2795605	Aureliella
0.00	2	2	S	2527968	Aureliella helgolandensis
0.00	1	0	G	2795779	Rosistilla
0.00	1	1	S	1930277	Rosistilla ulvae
0.00	1	0	O	2691356	Isosphaerales
0.00	1	0	F	1763524	Isosphaeraceae
0.00	1	0	G	466152	Singulisphaera

0.00	1	0	S	466153	Singulisphaera acidiphila
0.00	1	1	S1	886293	Singulisphaera acidiphila DSM 18658
0.00	10	0	P	74201	Verrucomicrobiota
0.00	10	0	C	203494	Verrucomicrobiia
0.00	9	0	O	48461	Verrucomicrobiales
0.00	7	1	F	203557	Verrucomicrobiaceae
0.00	4	0	G	2911469	Sulfuriroseicoccus
0.00	4	4	S	2707525	Sulfuriroseicoccus oceanibius
0.00	1	0	G	2735	Verrucomicrobium
0.00	1	0	S	2736	Verrucomicrobium spinosum
0.00	1	1	S1	240016	Verrucomicrobium spinosum DSM 4136 = JCM 18804
0.00	1	1	G	518753	Luteolibacter
0.00	2	0	F	1647988	Akkermansiaceae
0.00	2	1	G	239934	Akkermansia
0.00	1	1	S	239935	Akkermansia muciniphila
0.00	1	0	O	134621	Limisphaerales
0.00	1	0	F	3050006	Fontisphaeraceae
0.00	1	0	G	3050007	Fontisphaera
0.00	1	1	S	2974023	Fontisphaera persica
0.00	4	0	P	204428	Chlamydiota
0.00	4	0	C	204429	Chlamydiia
0.00	2	0	O	51291	Chlamydiales
0.00	2	0	F	809	Chlamydiaceae
0.00	2	0	F1	1113537	Chlamydia/Chlamydophila group
0.00	2	0	G	810	Chlamydia
0.00	2	2	S	83559	Chlamydia suis
0.00	2	0	O	1963360	Parachlamydiales
0.00	1	1	F	92712	Simkaniaceae
0.00	1	0	F	689704	Candidatus Rhabdochlamydiaceae
0.00	1	0	G	292833	Candidatus Rhabdochlamydia
0.00	1	1	S	225148	Candidatus Rhabdochlamydia porcellionis
0.00	1	0	P	256845	Lentisphaerota
0.00	1	0	C	1313211	Lentisphaeria
0.00	1	0	O	278081	Lentisphaerales
0.00	1	0	F	566277	Lentisphaeraceae
0.00	1	0	G	256846	Lentisphaera
0.00	1	1	S	1658616	Lentisphaera profundi
0.00	23	0	P	32066	Fusobacteriota

0.00	23	0	C	203490	Fusobacteriia
0.00	23	0	O	203491	Fusobacteriales
0.00	14	0	F	203492	Fusobacteriaceae
0.00	13	3	G	848	Fusobacterium
0.00	6	6	S	2764326	Fusobacterium hominis
0.00	3	3	S	851	Fusobacterium nucleatum
0.00	1	0	S	861	Fusobacterium ulcerans
0.00	1	1	S1	469617	Fusobacterium ulcerans ATCC 49185
0.00	1	1	G	167639	Ilyobacter
0.00	9	1	F	1129771	Leptotrichiaceae
0.00	5	2	G	32067	Leptotrichia
0.00	2	2	S	157687	Leptotrichia wadei
0.00	1	0	G1	2633022	unclassified Leptotrichia
0.00	1	1	S	712357	Leptotrichia sp. oral taxon 212
0.00	2	0	G	34104	Streptobacillus
0.00	2	2	S	34105	Streptobacillus moniliformis
0.00	1	0	G	32068	Sebaldella
0.00	1	0	S	826	Sebaldella termitidis
0.00	1	1	S1	526218	Sebaldella termitidis ATCC 33386
0.00	19	0	P	29547	Campylobacterota
0.00	19	0	C	3031852	Epsilonproteobacteria
0.00	18	0	O	213849	Campylobacterales
0.00	7	0	F	72294	Campylobacteraceae
0.00	7	2	G	194	Campylobacter
0.00	2	0	G1	2593542	unclassified Campylobacter
0.00	2	2	S	1660078	Campylobacter sp. RM5004
0.00	1	0	S	196	Campylobacter fetus
0.00	1	1	S1	1507806	Campylobacter fetus subsp. testudinum
0.00	1	0	S	522485	Campylobacter avium
0.00	1	1	S1	522484	Campylobacter avium LMG 24591
0.00	1	1	S	1031542	Campylobacter volucris
0.00	6	0	F	72293	Helicobacteraceae
0.00	6	0	G	209	Helicobacter
0.00	4	4	S	210	Helicobacter pylori
0.00	1	1	S	29419	Helicobacter canis
0.00	1	1	S	104628	Helicobacter suis
0.00	3	0	F	2771471	Sulfurimonadaceae
0.00	3	1	G	202746	Sulfurimonas

0.00	1	1	S	317658	Sulfurimonas paralvinellae
0.00	1	1	S	2590022	Sulfurimonas lithotrophica
0.00	2	0	F	2808963	Arcobacteraceae
0.00	1	0	G	28196	Arcobacter
0.00	1	0	S	28199	Arcobacter nitrofigilis
0.00	1	1	S1	572480	Arcobacter nitrofigilis DSM 7299
0.00	1	0	G	2321111	Aliarcobacter
0.00	1	0	S	708186	Aliarcobacter trophiarum
0.00	1	1	S1	1032241	Aliarcobacter trophiarum LMG 25534
0.00	1	0	O	235899	Nautiliales
0.00	1	0	F	2795691	Nitratiruptoraceae
0.00	1	0	G	269258	Nitratiruptor
0.00	1	0	G1	2624044	unclassified Nitratiruptor
0.00	1	1	S	2724901	Nitratiruptor sp. YY09-18
0.00	16	0	P	2818505	Myxococcota
0.00	9	0	C	32015	Myxococcia
0.00	9	0	O	29	Myxococcales
0.00	9	0	O1	80811	Cystobacterineae
0.00	9	9	F	31	Myxococcaceae
0.00	7	0	P1	3031711	Polyangia
0.00	7	0	O	3031712	Polyangiales
0.00	4	0	F	1524216	Labilitrichaceae
0.00	4	0	G	1524217	Labilithrix
0.00	4	4	S	1391654	Labilithrix luteola
0.00	2	0	F	49	Polyangiaceae
0.00	1	0	G	55	Polyangium
0.00	1	1	S	2567896	Polyangium aurulentum
0.00	1	0	G	39643	Sorangium
0.00	1	0	S	56	Sorangium cellulosum
0.00	1	1	S1	1254432	Sorangium cellulosum So0157-2
0.00	1	0	F	1055686	Sandaracinaceae
0.00	1	0	G	1055688	Sandaracinus
0.00	1	1	S	927083	Sandaracinus amylolyticus
0.00	6	0	P	203691	Spirochaetota
0.00	6	0	C	203692	Spirochaetia
0.00	2	1	O	136	Spirochaetales
0.00	1	0	F	2791015	Sphaerochaetaceae
0.00	1	0	G	3062336	Parasphaerochaeta

0.00	1	0	S	273376	Parasphaerochaeta coccoides
0.00	1	1	S1	760011	Parasphaerochaeta coccoides DSM 17374
0.00	2	0	O	1643687	Brevinematales
0.00	2	0	F	3024225	Thermospiraceae
0.00	2	0	G	3024226	Thermospira
0.00	2	2	S	2828656	Thermospira aquatica
0.00	1	0	O	1643686	Brachyspirales
0.00	1	0	F	143786	Brachyspiraceae
0.00	1	0	G	29521	Brachyspira
0.00	1	0	S	84378	Brachyspira murdochii
0.00	1	1	S1	526224	Brachyspira murdochii DSM 12563
0.00	1	0	O	1643688	Leptospirales
0.00	1	0	F	170	Leptospiraceae
0.00	1	0	G	338321	Turneriella
0.00	1	0	S	29510	Turneriella parva
0.00	1	1	S1	869212	Turneriella parva DSM 21527
0.00	4	2	P	57723	Acidobacteriota
0.00	1	0	C	204432	Terriglobia
0.00	1	0	O	204433	Terriglobales
0.00	1	0	F	204434	Acidobacteriaceae
0.00	1	0	G	940557	Granulicella
0.00	1	0	G1	2621151	unclassified Granulicella
0.00	1	1	S	1617967	Granulicella sp. 5B5
0.00	1	0	C	1813735	Vicinamibacteria
0.00	1	0	O	2910145	Vicinamibacterales
0.00	1	0	F	2211325	Vicinamibacteraceae
0.00	1	1	G	2004797	Luteitalea
0.00	2	0	D1	2323	Bacteria incertae sedis
0.00	2	0	D2	1783234	Bacteria candidate phyla
0.00	1	0	P	67810	Candidatus Bipolaricaulota
0.00	1	0	C	3121607	Candidatus Bipolaricaulia
0.00	1	0	O	3121608	Candidatus Bipolaricaulales
0.00	1	0	F	3121609	Candidatus Bipolaricaulaceae
0.00	1	0	G	2250122	Candidatus Bipolaricaulis
0.00	1	1	S	2026885	Candidatus Bipolaricaulis anaerobius
0.00	1	0	P	1383058	Candidatus Fervidibacterota
0.00	1	0	G	1448928	Candidatus Fervidibacter
0.00	1	1	S	1448929	Candidatus Fervidibacter sacchari

0.00	2	0	P	508458	Synergistota
0.00	2	0	C	649775	Synergistia
0.00	2	0	O	649776	Synergistales
0.00	2	0	F	3029087	Aminobacteriaceae
0.00	2	0	G	81466	Aminobacterium
0.00	2	0	G1	2685012	unclassified Aminobacterium
0.00	2	2	S	3070661	Aminobacterium sp. MB27-C1
0.00	2	0	P	200930	Deferribacterota
0.00	2	0	C	68337	Deferribacteres
0.00	2	0	O	191393	Deferribacterales
0.00	1	0	F	191394	Deferribacteraceae
0.00	1	0	G	53572	Deferribacter
0.00	1	0	S	197162	Deferribacter desulfuricans
0.00	1	1	S1	639282	Deferribacter desulfuricans SSM1
0.00	1	0	F	2945020	Mucispirillaceae
0.00	1	0	G	248038	Mucispirillum
0.00	1	0	S	248039	Mucispirillum schaedleri
0.00	1	1	S1	1379858	Mucispirillum schaedleri ASF457
0.00	1	0	P	74152	Elusimicrobiota
0.00	1	0	C	641853	Elusimicrobia
0.00	1	0	O	641854	Elusimicrobiales
0.00	1	0	F	641876	Elusimicrobiaceae
0.00	1	0	G	423604	Elusimicrobium
0.00	1	0	S	423605	Elusimicrobium minutum
0.00	1	1	S1	445932	Elusimicrobium minutum Pei191
0.00	1	0	D1	68525	delta/epsilon subdivisions
0.00	1	0	C	28221	Deltaproteobacteria
0.00	1	0	C1	45456	Deltaproteobacteria incertae sedis
0.00	1	0	G	1769732	Dissulfurimicrobium
0.00	1	1	S	1750598	Dissulfurimicrobium hydrothermale
0.00	1	0	P	67818	Atribacterota
0.00	1	0	C	2847774	Atribacteria
0.00	1	1	O	2847775	Atribacterales
0.00	1	0	P	3018035	Bdellovibrionota
0.00	1	0	C	3031418	Bdellovibrionia
0.00	1	0	O	213481	Bdellovibrionales
0.00	1	0	F	213483	Pseudobdellovibrionaceae
0.00	1	0	G	958	Bdellovibrio

0.00	1	1	S	2835041	Bdellovibrio reynosensis
0.00	29	0		2157	Archaea
0.00	18	1	P	28890	Euryarchaeota
0.00	11	0	P1	2283794	Methanomada group
0.00	10	0	C	183925	Methanobacteria
0.00	10	0	O	2158	Methanobacteriales
0.00	10	0	F	2159	Methanobacteriaceae
0.00	10	0	G	2160	Methanobacterium
0.00	8	8	S	710190	Methanobacterium petrolearium
0.00	1	1	S	710191	Methanobacterium ferruginis
0.00	1	0	G1	2627676	unclassified Methanobacterium
0.00	1	1	S	2584467	Methanobacterium sp. CWC-01
0.00	1	0	C	183939	Methanococci
0.00	1	0	O	2182	Methanococcales
0.00	1	0	F	196117	Methanocaldococcaceae
0.00	1	0	G	196118	Methanocaldococcus
0.00	1	1	S	1301915	Methanocaldococcus bathoardescens
0.00	5	0	P1	2290931	Stenosarchaea group
0.00	4	0	C	183963	Halobacteria
0.00	4	2	O	2235	Halobacteriales
0.00	2	0	F	1963268	Haloarculaceae
0.00	2	0	G	1073987	Halorientalis
0.00	2	0	G1	2648975	unclassified Halorientalis
0.00	2	2	S	1932360	Halorientalis sp. IM1011
0.00	1	0	C	224756	Methanomicrobia
0.00	1	0	O	94695	Methanosarcinales
0.00	1	0	F	2206	Methanosarcinaceae
0.00	1	0	G	2220	Methanolobus
0.00	1	1	S	3072978	Methanolobus sediminis
0.00	1	0	C	2545688	Candidatus Methanoliparia
0.00	1	0	O	2545689	Candidatus Methanoliparales
0.00	1	0	F	2545690	Candidatus Methanoliparaceae
0.00	1	0	G	2545692	Candidatus Methanoliparum
0.00	1	0	G1	2874847	unclassified Candidatus Methanoliparum
0.00	1	1	S	2874846	Candidatus Methanoliparum sp. LAM-1
0.00	7	0	D1	1783275	TACK group
0.00	4	0	P	651137	Nitrososphaerota
0.00	4	0	C	1643678	Nitrososphaeria

0.00	3	0	O	1033996	Nitrososphaerales
0.00	3	0	F	1033997	Nitrososphaeraceae
0.00	3	0	G	1826864	Candidatus Nitrosocosmicus
0.00	2	2	S	1798806	Candidatus Nitrosocosmicus franklandus
0.00	1	1	S	1353260	Candidatus Nitrosocosmicus oleophilus
0.00	1	0	O	31932	Nitrosopumilales
0.00	1	0	F	338190	Nitrosopumilaceae
0.00	1	1	G	338191	Nitrosopumilus
0.00	3	0	P	28889	Thermoproteota
0.00	3	0	C	183924	Thermoprotei
0.00	3	0	O	2281	Sulfolobales
0.00	3	0	F	118883	Sulfolobaceae
0.00	1	0	G	2284	Sulfolobus
0.00	1	0	G1	2641160	unclassified Sulfolobus
0.00	1	1	S	2512240	Sulfolobus sp. S-194
0.00	1	0	G	12914	Acidianus
0.00	1	0	S	563177	Acidianus hospitalis
0.00	1	1	S1	933801	Acidianus hospitalis W1
0.00	1	1	G	41674	Stygiolobus
0.00	3	0	D1	1783276	DPANN group
0.00	3	1	P	1462430	Candidatus Nanohalarchaeota
0.00	1	0	C	2856052	Candidatus Nanohalobia
0.00	1	0	O	2856053	Candidatus Nanohalobiales
0.00	1	0	F	2856054	Candidatus Nanohalobiaceae
0.00	1	0	G	2856051	Candidatus Nanohalobium
0.00	1	1	S	2565781	Candidatus Nanohalobium constans
0.00	1	0	P1	2856064	Candidatus Nanohalarchaeota incertae sedis
0.00	1	0	G	3157592	Candidatus Nanohalococcus
0.00	1	1	S	2978047	Candidatus Nanohalococcus occultus
0.00	1	0	P	2283796	Candidatus Thermoplasmatota
0.00	1	0	P1	1803510	DHVE2 group
0.00	1	0	G	379546	Candidatus Aciduliprofundum
0.00	1	0	G1	2633939	unclassified Aciduliprofundum
0.00	1	1	S	673860	Aciduliprofundum sp. MAR08-339
0.01	42	0		10239	Viruses
0.00	5	0	1	2559587	Riboviria
0.00	5	0	K	2732396	Orthornavirae
0.00	5	0	P	2732407	Lenarviricota

0.00	5	0	C	2732500	Miaviricetes
0.00	5	0	O	2732504	Ourlivirales
0.00	5	0	F	2560063	Botourmiaviridae
0.00	3	0	G	2560172	Magoulivirus
0.00	1	0	S	2956162	Magoulivirus jiplasmoparae
0.00	1	1	S1	2686494	Plasmopara viticola lesion associated ourmia-like virus 26
0.00	1	0	S	2956158	Magoulivirus fiplasmoparae
0.00	1	1	S1	2686493	Plasmopara viticola lesion associated ourmia-like virus 25
0.00	1	0	S	2956156	Magoulivirus epsilonplasmoparae
0.00	1	1	S1	2686564	Plasmopara viticola lesion associated ourmia-like virus 9
0.00	1	0	G	2560226	Scleroulivirus
0.00	1	0	S	2956413	Scleroulivirus lambdaplasmoparae
0.00	1	1	S1	2686545	Plasmopara viticola lesion associated ourmia-like virus 72
0.00	1	0	G	2946822	Betabotoulivirus
0.00	1	0	S	2955434	Betabotoulivirus deltaplasmoparae
0.00	1	1	S1	2686523	Plasmopara viticola lesion associated ourmia-like virus 52
0.00	37	0		185751	Pospiviroidae
0.00	19	0	G	185756	Apscaviroid
0.00	19	19	S	12904	Grapevine yellow speckle viroid 1
0.00	18	0	G	147262	Hostuviroid
0.00	18	18	S	12893	Hop stunt viroid
