# Supplementary material for: Nanopore Workflow for Grapevine Viroid Surveillance in Kazakhstan: Bypassing rRNA Depletion Through Non-Canonical Priming
Source: Pathogens. 2025 Aug 6;14(8):782. doi: 10.3390/pathogens14080782 (PMC12388932; doi:10.3390/pathogens14080782)
Supplement: Supplementary file 1 [file pathogens-14-00782-s001.zip › Table S1.pdf]

Table S1. Total RNA quantity and quality measured by Nano-500B spectrophotometer after extraction using CTAB and further cleaning using Mini Filter DNA and Mini Filter RNA

Sample N.	Purification Stage	A260	A280	A230	A260/A280	A260/A230	Conc (ng/µL)	Total ug in 50 uL
1	After CTAB	11,03	6,49	18,38	1,70	0,60	441	-
	After Mini Filters	5,25	2,78	4,20	1,89	1,25	210	10,5
2	After CTAB	12,80	7,11	16,00	1,80	0,80	512	-
	After Mini Filters	6,40	3,30	4,27	1,94	1,50	256	12,8
3	After CTAB	14,85	9,00	29,70	1,65	0,50	594	-
	After Mini Filters	7,50	3,75	3,57	2,00	2,10	300	15
4	After CTAB	16,00	9,14	20,00	1,75	0,80	640	-
	After Mini Filters	8,20	4,00	3,91	2,05	2,10	328	16,4
5	After CTAB	10,30	6,44	22,89	1,60	0,45	412	-
	After Mini Filters	5,15	2,64	4,29	1,95	1,20	206	10,3
6	After CTAB	16,80	9,33	24,00	1,80	0,70	672	-
	After Mini Filters	8,40	4,12	3,91	2,04	2,15	336	16,8
7	After CTAB	13,50	7,71	16,88	1,75	0,80	540	-
	After Mini Filters	6,80	3,45	4,00	1,97	1,70	272	13,6
8	After CTAB	11,90	7,21	17,00	1,65	0,70	476	-
	After Mini Filters	6,00	3,00	3,16	2,00	1,90	240	12
