# Supplementary figures and images for: Nanopore Workflow for Grapevine Viroid Surveillance in Kazakhstan: Bypassing rRNA Depletion Through Non-Canonical Priming
Source: Pathogens. 2025 Aug 6;14(8):782. doi: 10.3390/pathogens14080782 (PMC12388932; doi:10.3390/pathogens14080782)

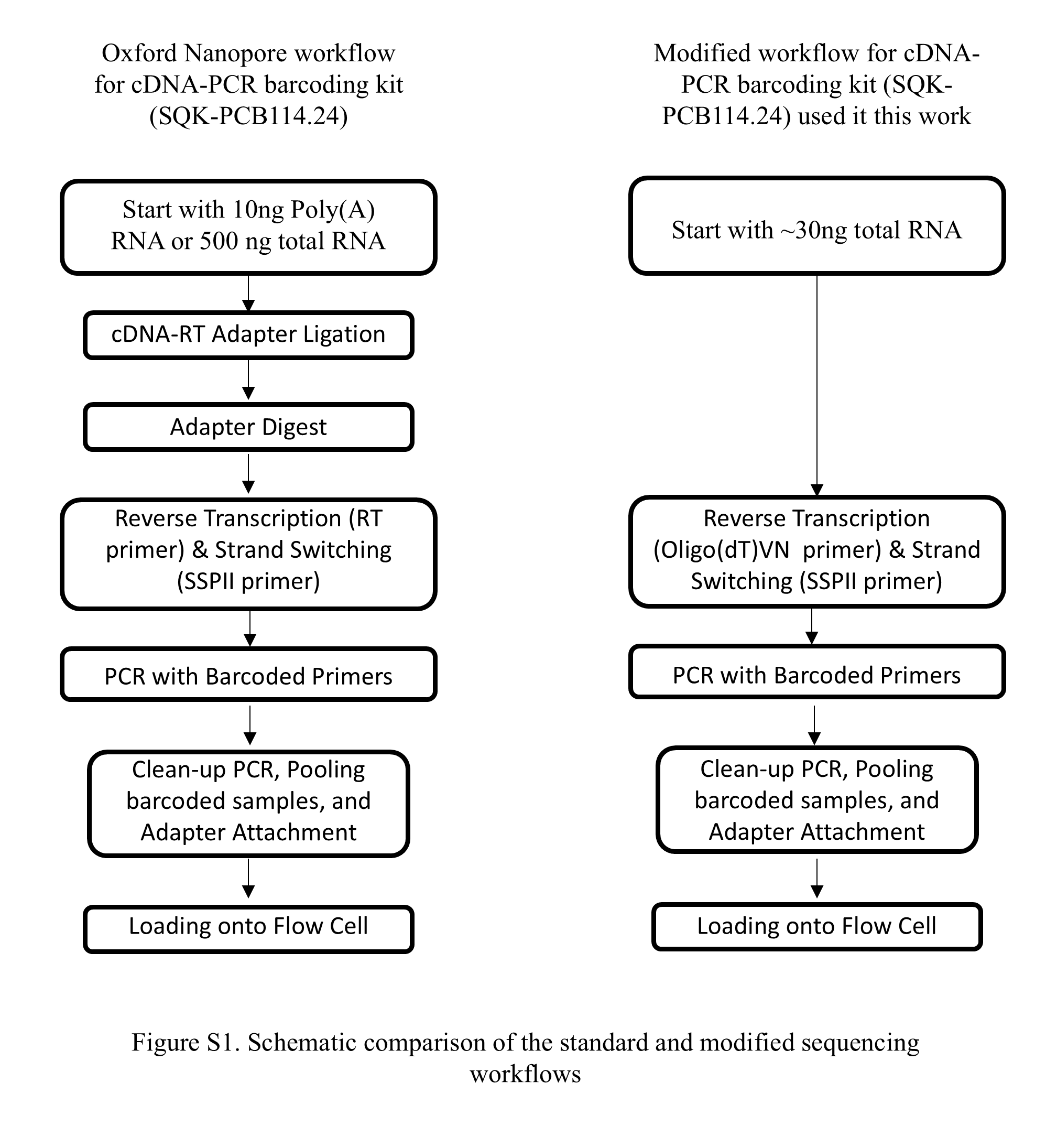

Supplement: Supplementary file 1 [file pathogens-14-00782-s001.zip › Figure S1.png]

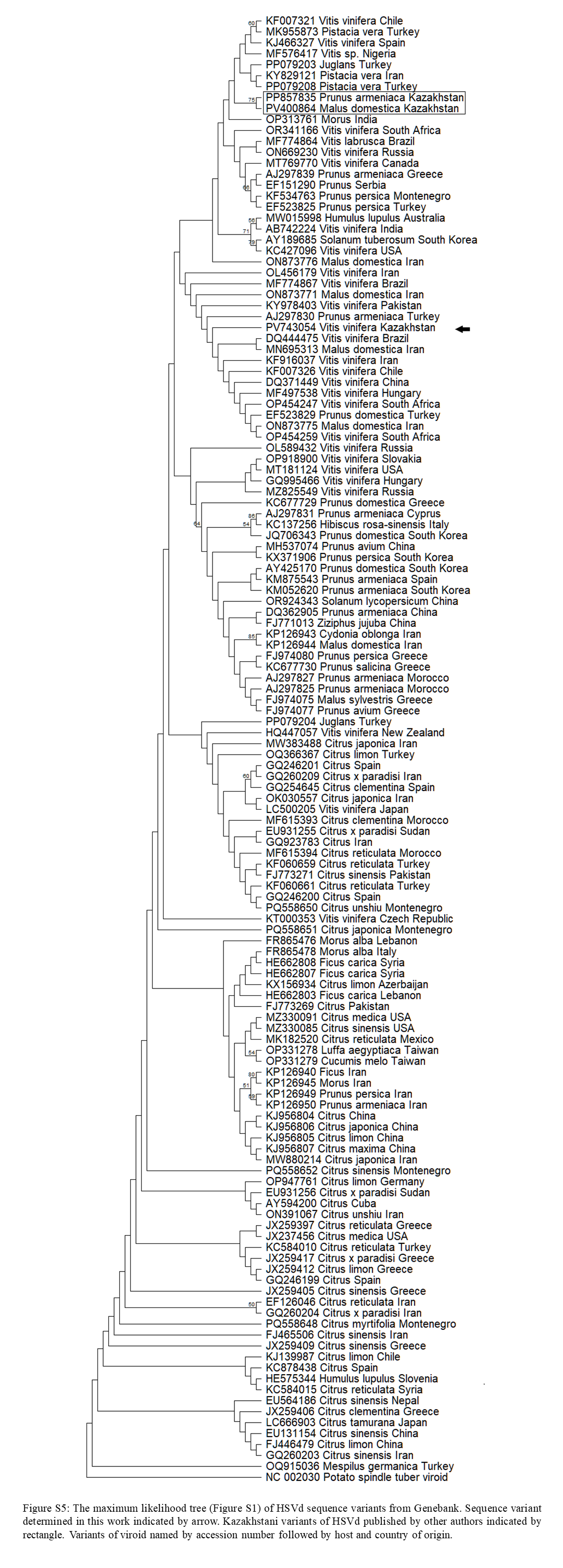

Supplement: Supplementary file 1 [file pathogens-14-00782-s001.zip › Figure S5.png]
